# Supplementary material for: Biotransformation of Methoxyflavones by Selected Entomopathogenic Filamentous Fungi
Source: Int J Mol Sci. 2020 Aug 25;21(17):6121. doi: 10.3390/ijms21176121 (PMC7503753; doi:10.3390/ijms21176121)

# Supplementary Data

## Biotransformation of methoxyflavone by selected entomopathogenic filamentous fungi

**Mateusz Łużny*, Tomasz Tronina, Ewa Kozłowska, Monika Dymarska, Jarosław Popłoński, Jacek Łyczko, Edyta Kostrzewa-Susłow and Tomasz Janeczko***

Department of Chemistry, Wrocław University of Environmental and Life Sciences, Norwida 25, 50-375 Wrocław, Poland; e.a.kozlowska@gmail.com (E.K.), ekostrzew@gmail.com (E.K-S.)

***** Correspondence:mat.luzny@gmail.com(M.Ł.) janeczko13@interia.pl (T.J.); Tel.: +48-713-205-195

## Content

Fig.S1. MS analysis flavone 2'-*O*-β-D-(4''-*O*-methyl)-glucopyranoside (**6**)
Fig.S2. $^{1}$H NMR spectral of flavone 2'-*O*-β-D-(4''-*O*-methyl)-glucopyranoside (**6**) ( DMSO-$d_6$, 600 MHz)
Fig.S3. Flavone part of the $^{1}$H NMR spectral flavone 2'-*O*-β-D-(4''-*O*-methyl)-glucopyranoside (**6**) (DMSO-$d_6$, 600 MHz)
Fig.S4. Glucopyranoside part of the $^{1}$H NMR spectral flavone 2'-*O*-β-D-(4''-*O*-methyl)-glucopyranoside (**6**) (DMSO-$d_6$, 600 MHz)
Fig.S5. $^{13}$C NMR spectral of flavone 2'-*O*-β-D-(4''-*O*-methyl)-glucopyranoside (**6**) (DMSO-$d_6$, 151 MHz)
Fig.S6. COSY spectral of flavone 2'-*O*-β-D-(4''-*O*-methyl)-glucopyranoside (**6**) (DMSO-$d_6$, 151 MHz)
Fig.S7. HMQC spectral of flavone 2'-*O*-β-D-(4''-*O*-methyl)-glucopyranoside (**6**) (DMSO-$d_6$, 151 MHz)
Fig.S8. HMBC spectral of flavone 2'-*O*-β-D-(4''-*O*-methyl)-glucopyranoside (**6**) (DMSO-$d_6$, 151 MHz)
Fig.S9. MS analysis 2'-methoxyflavone 5'-*O*-β-D-(4''-*O*-methyl)-glucopyranoside (**8**)
Fig.S10. $^{1}$H NMR spectral of 2'-methoxyflavone 5'-*O*-β-D-(4''-*O*-methyl)-glucopyranoside (**8**) (DMSO-$d_6$, 600 MHz)
Fig.S11. Flavone part of the $^{1}$H NMR spectral 2'-methoxyflavone 5'-*O*-β-D-(4''-*O*-methyl)-glucopyranoside (**8**) (DMSO-$d_6$, 600 MHz)
Fig.S12. Glucopyranoside part of the $^{1}$H NMR spectral 2'-methoxyflavone 5'-*O*-β-D-(4''-*O*-methyl)-glucopyranoside (**8**) (DMSO-$d_6$, 600 MHz)
Fig.S13. $^{13}$C NMR spectral of 2'-methoxyflavone 5'-*O*-β-D-(4''-*O*-methyl)-glucopyranoside (**8**) (DMSO-$d_6$, 151 MHz)
Fig.S14. COSY spectral of 2'-methoxyflavone 5'-*O*-β-D-(4''-*O*-methyl)-glucopyranoside (**8**) (DMSO-$d_6$, 151 MHz)
Fig.S15. HMQC spectral of 2'-methoxyflavone 5'-*O*-β-D-(4''-*O*-methyl)-glucopyranoside (**8**) (DMSO-$d_6$, 151 MHz)
Fig.S16. HMBC spectral of 2'-methoxyflavone 5'-*O*-β-D-(4''-*O*-methyl)-glucopyranoside (**8**) (DMSO-$d_6$, 151 MHz)
Fig.S17. MS analysis 2'-methoxyflavone 8-*O*-β-D-(4''-*O*-methyl)-glucopyranoside (**7**)
Fig.S18. $^{1}$H NMR spectral of 2'-methoxyflavone 8-*O*-β-D-(4''-*O*-methyl)-glucopyranoside (**7**) (Acetone-$d_6$, 600 MHz)
Fig.S19. Flavone part of the $^{1}$H NMR spectral 2'-methoxyflavone 8-*O*-β-D-(4''-*O*-methyl)-glucopyranoside (**7**) (Acetone-$d_6$, 600 MHz)
Fig.S20. $^{13}$C NMR spectral of 2'-methoxyflavone 8-*O*-β-D-(4''-*O*-methyl)-glucopyranoside (**7**) (Acetone-$d_6$, 151 MHz)
Fig.S21. COSY spectral of 2'-methoxyflavone 8-*O*-β-D-(4''-*O*-methyl)-glucopyranoside (**7**) (Acetone-$d_6$, 151 MHz)
Fig.S22. HMQC spectral of 2'-methoxyflavone 8-*O*-β-D-(4''-*O*-methyl)-glucopyranoside (**7**) (Acetone-$d_6$, 151 MHz)

Fig.S23. HMBC spectral of 2’-methoxyflavone 8-*O*-β-D-(4”-*O*-methyl)-glucopyranoside (**7**) (Acetone-$d_6$, 151 MHz)
Fig.S24. MS analysis 2’-methoxyflavone 3-*O*-β-D-(4”-*O*-methyl)-glucopyranoside (**9**)
Fig.S25. $^{1}$H NMR spectral of 2’-methoxyflavone 3-*O*-β-D-(4”-*O*-methyl)-glucopyranoside (**9**) (DMSO-$d_6$, 600 MHz)
Fig.S26. Flavone part of the $^{1}$H NMR spectral 2’-methoxyflavone 3-*O*-β-D-(4”-*O*-methyl)-glucopyranoside (**9**) (DMSO-$d_6$, 600 MHz)
Fig.S27. Glucopyranoside part of the $^{1}$H NMR spectral 2’-methoxyflavone 3-*O*-β-D-(4”-*O*-methyl)-glucopyranoside (**9**) (DMSO-$d_6$, 600 MHz)
Fig.S28. MS analysis 2’-hydroxyflavone (**10**)
Fig.S29. $^{1}$H NMR spectral of 2’-hydroxyflavone (**10**) (Acetone-$d_6$, 600 MHz)
Fig.S30. $^{13}$C NMR spectral of 2’-hydroxyflavone (**10**) (Acetone-$d_6$, 151 MHz)
Fig.S31. COSY spectral of 2’-hydroxyflavone (**10**) (Acetone-$d_6$, 151 MHz)
Fig.S32. HMQC spectral of 2’-hydroxyflavone (**10**) (Acetone-$d_6$, 151 MHz)
Fig.S33. HMBC spectral of 2’-hydroxyflavone (**10**) (Acetone-$d_6$, 151 MHz)
Fig.S34. MS analysis 3’-hydroxyflavone (**11**)
Fig.S35. $^{1}$H NMR spectral of 3’-hydroxyflavone (**11**) (DMSO-$d_6$, 600 MHz)
Fig.S36. $^{13}$C NMR spectral of 3’-hydroxyflavone (**11**) (DMSO-$d_6$, 151 MHz)
Fig.S37. COSY spectral of 3’-hydroxyflavone (**11**) (DMSO-$d_6$, 151 MHz)
Fig.S38. HMQC spectral of 3’-hydroxyflavone (**11**) (DMSO-$d_6$, 151 MHz)
Fig.S39. HMBC spectral of 3’-hydroxyflavone (**11**) (DMSO-$d_6$, 151 MHz)
Fig.S40. MS analysis flavone 3’-*O*-β-D-(4”-*O*-methyl)-glucopyranoside (**12**)
Fig.S41. $^{1}$H NMR spectral of flavone 3’-*O*-β-D-(4”-*O*-methyl)-glucopyranoside (**12**) (DMSO-$d_6$, 600 MHz)
Fig.S42. Flavone part of the $^{1}$H NMR spectral flavone 3’-*O*-β-D-(4”-*O*-methyl)-glucopyranoside (**12**) (DMSO-$d_6$, 600 MHz)
Fig.S43. Glucopyranoside part of the $^{1}$H NMR spectral flavone 3’-*O*-β-D-(4”-*O*-methyl)-glucopyranoside (**12**) (DMSO-$d_6$, 600 MHz)
Fig.S44. $^{13}$C NMR spectral of flavone 3’-*O*-β-D-(4”-*O*-methyl)-glucopyranoside (**12**) (DMSO-$d_6$, 151 MHz)
Fig.S45. HMQC spectral of flavone 3’-*O*-β-D-(4”-*O*-methyl)-glucopyranoside (**12**) (DMSO-$d_6$, 151 MHz)
Fig.S46. HMBC spectral of flavone 3’-*O*-β-D-(4”-*O*-methyl)-glucopyranoside (**12**) (DMSO-$d_6$, 151 MHz)
Fig.S47. MS analysis 4’-hydroxyflavone (**13**)
Fig.S48. $^{1}$H NMR spectral of 4’-hydroxyflavone (**13**) (DMSO-$d_6$, 600 MHz)
Fig.S49. $^{13}$C NMR spectral of 4’-hydroxyflavone (**13**) (DMSO-$d_6$, 151 MHz)
Fig.S50. COSY spectral of 4’-hydroxyflavone (**13**) (DMSO-$d_6$, 151 MHz)
Fig.S51. HMQC spectral of 4’-hydroxyflavone (**13**) (DMSO-$d_6$, 151 MHz)
Fig.S52. HMBC spectral of 4’-hydroxyflavone (**13**) (DMSO-$d_6$, 151 MHz)
Fig.S53. MS analysis flavone 4’-*O*-β-D-(4”-*O*-methyl)-glucopyranoside (**14**)
Fig.S54. $^{1}$H NMR spectral of flavone 4’-*O*-β-D-(4”-*O*-methyl)-glucopyranoside (**14**) (DMSO-$d_6$, 600 MHz)
Fig.S55. Flavone part of the $^{1}$H NMR spectral flavone 4’-*O*-β-D-(4”-*O*-methyl)-glucopyranoside (**14**) (DMSO-$d_6$, 600 MHz)
Fig.S56. Glucopyranoside part of the $^{1}$H NMR spectral flavone 4’-*O*-β-D-(4”-*O*-methyl)-glucopyranoside (**14**) (DMSO-$d_6$, 600 MHz)
Fig.S57. $^{13}$C NMR spectral of flavone 4’-*O*-β-D-(4”-*O*-methyl)-glucopyranoside (**14**) (DMSO-$d_6$, 151 MHz)
Fig.S58. COSY spectral of flavone 4’-*O*-β-D-(4”-*O*-methyl)-glucopyranoside (**14**) (DMSO-$d_6$, 151 MHz)
Fig.S59. HMQC spectral of flavone 4’-*O*-β-D-(4”-*O*-methyl)-glucopyranoside (**14**) (DMSO-$d_6$, 151 MHz)
Fig.S60. HMBC spectral of flavone 4’-*O*-β-D-(4”-*O*-methyl)-glucopyranoside (**14**) (DMSO-$d_6$, 151 MHz)
Fig.S61. MS analysis 5’-methoxyflavone 2’-*O*-β-D-(4”-*O*-methyl)-glucopyranoside (**15**)
Fig.S62. $^{1}$H NMR spectral of 5’-methoxyflavone 2’-*O*-β-D-(4”-*O*-methyl)-glucopyranoside (**15**) (DMSO-$d_6$, 600 MHz)
Fig.S63. Flavone part of the $^{1}$H NMR spectral 5’-methoxyflavone 2’-*O*-β-D-(4”-*O*-methyl)-glucopyranoside (**15**) (DMSO-$d_6$, 600 MHz)

Fig.S64. Glucopyranoside part of the $^{1}$H NMR spectral 5'-methoxyflavone 2'-*O*-β-D-(4"-*O*-methyl)-glucopyranoside (**15**) (DMSO-$d_6$, 600 MHz)
Fig.S65. $^{13}$C NMR spectral of 5'-methoxyflavone 2'-*O*-β-D-(4"-*O*-methyl)-glucopyranoside (**15**) (DMSO-$d_6$, 151 MHz)
Fig.S66. COSY spectral of 5'-methoxyflavone 2'-*O*-β-D-(4"-*O*-methyl)-glucopyranoside (**15**) (DMSO-$d_6$, 151 MHz)
Fig.S67. HMQC spectral of 5'-methoxyflavone 2'-*O*-β-D-(4"-*O*-methyl)-glucopyranoside (**15**) (DMSO-$d_6$, 151 MHz)
Fig.S68. HMBC spectral of 5'-methoxyflavone 2'-*O*-β-D-(4"-*O*-methyl)-glucopyranoside (**15**) (DMSO-$d_6$, 151 MHz)
Fig.S69. MS analysis 2',5'-dimethoxyflavone 4'-*O*-β-D-(4"-*O*-methyl)-glucopyranoside (**16**)
Fig.S70. $^{1}$H NMR spectral of 2',5'-dimethoxyflavone 4'-*O*-β-D-(4"-*O*-methyl)-glucopyranoside (**16**) (DMSO-$d_6$, 600 MHz)
Fig.S71. Flavone part of the $^{1}$H NMR spectral 2',5'-dimethoxyflavone 4'-*O*-β-D-(4"-*O*-methyl)-glucopyranoside (**16**) (DMSO-$d_6$, 600 MHz)
Fig.S72. Glucopyranoside part of the $^{1}$H NMR spectral 2',5'-dimethoxyflavone 4'-*O*-β-D-(4"-*O*-methyl)-glucopyranoside (**16**) (DMSO-$d_6$, 600 MHz)
Fig.S73. $^{13}$C NMR spectral of 2',5'-dimethoxyflavone 4'-*O*-β-D-(4"-*O*-methyl)-glucopyranoside (**16**) (DMSO-$d_6$, 151 MHz)
Fig.S74. COSY spectral of 2',5'-dimethoxyflavone 4'-*O*-β-D-(4"-*O*-methyl)-glucopyranoside (**16**) (DMSO-$d_6$, 151 MHz)
Fig.S75. HMQC spectral of 2',5'-dimethoxyflavone 4'-*O*-β-D-(4"-*O*-methyl)-glucopyranoside (**16**) (DMSO-$d_6$, 151 MHz)
Fig.S76. HMBC spectral of 2',5'-dimethoxyflavone 4'-*O*-β-D-(4"-*O*-methyl)-glucopyranoside (**16**) (DMSO-$d_6$, 151 MHz)
Fig.S77. MS analysis 5'-hydroxy-2'-methoxyflavone (**17**)
Fig.S78. $^{1}$H NMR spectral of 5'-hydroxy-2'-methoxyflavone (**17**) (DMSO-$d_6$, 600 MHz)
Fig.S79. $^{13}$C NMR spectral of 5'-hydroxy-2'-methoxyflavone (**17**) (DMSO-$d_6$, 151 MHz)
Fig.S80. COSY spectral of 5'-hydroxy-2'-methoxyflavone (**17**) (DMSO-$d_6$, 151 MHz)
Fig.S81. HMQC spectral of 5'-hydroxy-2'-methoxyflavone (**17**) (DMSO-$d_6$, 151 MHz)
Fig.S82. HMBC spectral of 5'-hydroxy-2'-methoxyflavone (**17**) (DMSO-$d_6$, 151 MHz)
Fig.S83. MS analysis 4'-hydroxy-2',5'-dimethoxyflavone
Fig.S84. $^{1}$H NMR spectral of 5'-hydroxy-2'-methoxyflavone (**17**) and 4'-hydroxy-2',5'-dimethoxyflavone (**18**) (DMSO-$d_6$, 600 MHz)
Fig.S85. Part of the $^{1}$H NMR spectral 5'-hydroxy-2'-methoxyflavone (**17**) and 4'-hydroxy-2',5'-dimethoxyflavone (**18**) (DMSO-$d_6$, 600 MHz)
Fig.S86. $^{13}$C NMR spectral of 5'-hydroxy-2'-methoxyflavone (**17**) and 4'-hydroxy-2',5'-dimethoxyflavone (**18**) (DMSO-$d_6$, 151 MHz)
Fig.S87. HMQC spectral of 5'-hydroxy-2'-methoxyflavone (**17**) and 4'-hydroxy-2',5'-dimethoxyflavone (**18**) (DMSO-$d_6$, 151 MHz)
Fig.S88. HMBC spectral of 5'-hydroxy-2'-methoxyflavone (**17**) and 4'-hydroxy-2',5'-dimethoxyflavone (**18**) (DMSO-$d_6$, 151 MHz)
Fig.S89. MS analysis 4',5'-dimethoxyflavone 5'-*O*-β-D-(4"-*O*-methyl)-glucopyranoside (**19**) and 3',5'-dimethoxyflavone 4'-*O*-β-D-(4"-*O*-methyl)-glucopyranoside (**20**)
Fig.S90. $^{1}$H NMR spectral of 4',5'-dimethoxyflavone 5'-*O*-β-D-(4"-*O*-methyl)-glucopyranoside (**19**) and 3',5'-dimethoxyflavone 4'-*O*-β-D-(4"-*O*-methyl)-glucopyranoside (**20**) (DMSO-$d_6$, 600 MHz)
Fig.S91. Flavone part of the $^{1}$H NMR spectral 4',5'-dimethoxyflavone 5'-*O*-β-D-(4"-*O*-methyl)-glucopyranoside (**19**) and 3',5'-dimethoxyflavone 4'-*O*-β-D-(4"-*O*-methyl)-glucopyranoside (**20**) (DMSO-$d_6$, 600 MHz)
Fig.S92. Glucopyranoside part of the $^{1}$H NMR spectral 4',5'-dimethoxyflavone 5'-*O*-β-D-(4"-*O*-methyl)-glucopyranoside (**19**) and 3',5'-dimethoxyflavone 4'-*O*-β-D-(4"-*O*-methyl)-glucopyranoside (**20**) (DMSO-$d_6$, 600 MHz)

Fig.S93. $^{13}$C NMR spectral of 4’,5’-dimethoxyflavone 5’-*O*-β-D-(4”-*O*-methyl)-glucopyranoside (**19**) and 3’,5’-dimethoxyflavone 4’-*O*-β-D-(4”-*O*-methyl)-glucopyranoside (**20**) (DMSO-$d_6$, 151 MHz)
Fig.S94. HMQC spectral of 4’,5’-dimethoxyflavone 5’-*O*-β-D-(4”-*O*-methyl)-glucopyranoside (**19**) and 3’,5’-dimethoxyflavone 4’-*O*-β-D-(4”-*O*-methyl)-glucopyranoside (**20**) (DMSO-$d_6$, 151 MHz)
Fig.S95. HMBC spectral of 4’,5’-dimethoxyflavone 5’-*O*-β-D-(4”-*O*-methyl)-glucopyranoside (**19**) and 3’,5’-dimethoxyflavone 4’-*O*-β-D-(4”-*O*-methyl)-glucopyranoside (**20**) (DMSO-$d_6$, 151 MHz)
Fig.S96. MS analysis 3’,4’,5’-trimethoxyflavone 6-*O*-β-D-(4”-*O*-methyl)-glucopyranoside (**21**)
Fig.S97. $^{1}$H NMR spectral of 3’,4’,5’-trimethoxyflavone 6-*O*-β-D-(4”-*O*-methyl)-glucopyranoside (**21**) (DMSO-$d_6$, 600 MHz)
Fig.S98. Flavone part of the $^{1}$H NMR spectral 3’,4’,5’-trimethoxyflavone 6-*O*-β-D-(4”-*O*-methyl)-glucopyranoside (**21**) (DMSO-$d_6$, 600 MHz)
Fig.S99. Glucopyranoside part of the $^{1}$H NMR spectral 3’,4’,5’-trimethoxyflavone 6-*O*-β-D-(4”-*O*-methyl)-glucopyranoside (**21**) (DMSO-$d_6$, 600 MHz)
Fig.S100. $^{13}$C NMR spectral of 3’,4’,5’-trimethoxyflavone 6-*O*-β-D-(4”-*O*-methyl)-glucopyranoside (**21**) (DMSO-$d_6$, 151 MHz)
Fig.S101. HMQC spectral of 3’,4’,5’-trimethoxyflavone 6-*O*-β-D-(4”-*O*-methyl)-glucopyranoside (**21**) (DMSO-$d_6$, 151 MHz)
Fig.S102. HMBC spectral of 3’,4’,5’-trimethoxyflavone 6-*O*-β-D-(4”-*O*-methyl)-glucopyranoside (**21**) (DMSO-$d_6$, 151 MHz)
Fig.S103. MS analysis 6-hydroxy-4’,5’-dimethoxyflavone 3’-*O*-β-D-(4”-*O*-methyl)-glucopyranoside (**22**)
Fig.S104. $^{1}$H NMR spectral of 6-hydroxy-4’,5’-dimethoxyflavone 3’-*O*-β-D-(4”-*O*-methyl)-glucopyranoside (**22**) (DMSO-$d_6$, 600 MHz)
Fig.S105. Flavone part of the $^{1}$H NMR spectral 6-hydroxy-4’,5’-dimethoxyflavone 3’-*O*-β-D-(4”-*O*-methyl)-glucopyranoside (**22**) (DMSO-$d_6$, 600 MHz)
Fig.S106. Glucopyranoside part of the $^{1}$H NMR spectral 6-hydroxy-4’,5’-dimethoxyflavone 3’-*O*-β-D-(4”-*O*-methyl)-glucopyranoside (**22**) (DMSO-$d_6$, 600 MHz)
Fig.S107. $^{13}$C NMR spectral of 6-hydroxy-4’,5’-dimethoxyflavone 3’-*O*-β-D-(4”-*O*-methyl)-glucopyranoside (**22**) (DMSO-$d_6$, 151 MHz)
Fig.S108. HMQC spectral of 6-hydroxy-4’,5’-dimethoxyflavone 3’-*O*-β-D-(4”-*O*-methyl)-glucopyranoside (**22**) (DMSO-$d_6$, 151 MHz)
Fig.S109. HMBC spectral of 6-hydroxy-4’,5’-dimethoxyflavone 3’-*O*-β-D-(4”-*O*-methyl)-glucopyranoside (**22**) (DMSO-$d_6$, 151 MHz)

Fig.S1. MS analysis flavone 2'-*O*-β-D-(4''-*O*-methyl)-glucopyranoside (**6**)

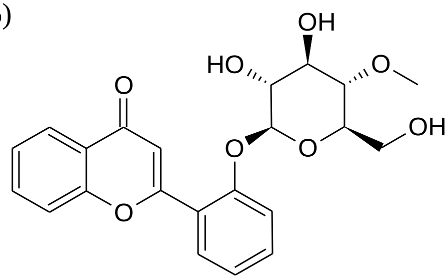


Molecular Formula = $C_{22}H_{22}O_8$
Formula Weight = 414.40528
Precursor: = 415.4000
CE (collision energy): -15.0

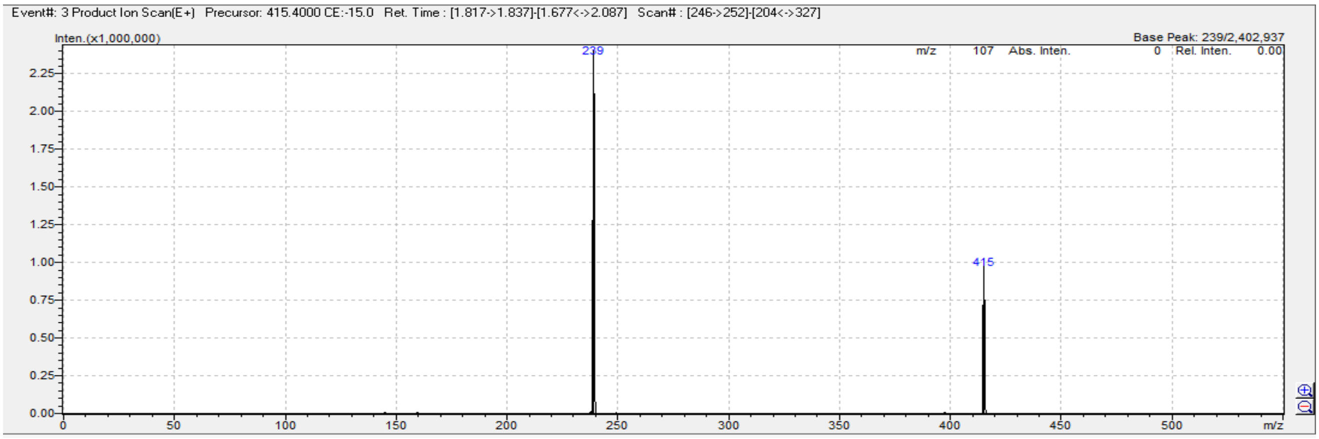


CE:-35.0

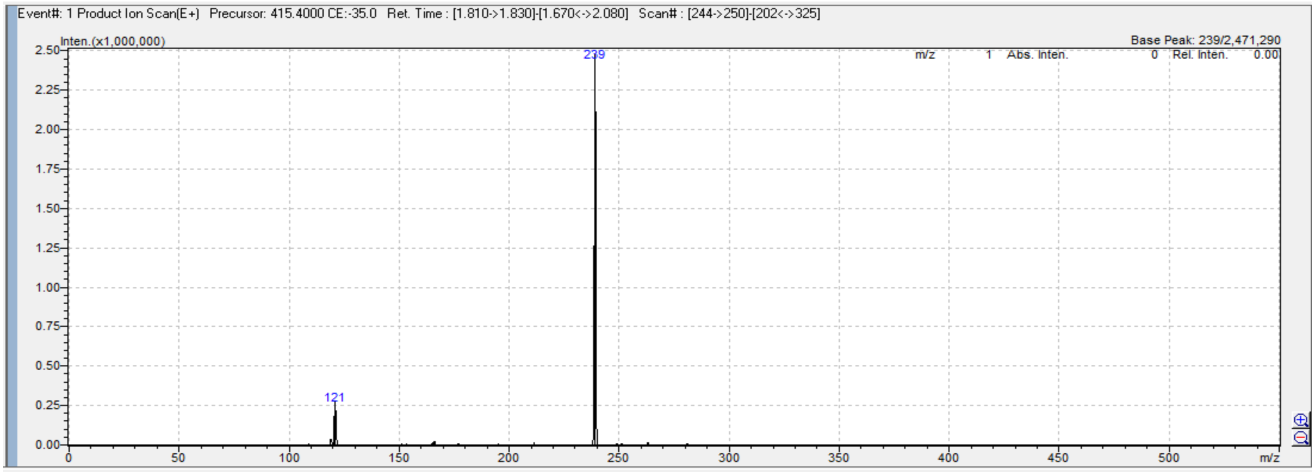


CE:-45.0

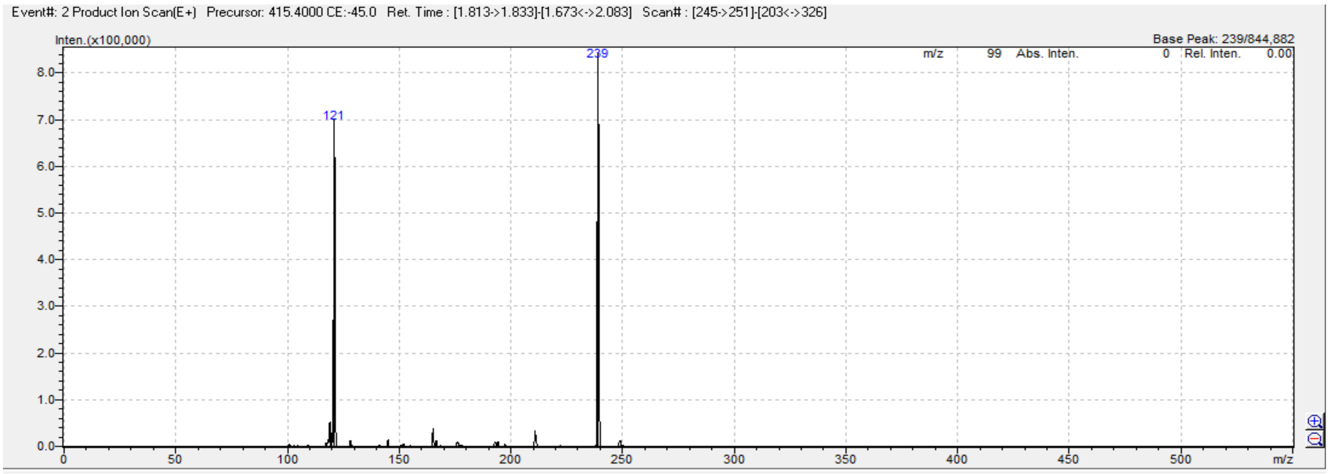

Fig.S2. $^{1}$H NMR spectral of flavone 2’-*O*-β-D-(4”-*O*-methyl)-glucopyranoside (**6**) (DMSO-$d_6$, 600 MHz)

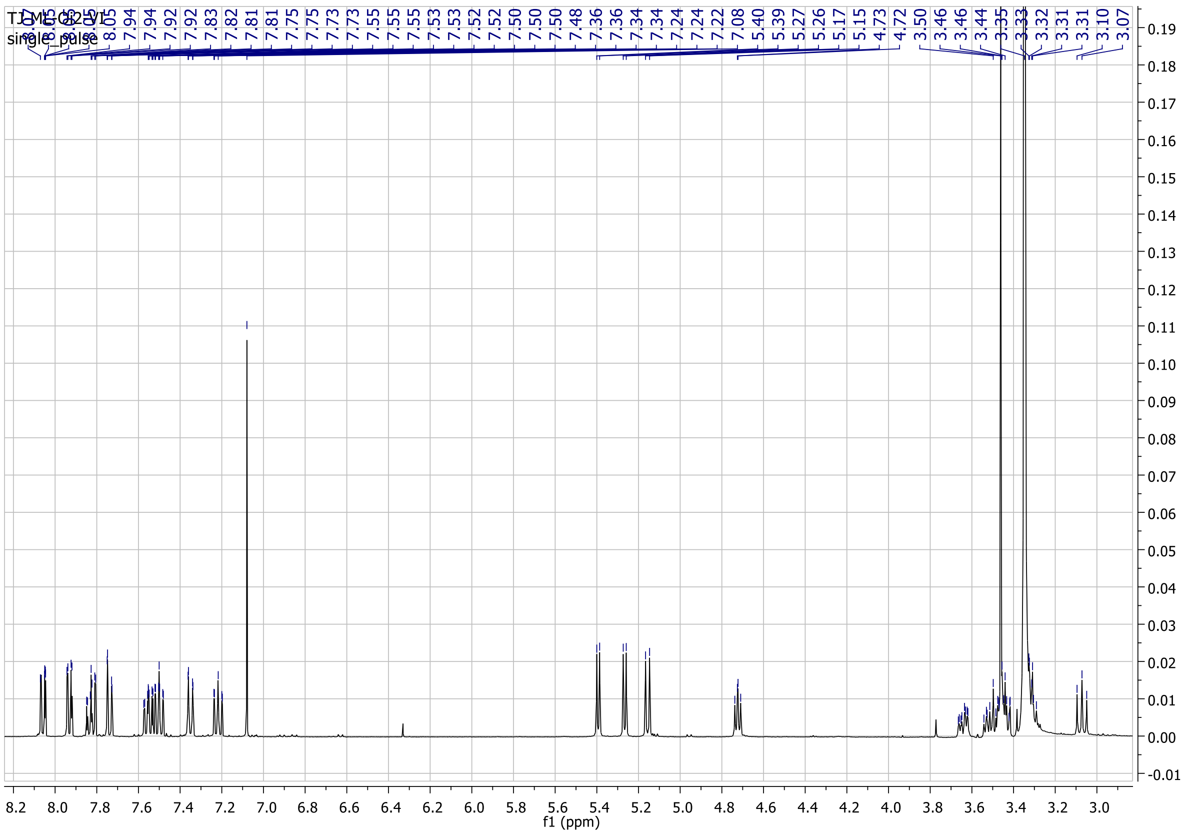


Fig.S3. Flavone part of the $^{1}$H NMR spectral flavone 2’-*O*-β-D-(4”-*O*-methyl)-glucopyranoside (**6**) (DMSO-$d_6$, 600 MHz)

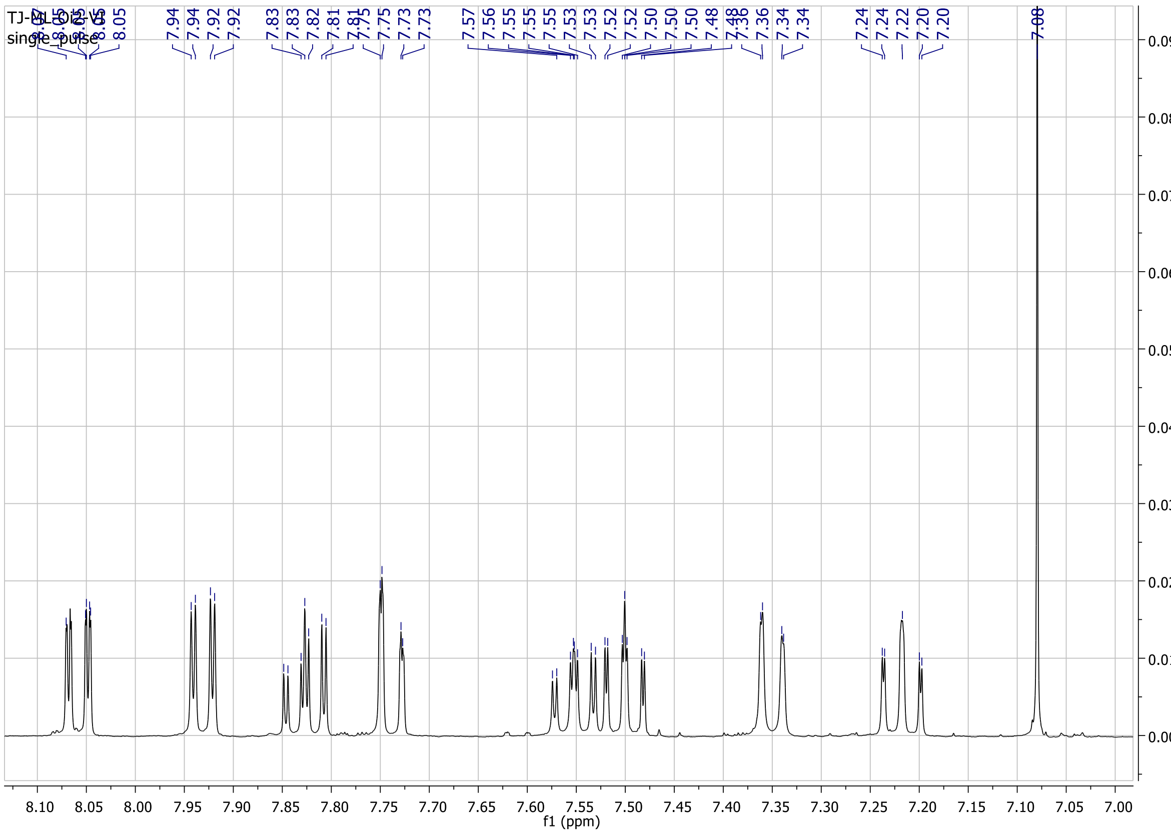

Fig.S4. Glucopyranoside part of the $^{1}$H NMR spectral flavone 2’-*O*-β-D-(4”-*O*-methyl)-glucopyranoside (**6**) (DMSO-$d_6$, 600 MHz)

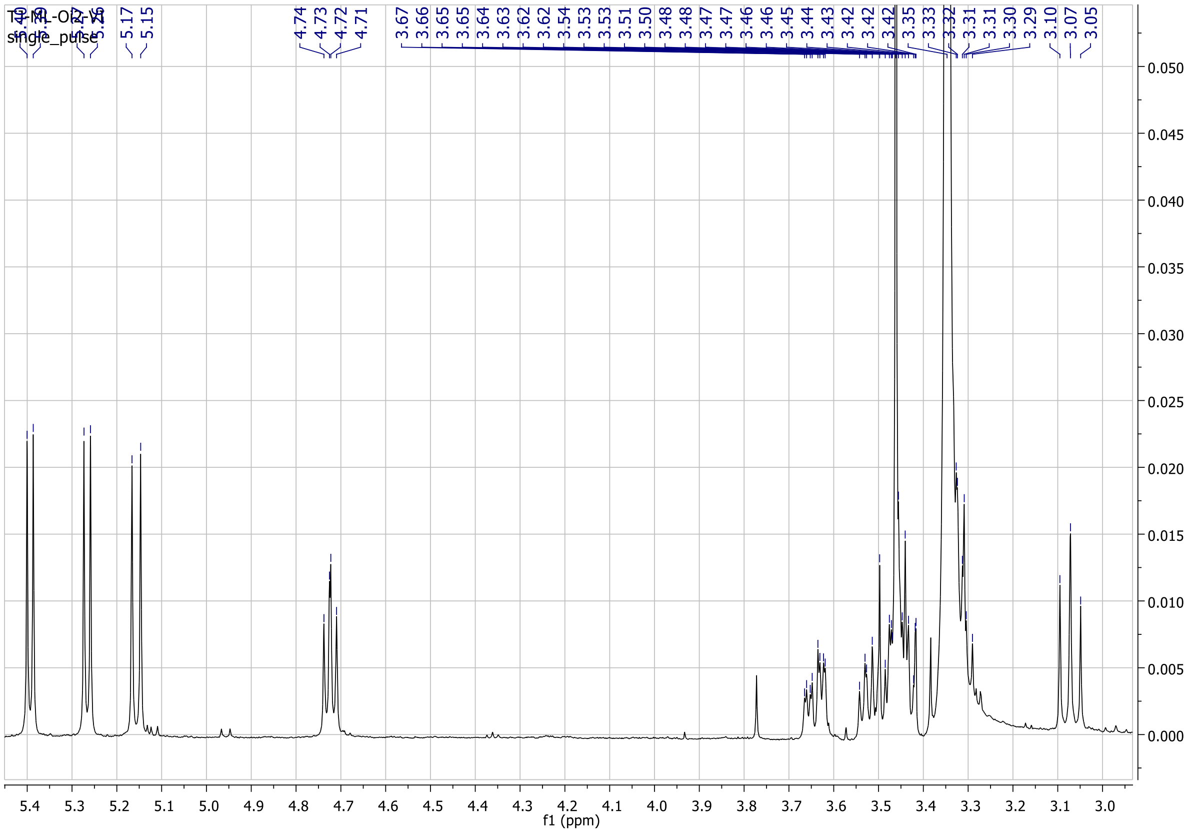


Fig.S5. $^{13}$C NMR spectral of flavone 2’-*O*-β-D-(4”-*O*-methyl)-glucopyranoside (**6**) (DMSO-$d_6$, 151 MHz)

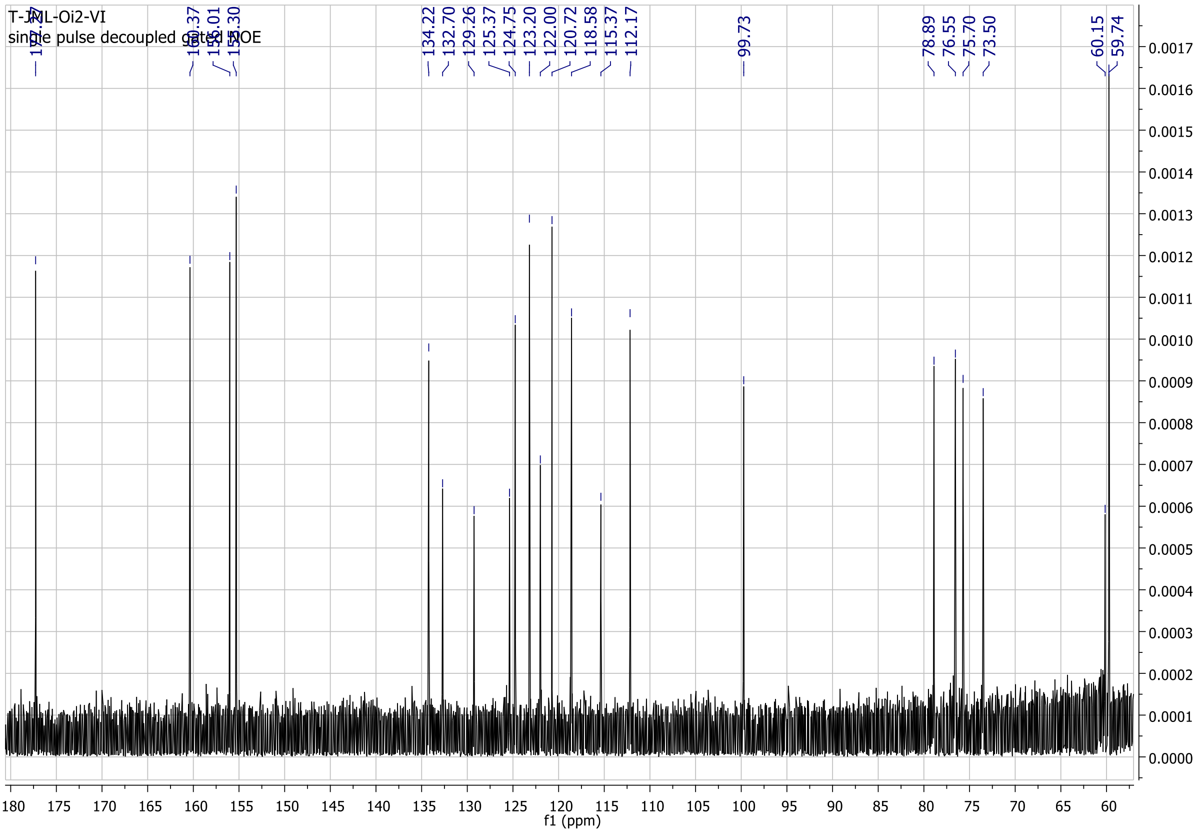

Fig.S6. COSY spectral of flavone 2'-*O*-β-D-(4''-*O*-methyl)-glucopyranoside (**6**) (DMSO-$d_6$, 151 MHz)

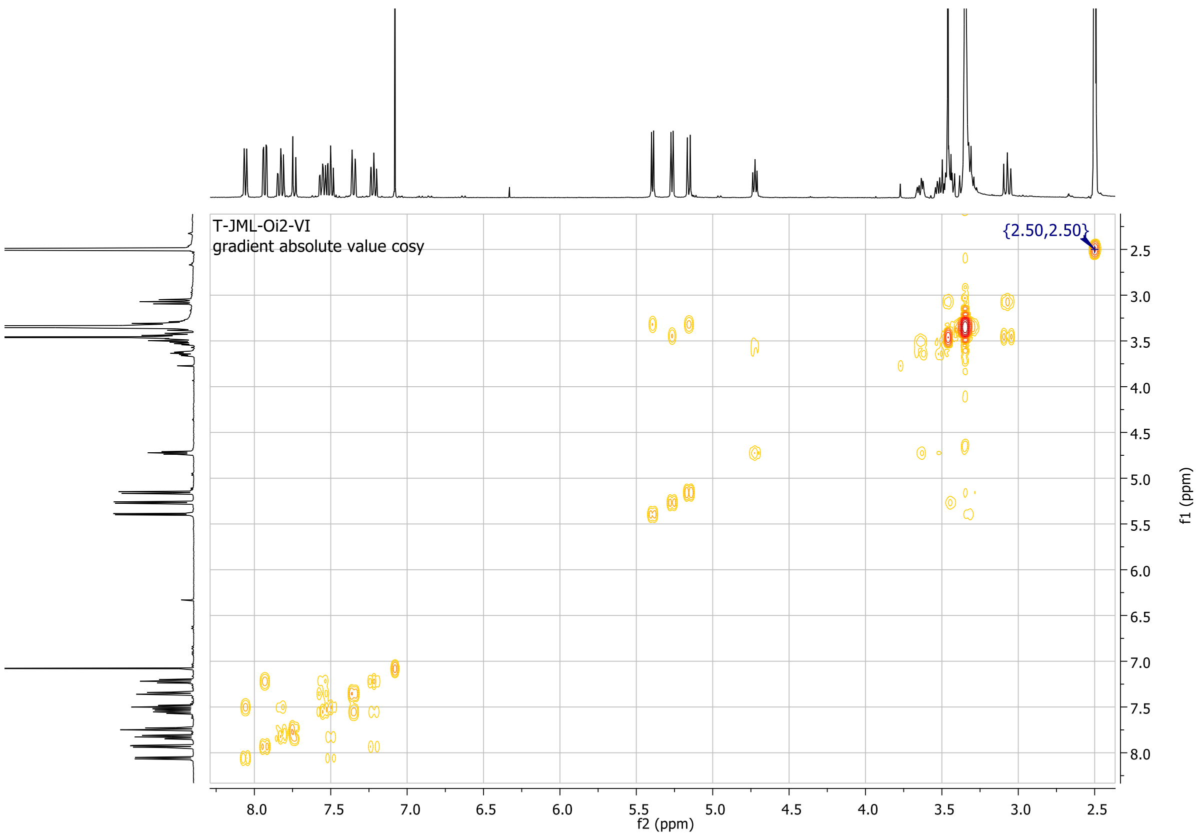


Fig.S7. HMQC spectral of flavone 2'-*O*-β-D-(4''-*O*-methyl)-glucopyranoside (**6**) (DMSO-$d_6$, 151 MHz)

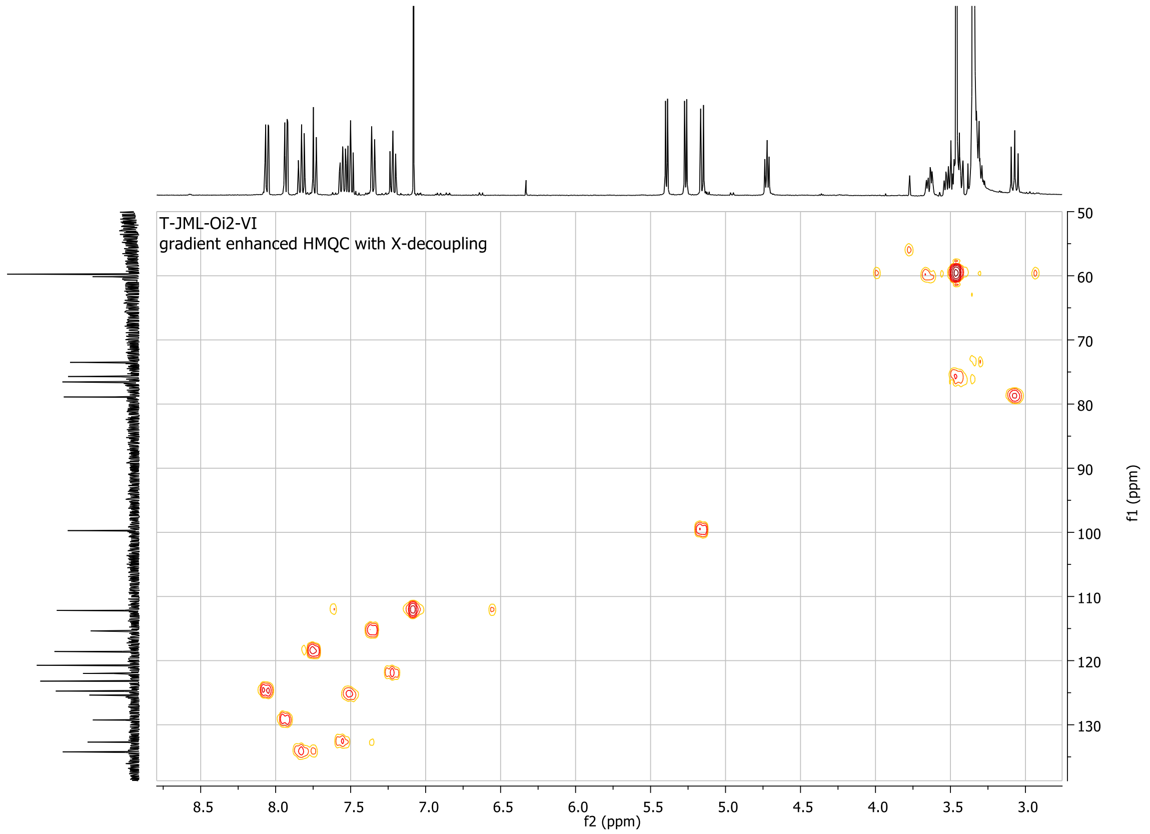

Fig.S8. HMBC spectral of flavone 2’-*O*-β-D-(4”-*O*-methyl)-glucopyranoside (**6**) (DMSO-$d_6$, 151 MHz)

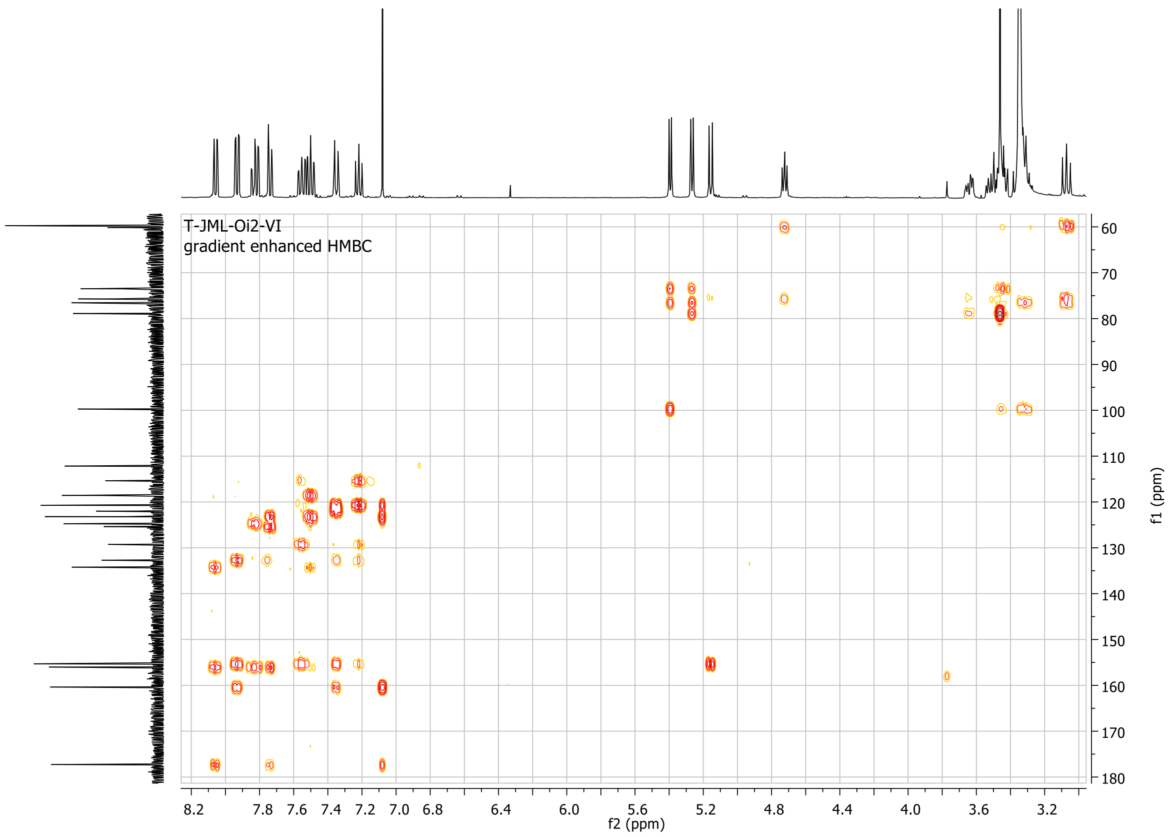

Fig.S9. MS analysis 2’-methoxyflavone 5’-*O*-β-D-(4”-*O*-methyl)-glucopyranoside (**8**)

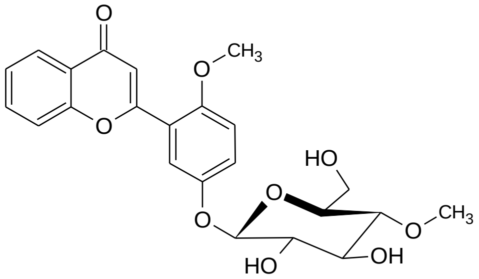


Molecular Formula = $C_{23}H_{24}O_9$

Formula Weight = 444.43126

Precursor =445.4000

CE: -15.0

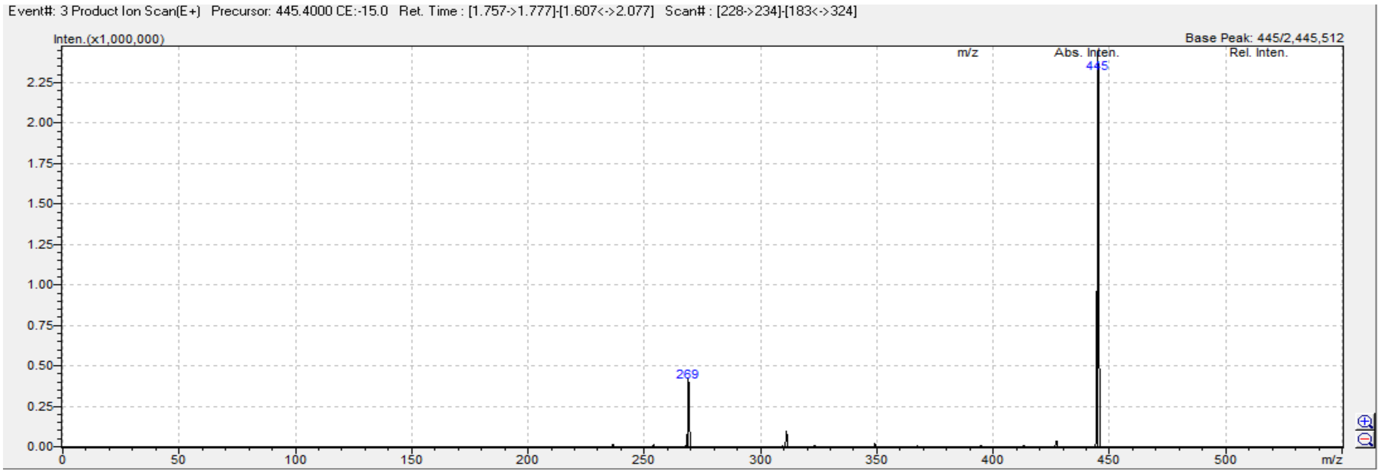


CE:-35.0

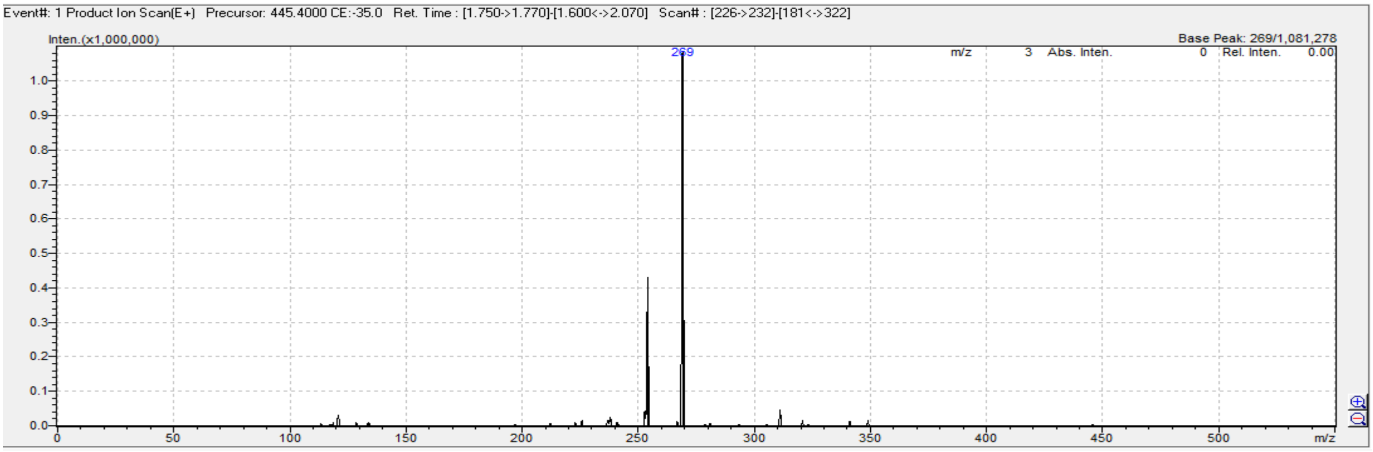


CE:-45.0

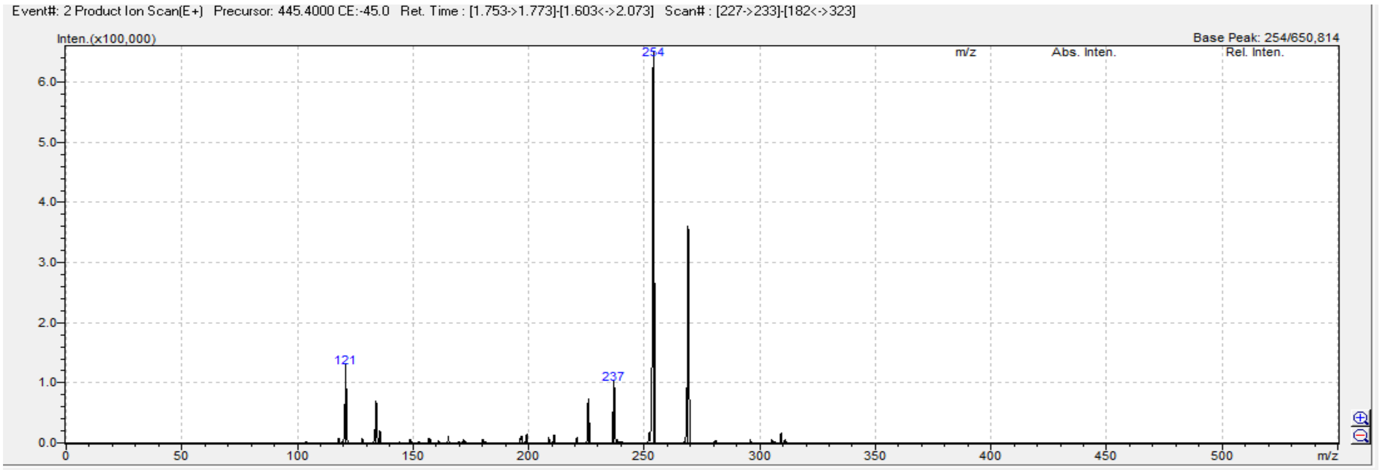

Fig.S10. $^{1}$H NMR spectral of 2’-methoxyflavone 5’-*O*-β-D-(4”-*O*-methyl)-glucopyranoside (**8**) (Acetone-$d_6$, 600 MHz)

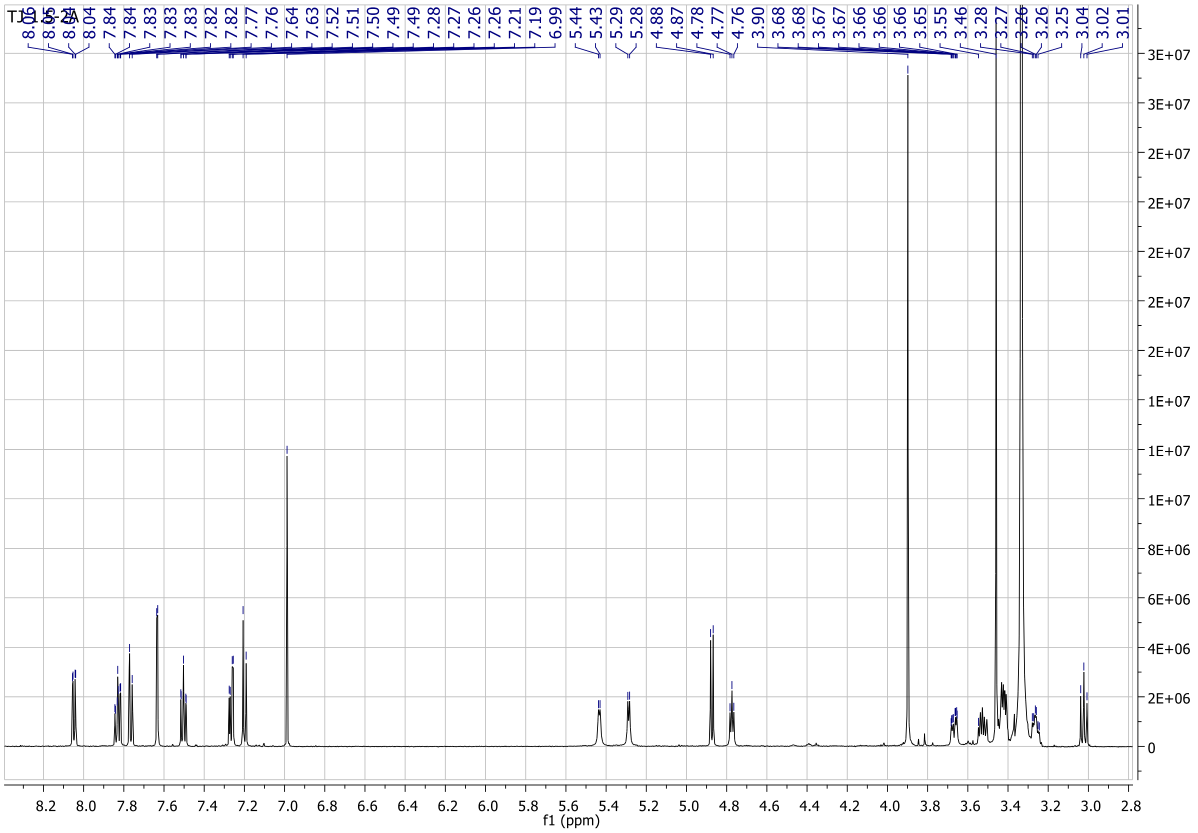


Fig.S11. Flavone part of the $^{1}$H NMR spectral 2’-methoxyflavone 5’-*O*-β-D-(4”-*O*-methyl)-glucopyranoside (**8**) (Acetone-$d_6$, 600 MHz)

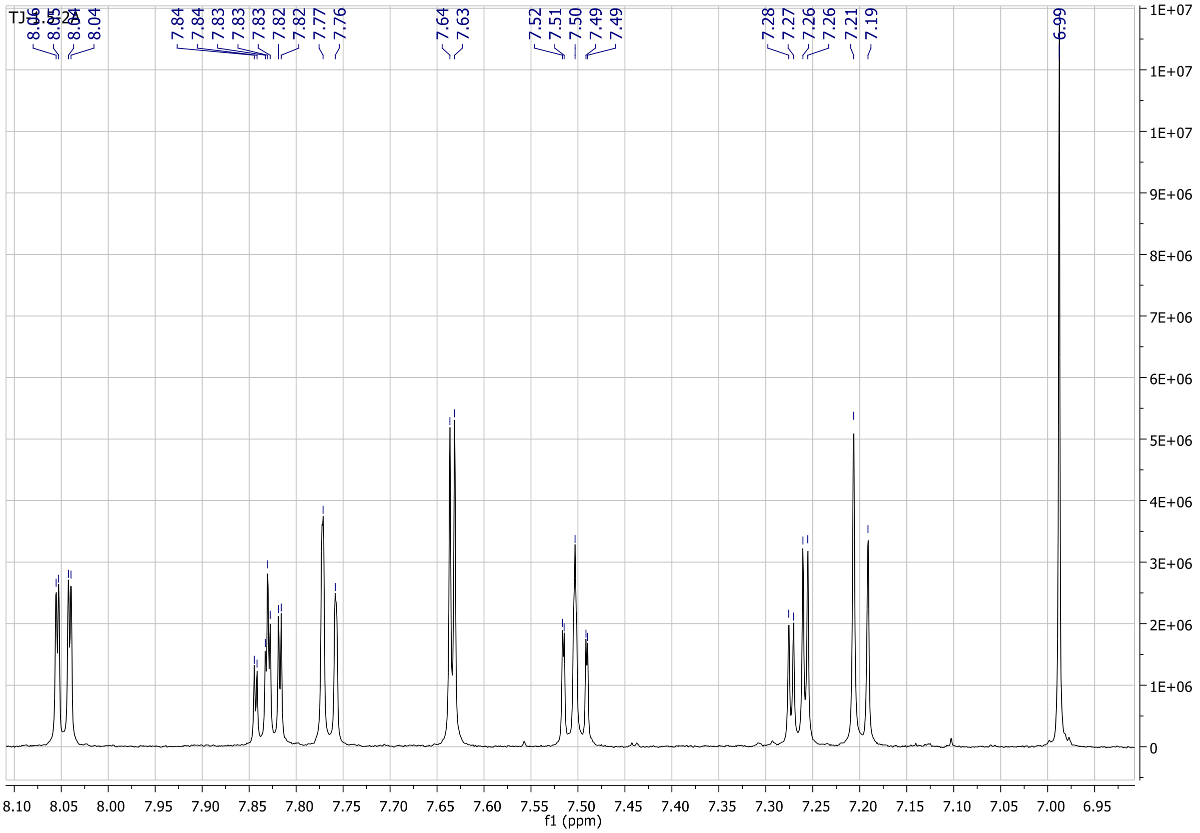

Fig.S12. Glucopyranoside part of the $^{1}H$ NMR spectral 2’-methoxyflavone 5’-*O*-β-D-(4”-*O*-methyl)-glucopyranoside (**8**) (Acetone-$d_6$, 600 MHz)

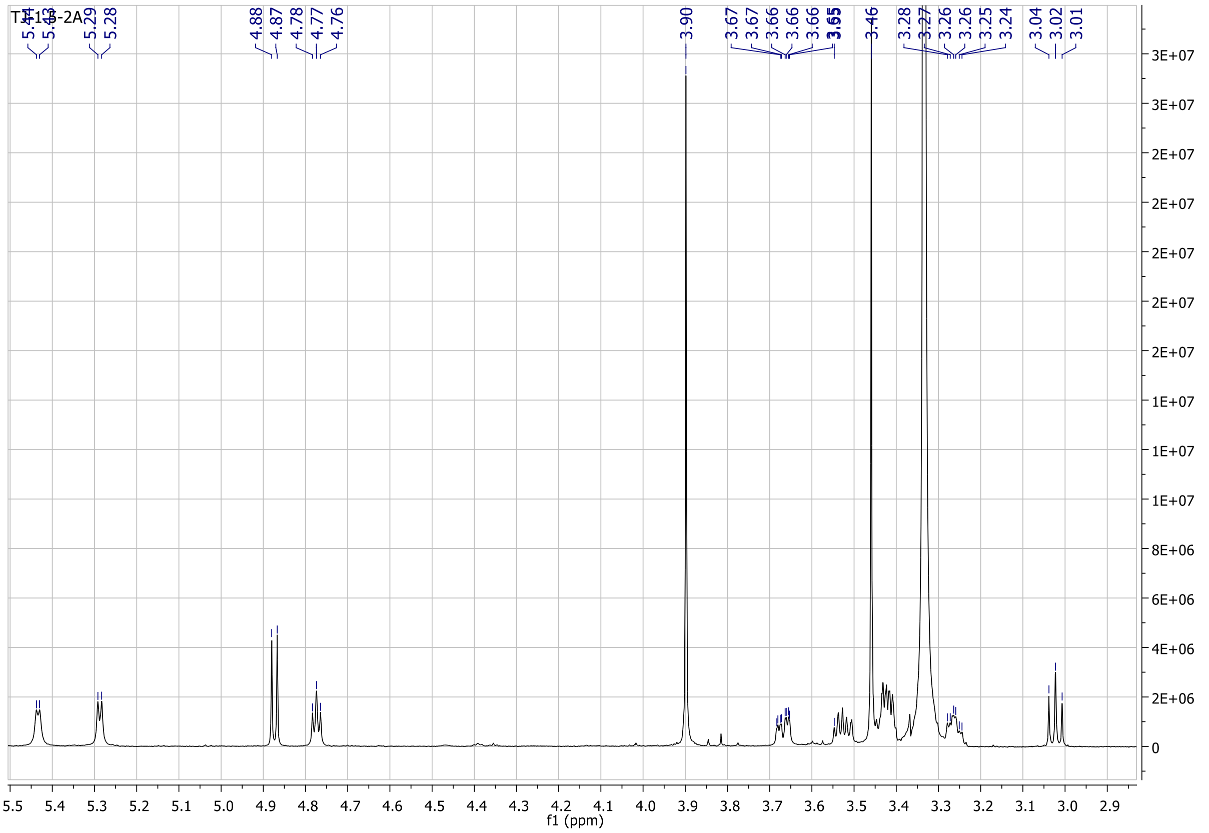


Fig.S13. $^{13}C$ NMR spectral of 2’-methoxyflavone 5’-*O*-β-D-(4”-*O*-methyl)-glucopyranoside (**8**) (Acetone-$d_6$, 151 MHz)

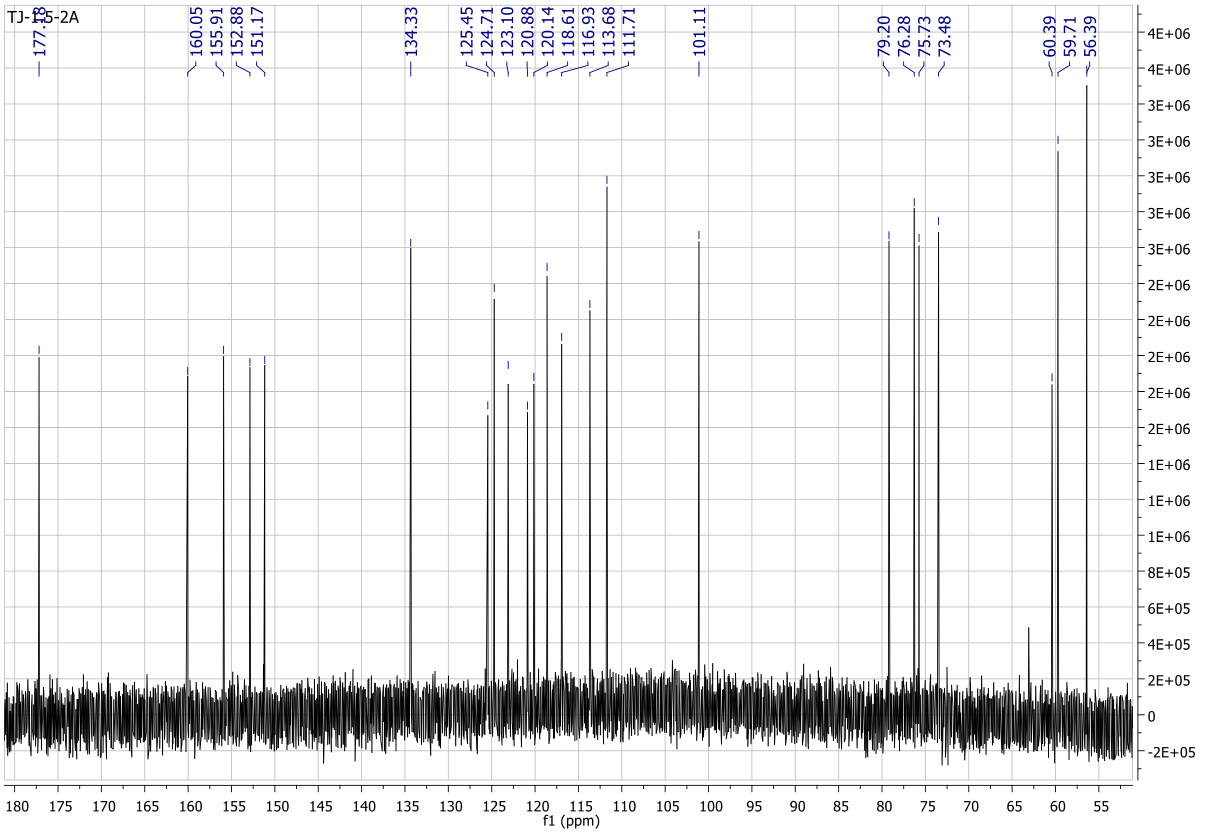

Fig.S14. COSY spectral of 2'-methoxyflavone 5'-*O*-β-D-(4''-*O*-methyl)-glucopyranoside (**8**) (Acetone-$d_6$, 151 MHz)

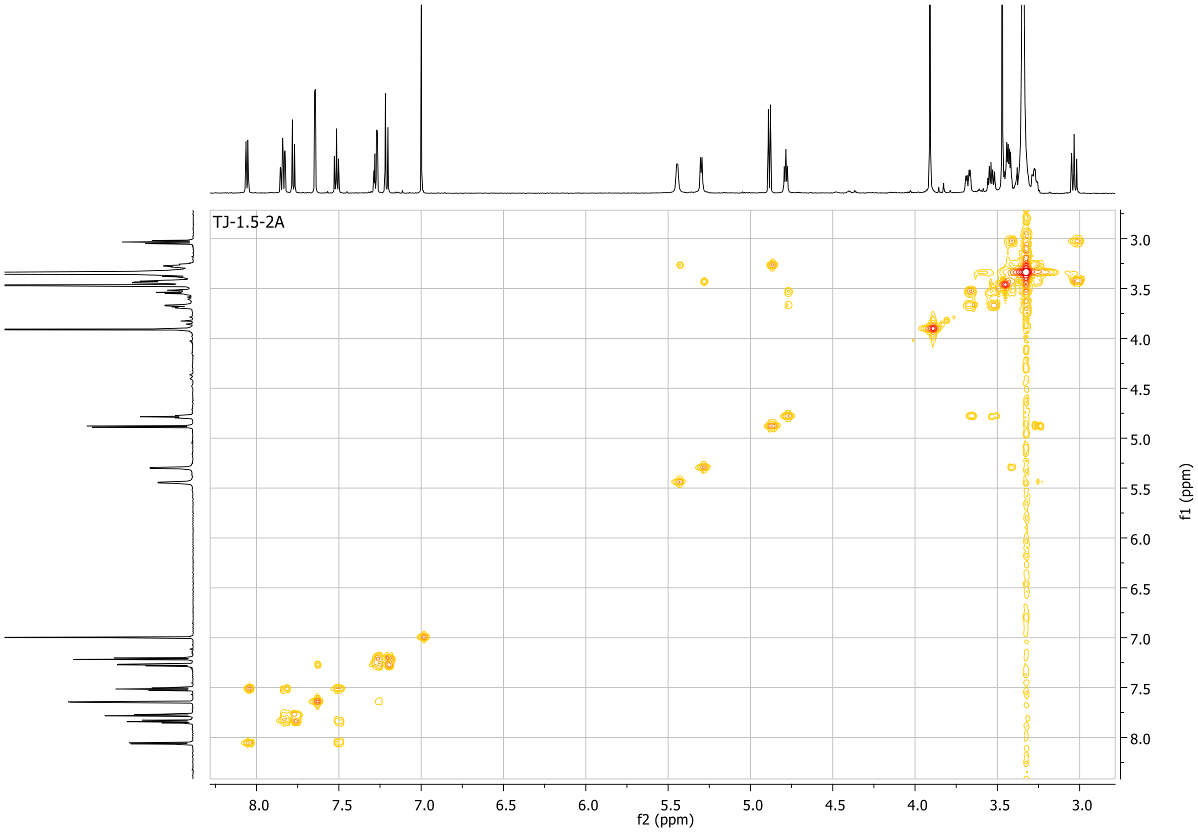


Fig.S15. HMQC spectral of 2'-methoxyflavone 5'-*O*-β-D-(4''-*O*-methyl)-glucopyranoside (**8**) (Acetone-$d_6$, 151 MHz)

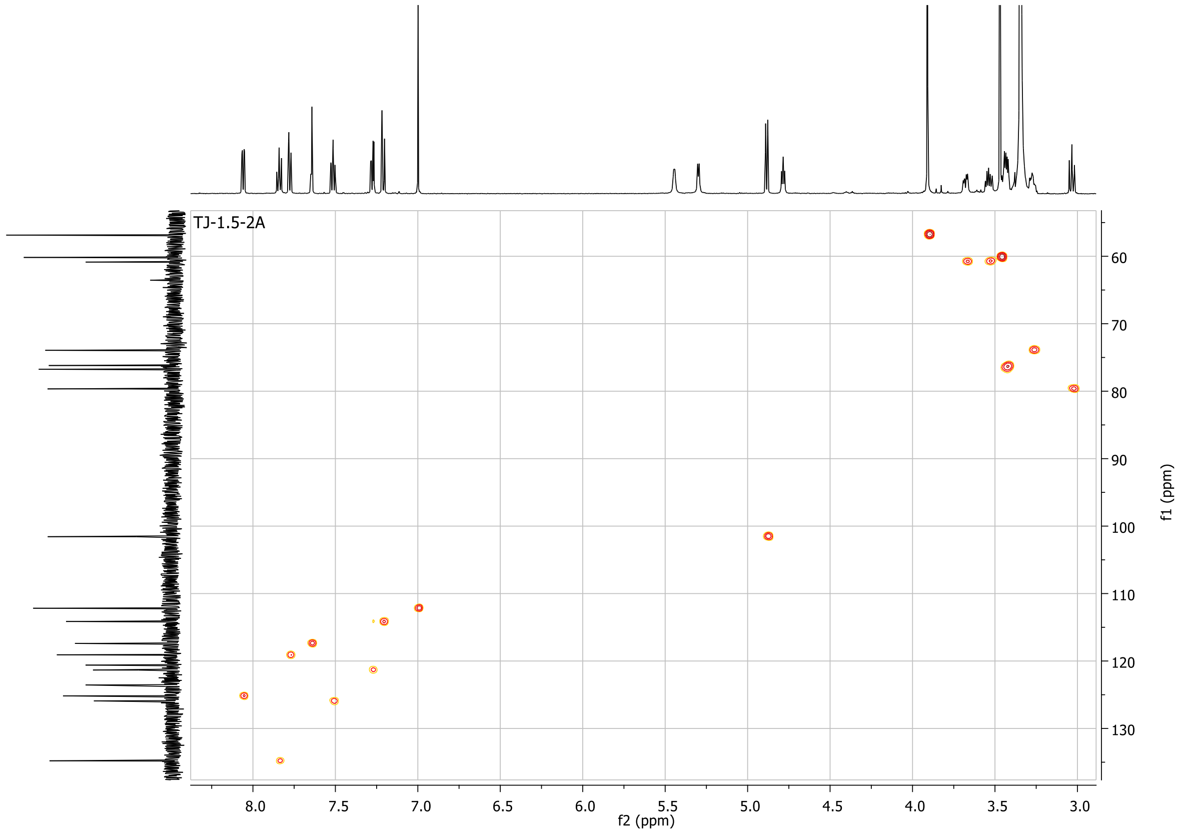

Fig.S16. HMBC spectral of 2’-methoxyflavone 5’-*O*-β-D-(4”-*O*-methyl)-glucopyranoside flavone (**8**) (Acetone-$d_6$, 151 MHz)

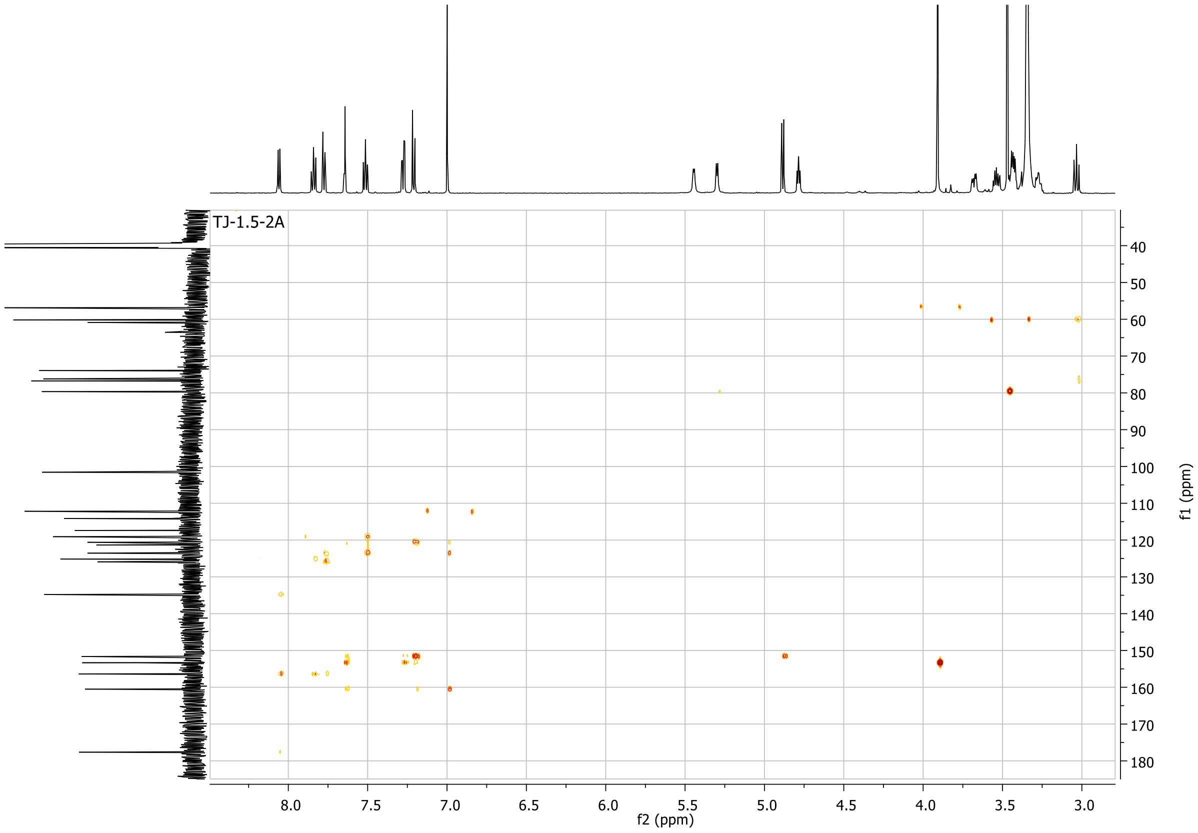

Fig.S17. MS analysis 2'-methoxyflavone 8-*O*-β-D-(4"-*O*-metyloglukopiranozylo)-2'-metoksyflawon (**7**)

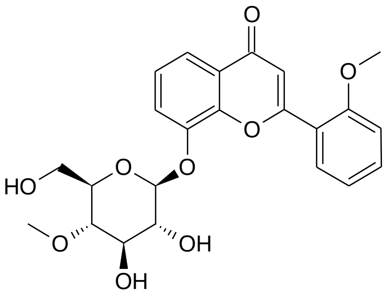


Molecular Formula = $C_{23}H_{24}O_9$
Formula Weight = 444.43126
Precursor =445.4000

CE: -15.0

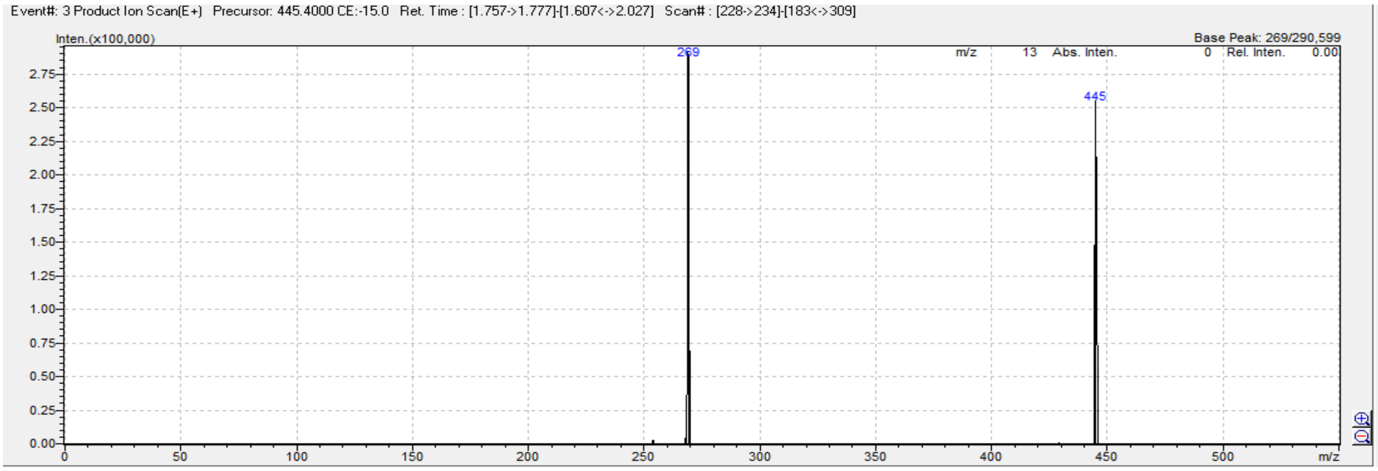


CE:-35.0

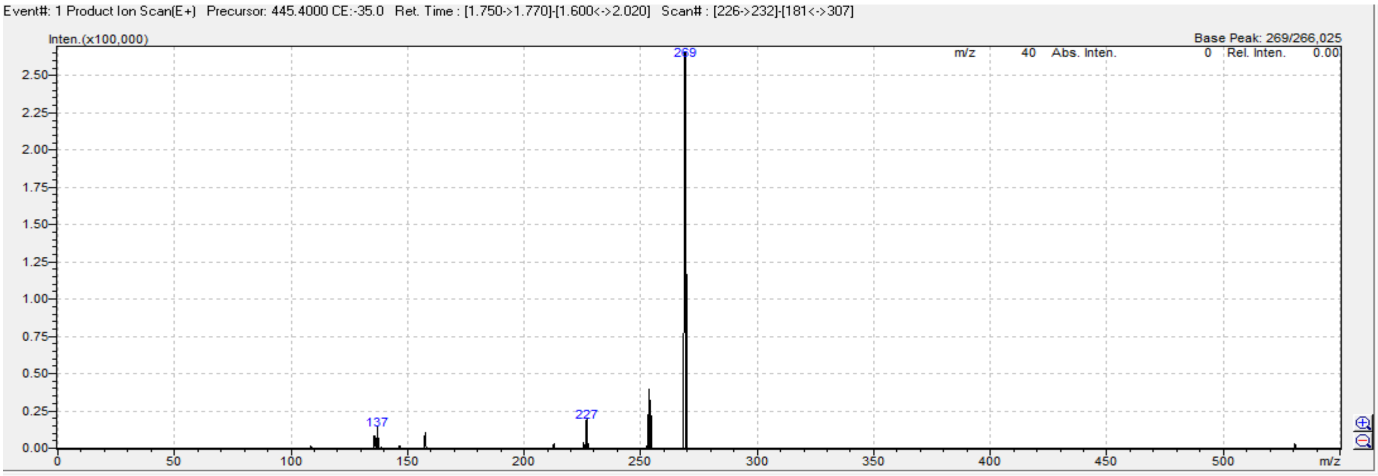


CE:-45.0

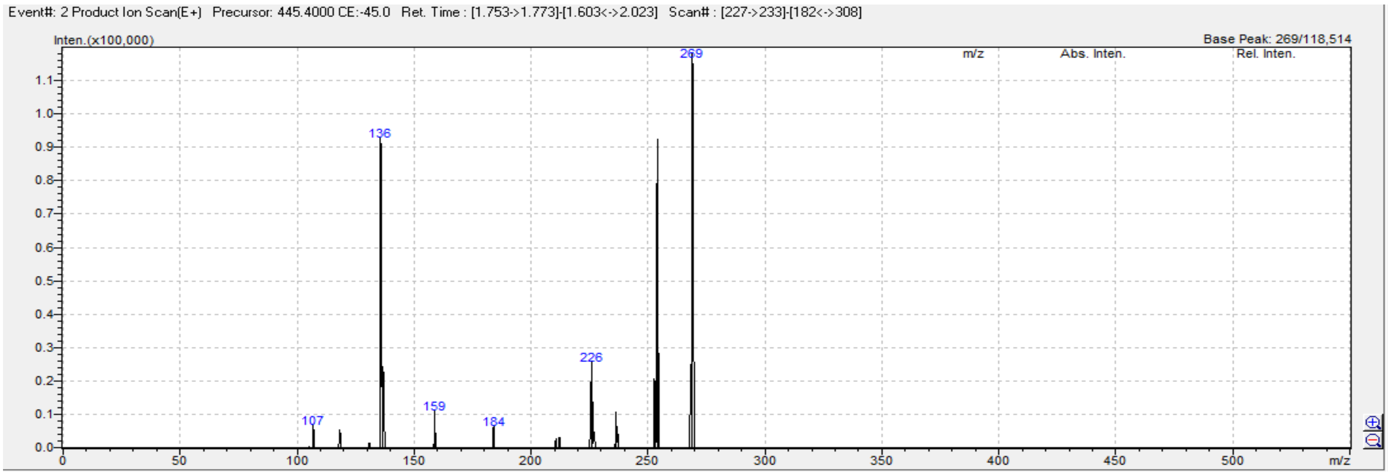

Fig.S18. $^{1}$H NMR spectral of 2’-methoxyflavone 8-*O*-β-D-(4”-*O*-methyl)-glucopyranoside (**7**) (Acetone-$d_6$, 600 MHz)

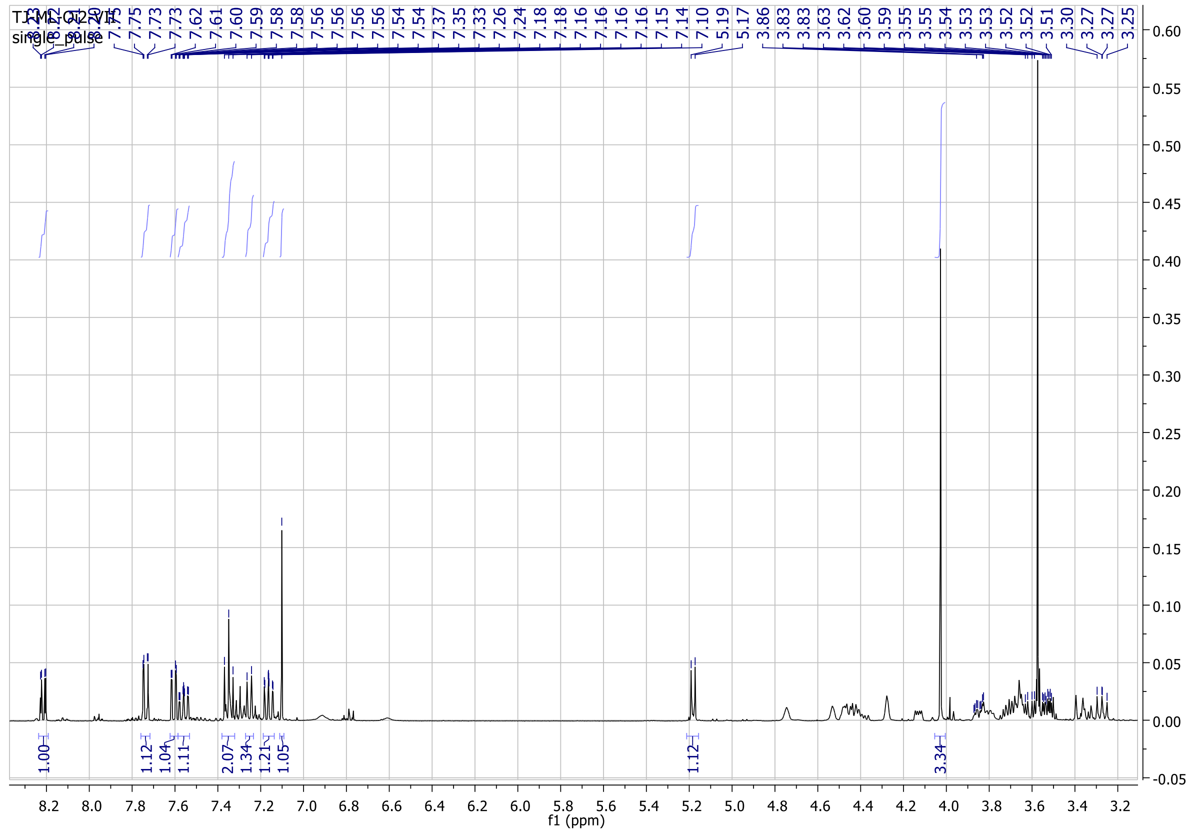

Fig.S19. Flavone part of the $^{1}$H NMR spectral 2’-methoxyflavone 8-*O*-β-D-(4”-*O*-methyl)-glucopyranoside (**7**) (Acetone-$d_6$, 600 MHz)

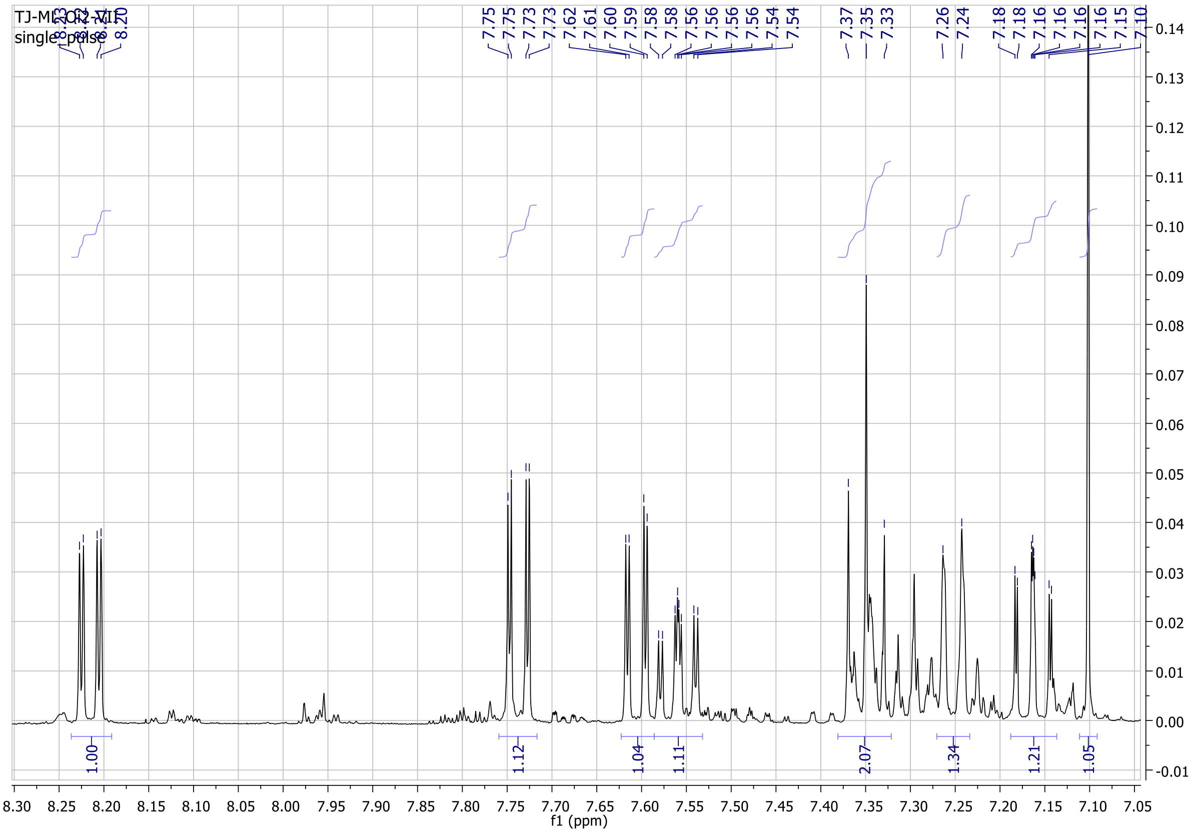

Fig.S20. $^{13}$C NMR spectral of 2'-methoxyflavone 8-*O*-β-D-(4"-*O*-methyl)-glucopyranoside (**7**) (Acetone-$d_6$, 151 MHz)

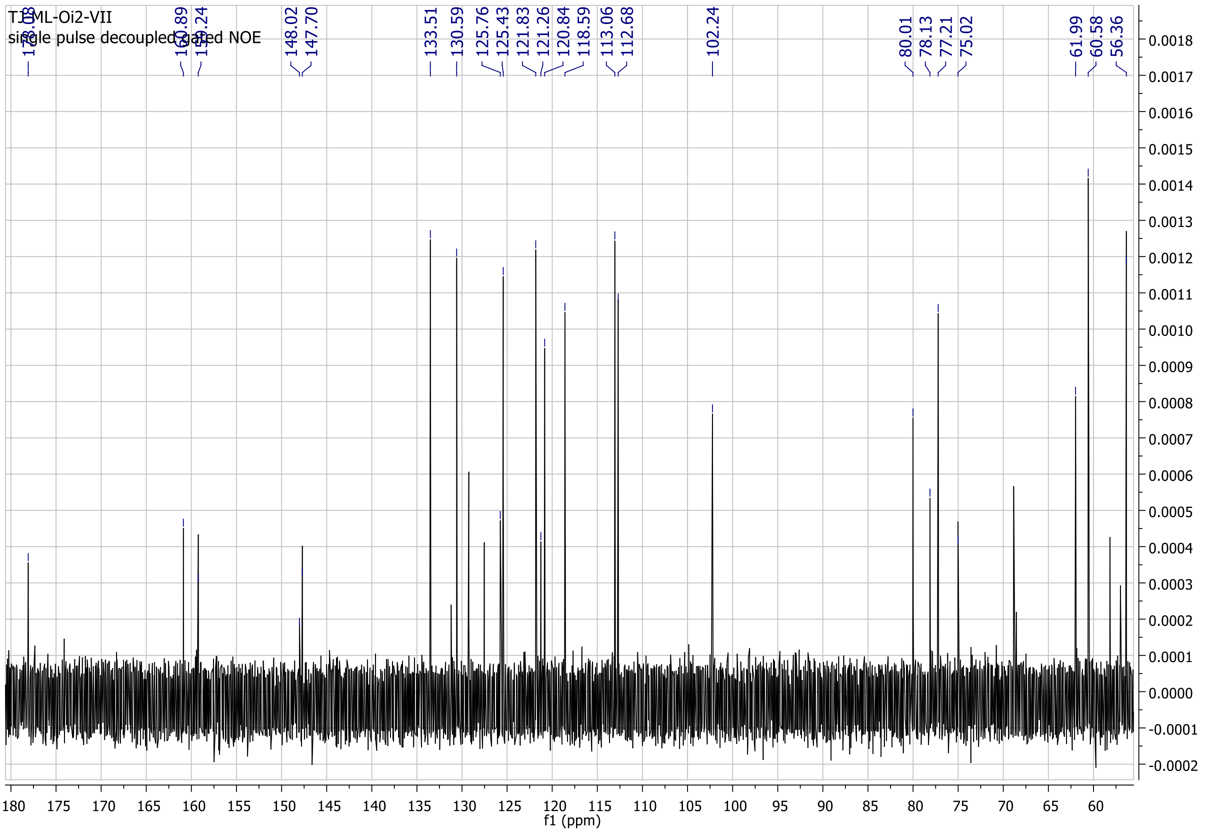


Fig.S21. COSY spectral of 2'-methoxyflavone 8-*O*-β-D-(4"-*O*-methyl)-glucopyranoside (**1**) (Acetone-$d_6$, 151 MHz)

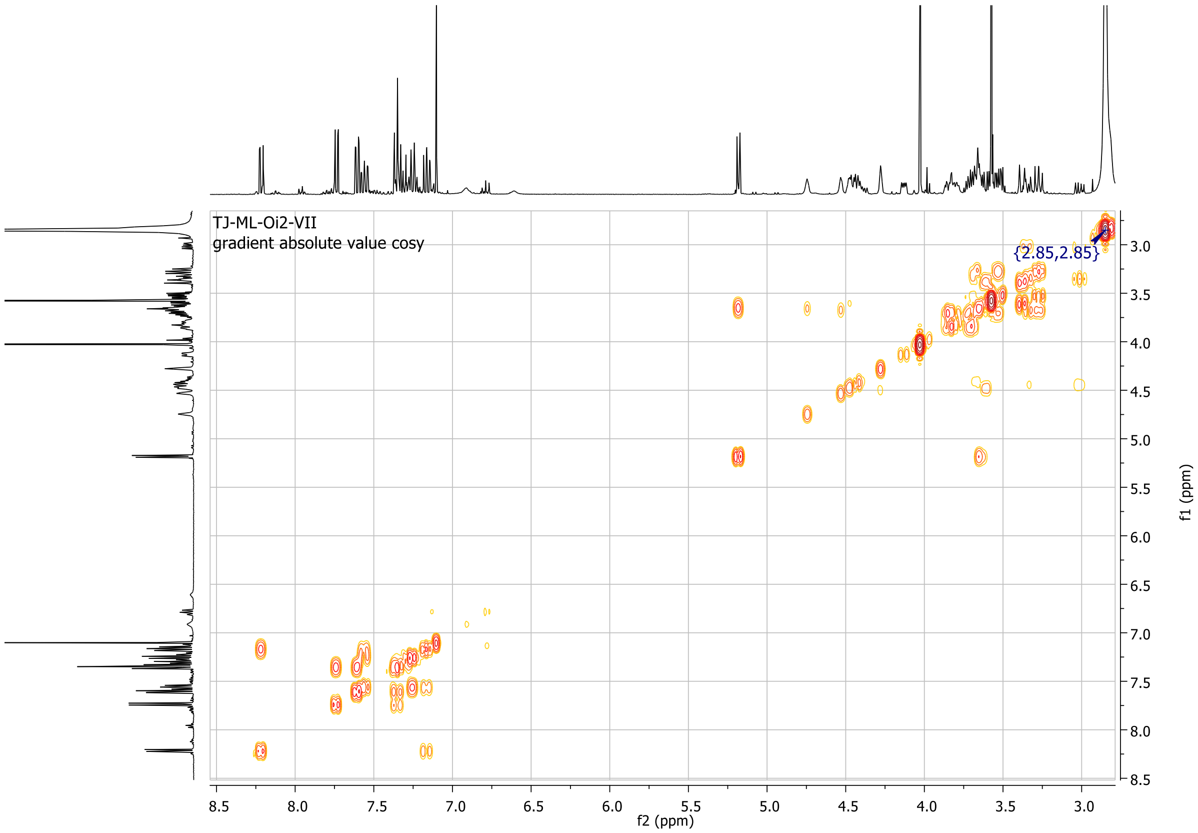

Fig.S22. HMQC spectral of 2’-methoxyflavone 8-*O*-β-D-(4”-*O*-methyl)-glucopyranoside (**7**) (Acetone-$d_6$, 151 MHz)

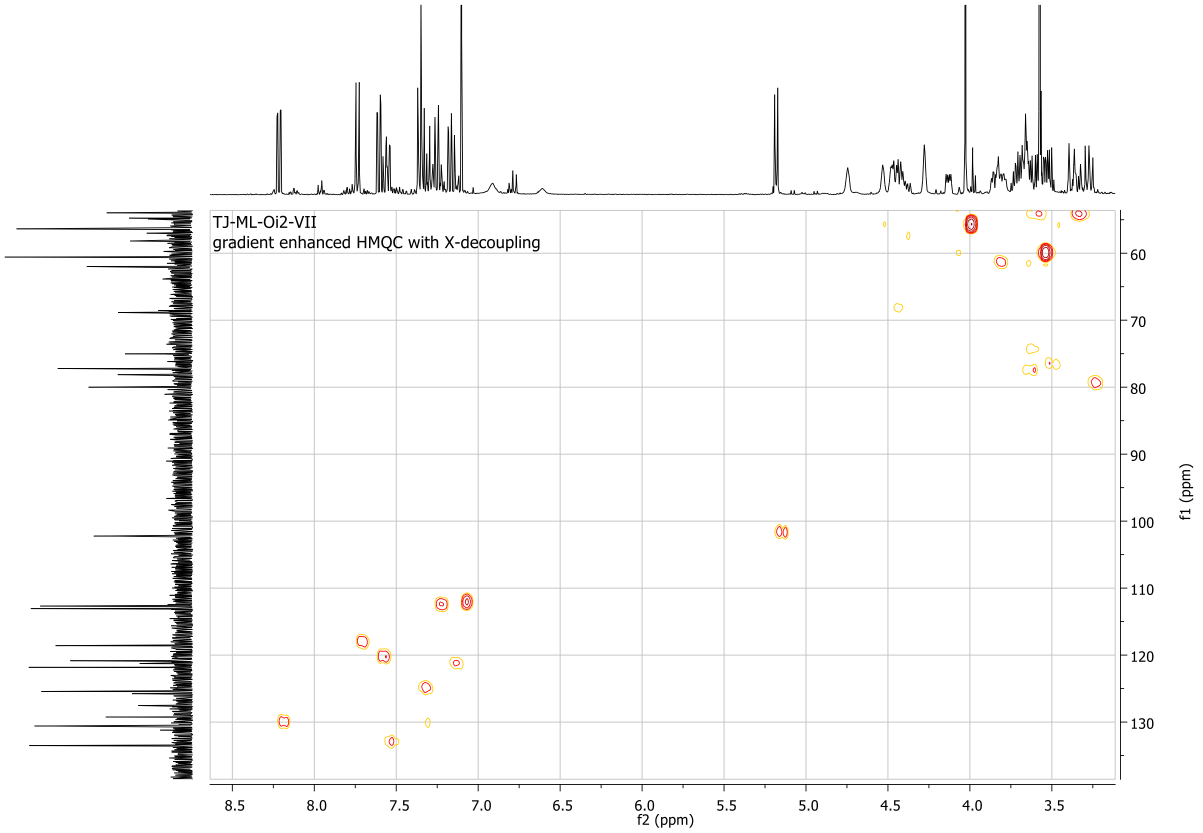


Fig.S23. HMBC spectral of 2’-methoxyflavone 8-*O*-β-D-(4”-*O*-methyl)-glucopyranoside flavone (**7**) (Acetone-$d_6$, 151 MHz)

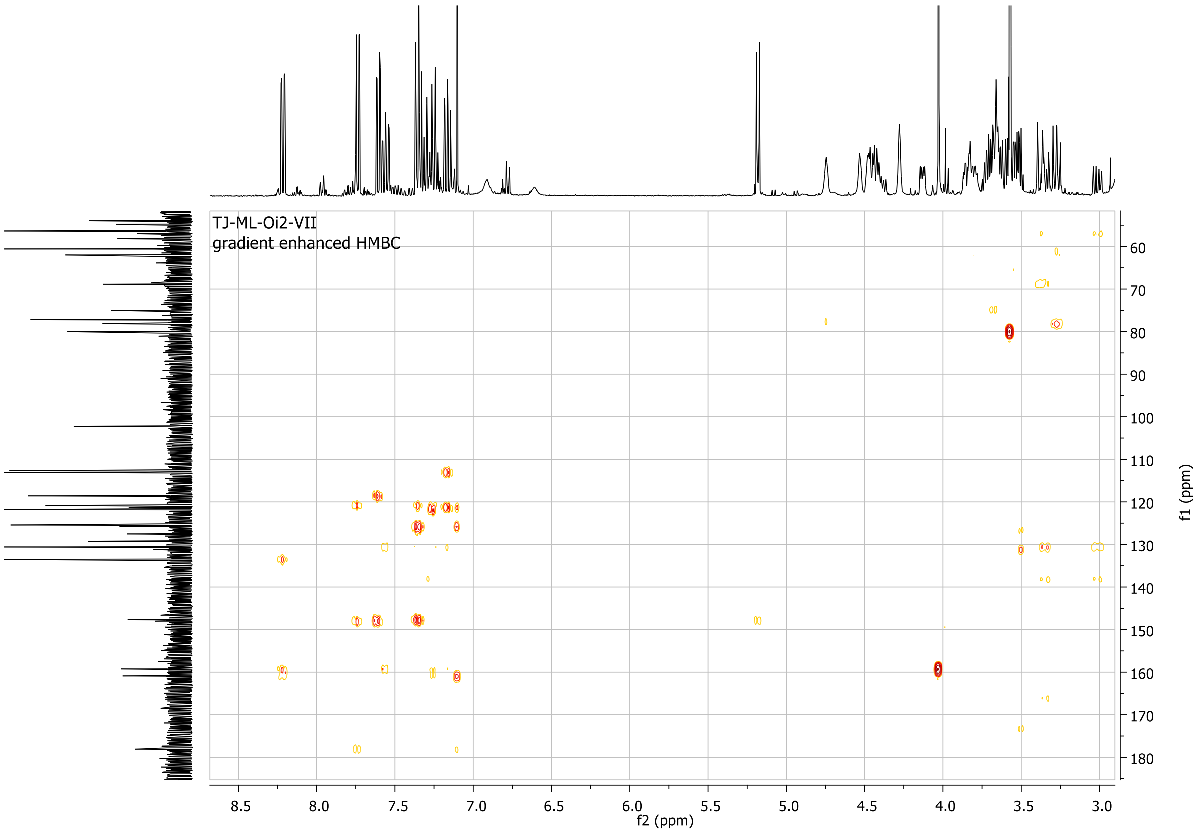

Fig.S24. MS analysis 3-*O*-β-D-(4”-*O*-metyloglukopiranozylo)-2’-metoksyflawon (**9**)

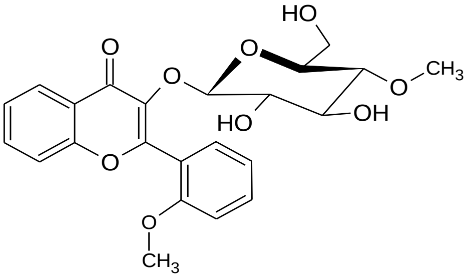


| | |
|---|---|
| Molecular Formula | = $C_{23}H_{24}O_9$ |
| Formula Weight | = 444.43126 |
| Precursor | =445.4000 |

CE: -15.0

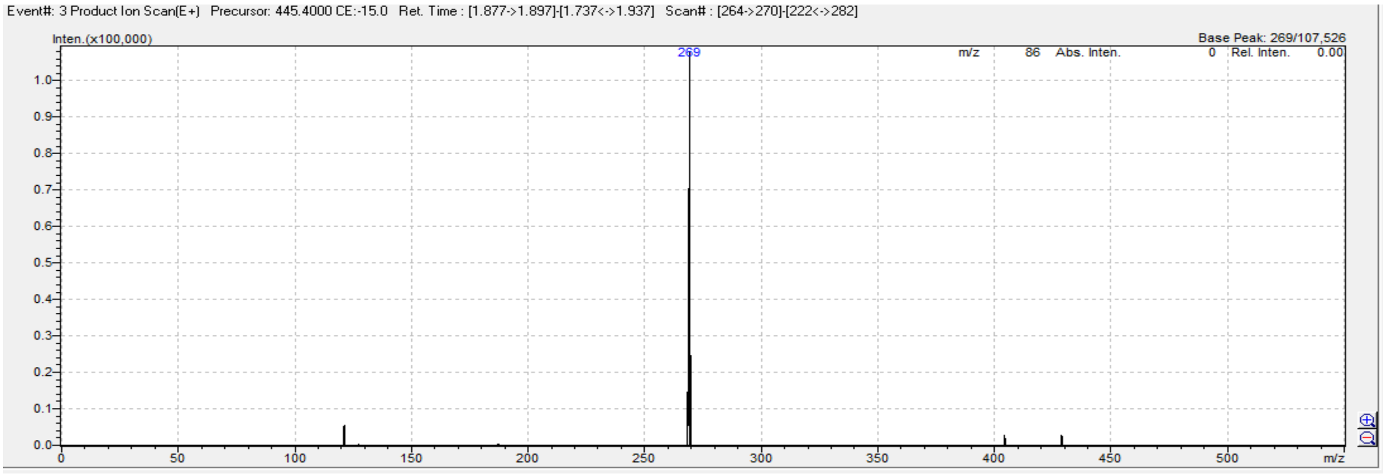


CE:-35.0

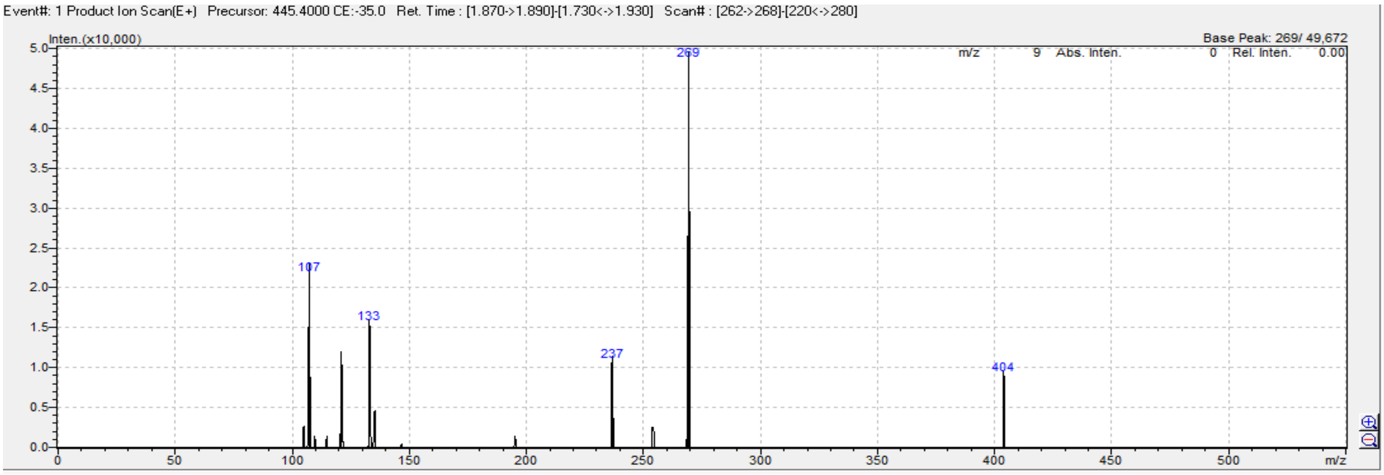


CE:-45.0

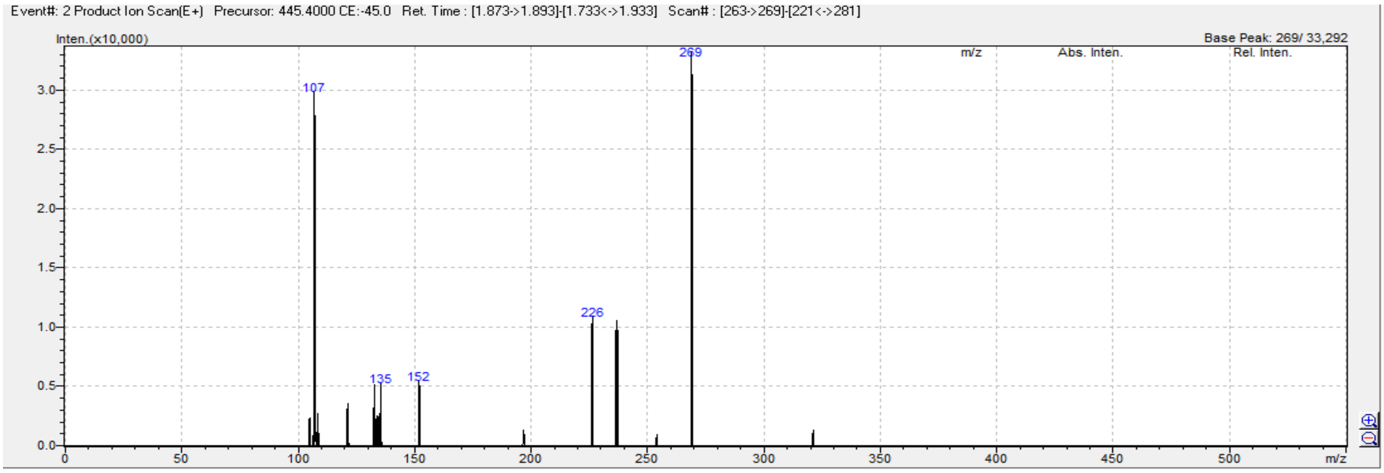

Fig.S25. $^{1}$H NMR spectral of 2'-methoxyflavone 3-*O*-β-D-(4''-*O*-methyl)-glucopyranoside (**9**) (DMSO-$d_6$, 600 MHz)

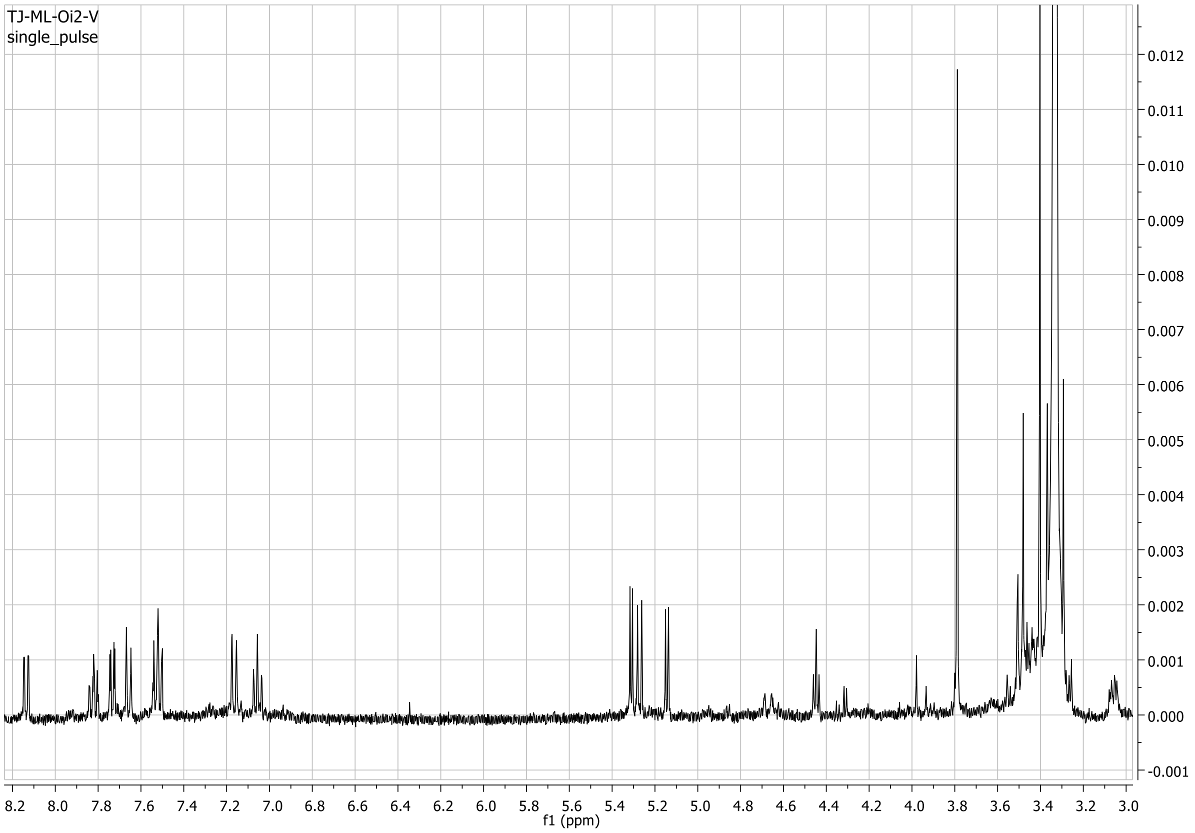


Fig.S26. Flavone part of the $^{1}$H NMR spectral 2'-methoxyflavone 3-*O*-β-D-(4''-*O*-methyl)-glucopyranoside (**9**) (DMSO-$d_6$, 600 MHz)

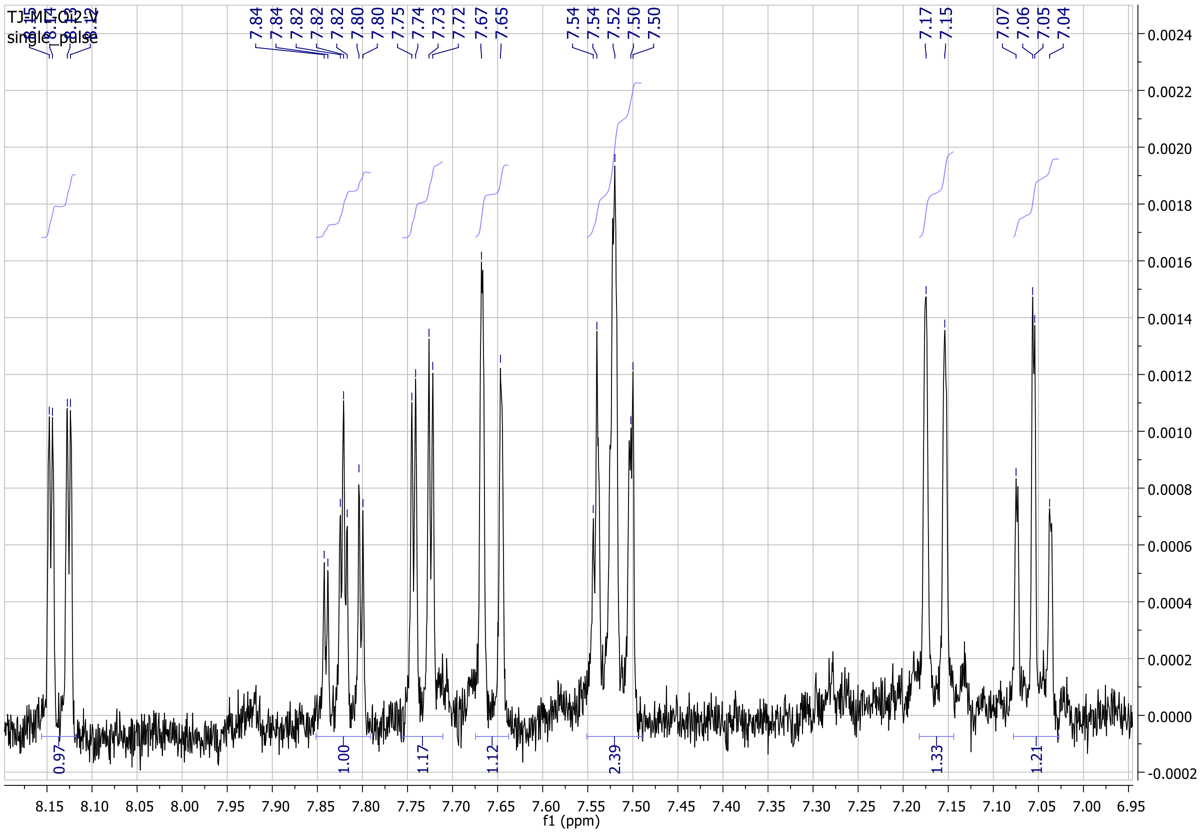

Fig.S27. Glucopyranoside part of the $^{1}$H NMR spectral 2’-methoxyflavone 3-*O*-β-D-(4”-*O*-methyl)-glucopyranoside (**9**) (DMSO-$d_6$, 600 MHz)

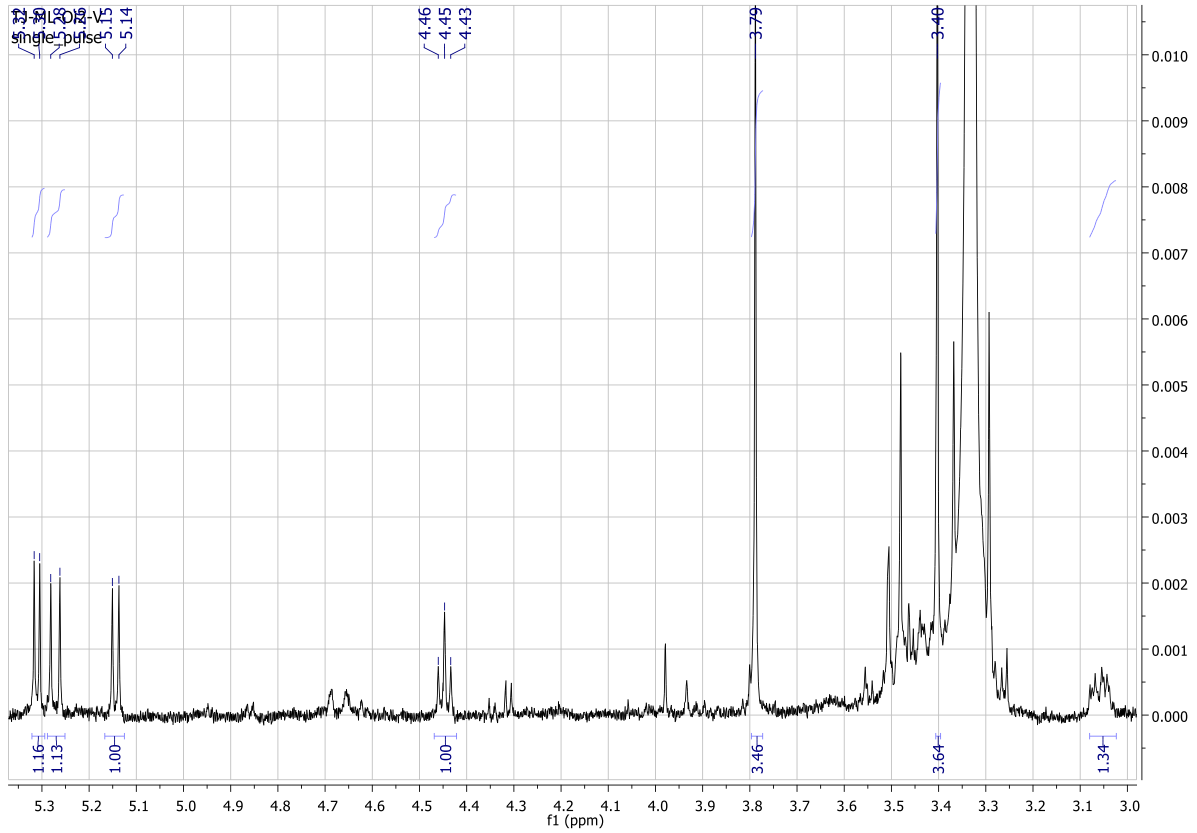

Fig.S28. MS analysis 2'-hydroxyflavone (**10**)

Molecular Formula: $C_{15}H_{10}O_3$
Formula Weight: 238.2381
Precursor: 239.2000

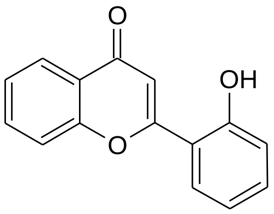


CE: -15.0

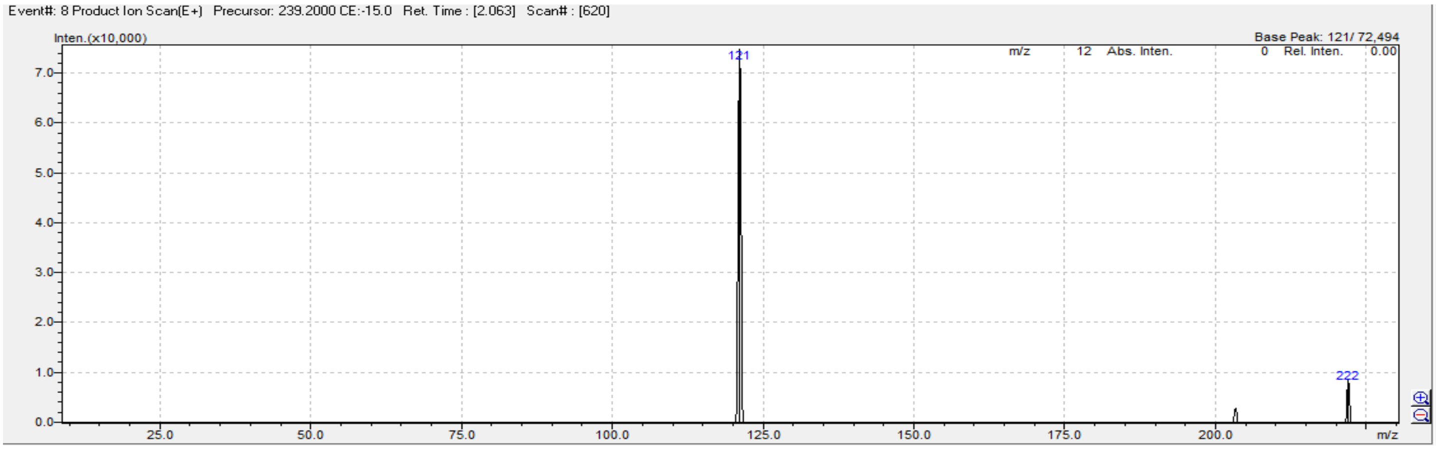


CE:-35.0

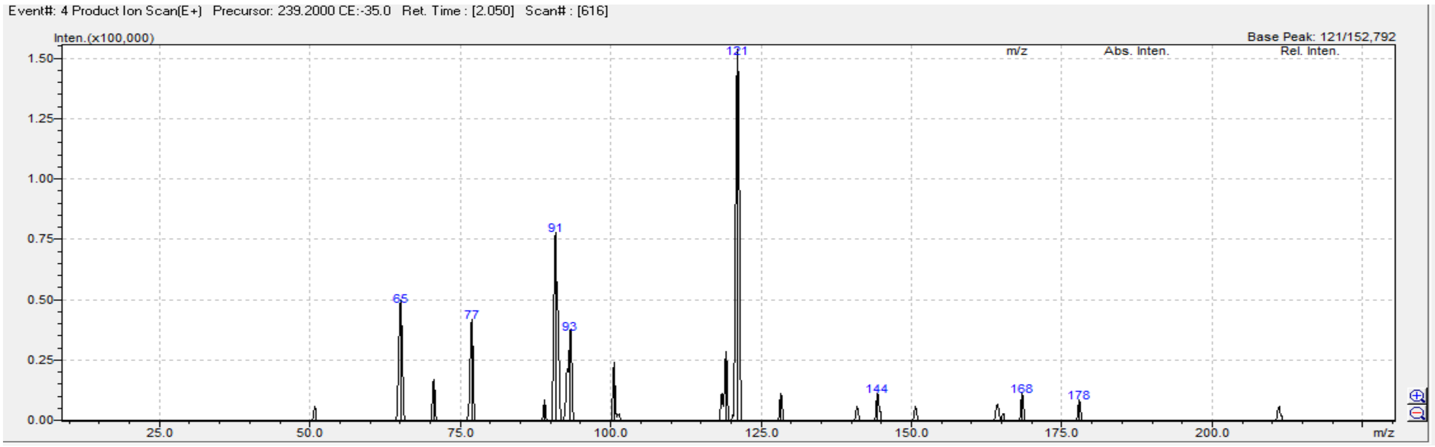


CE:-45.0

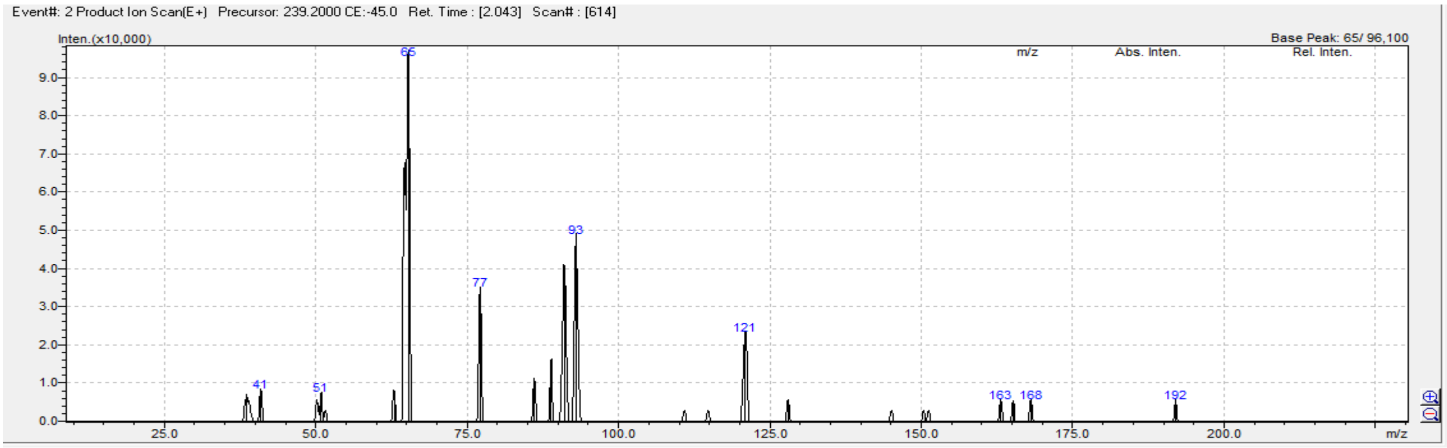

Fig.S29. $^{1}$H NMR spectral of 2’-hydroxyflavone (**10**) (Acetone-$d_6$, 600 MHz)

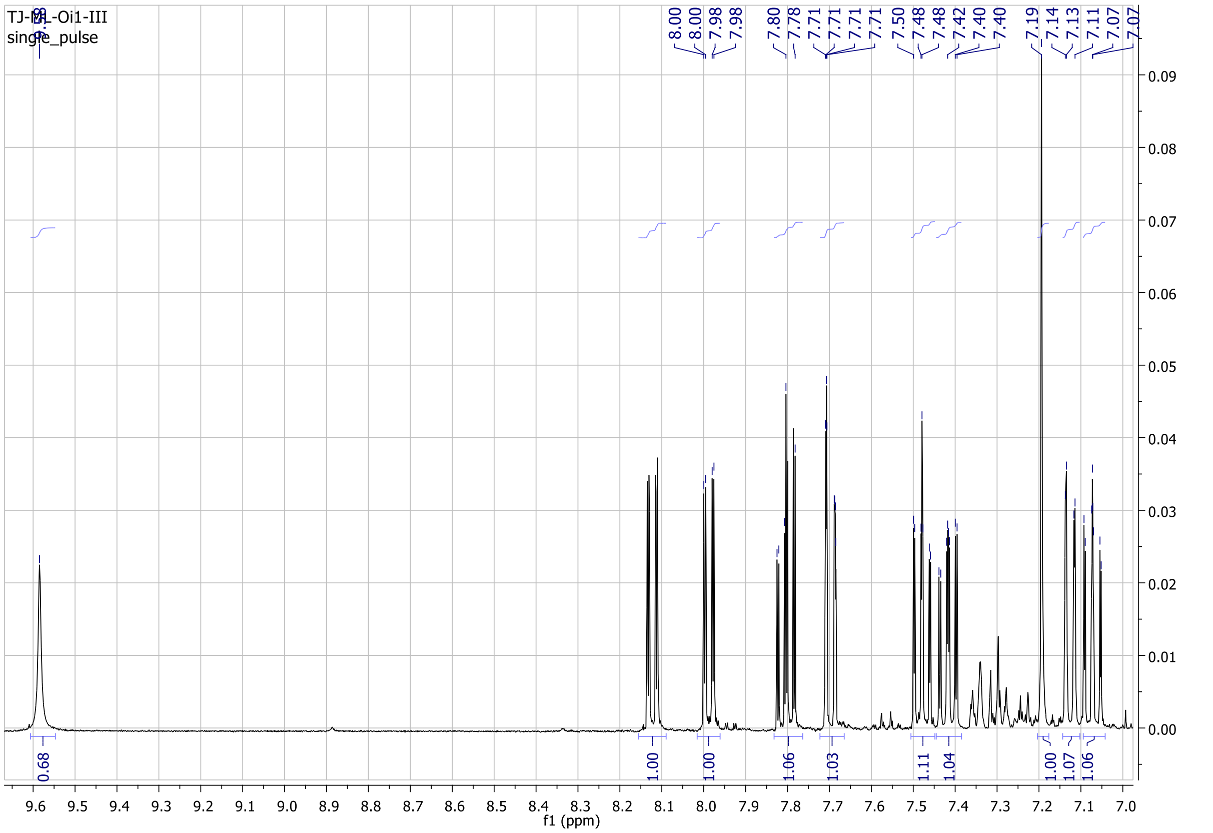


Fig.S30. $^{13}$C NMR spectral of 2’-hydroxyflavone (**10**) (Acetone-$d_6$, 151 MHz)

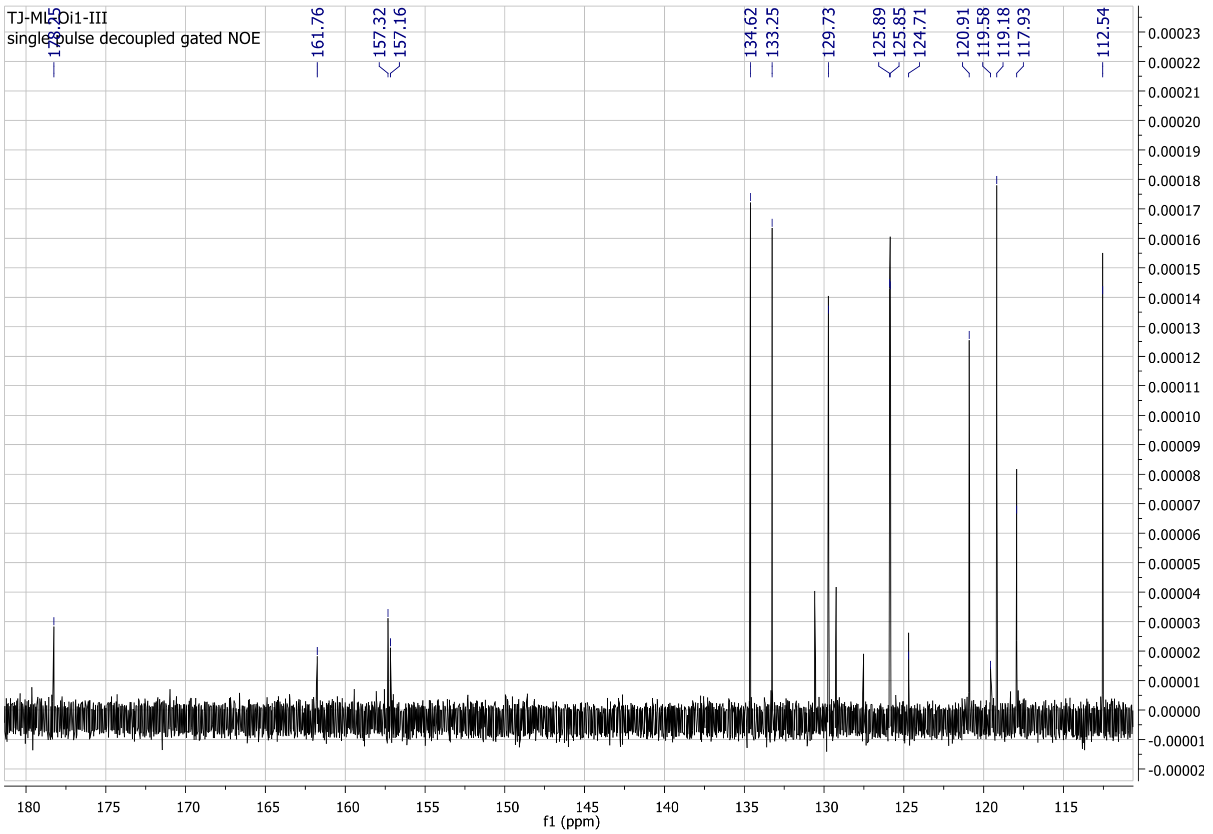

Fig.S31. COSY spectral of 2’-hydroxyflavone (**10**) (Acetone-$d_6$, 151 MHz)

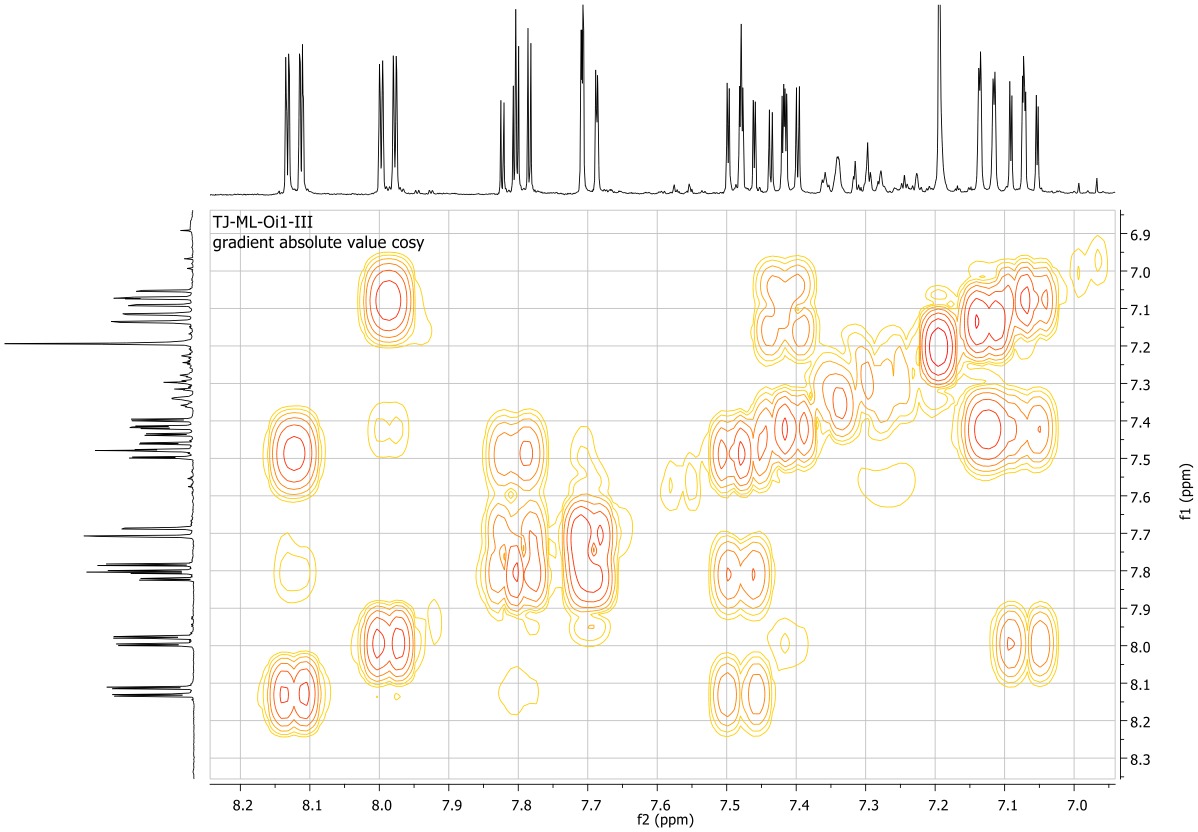


Fig.S32. HMQC spectral of 2’-hydroxyflavone (**10**) (Acetone-$d_6$, 151 MHz)

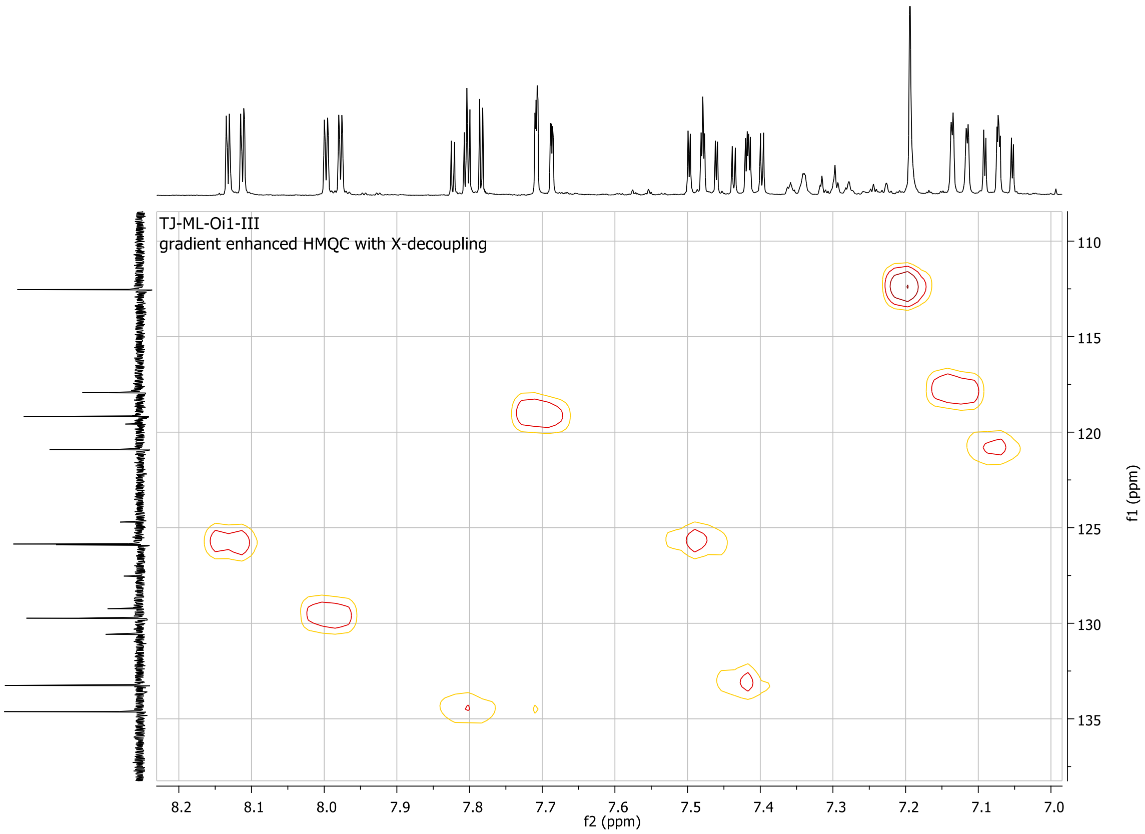

Fig.S33. HMBC spectral of 2'-hydroxyflavone (**10**) (Acetone-$d_6$, 151 MHz)

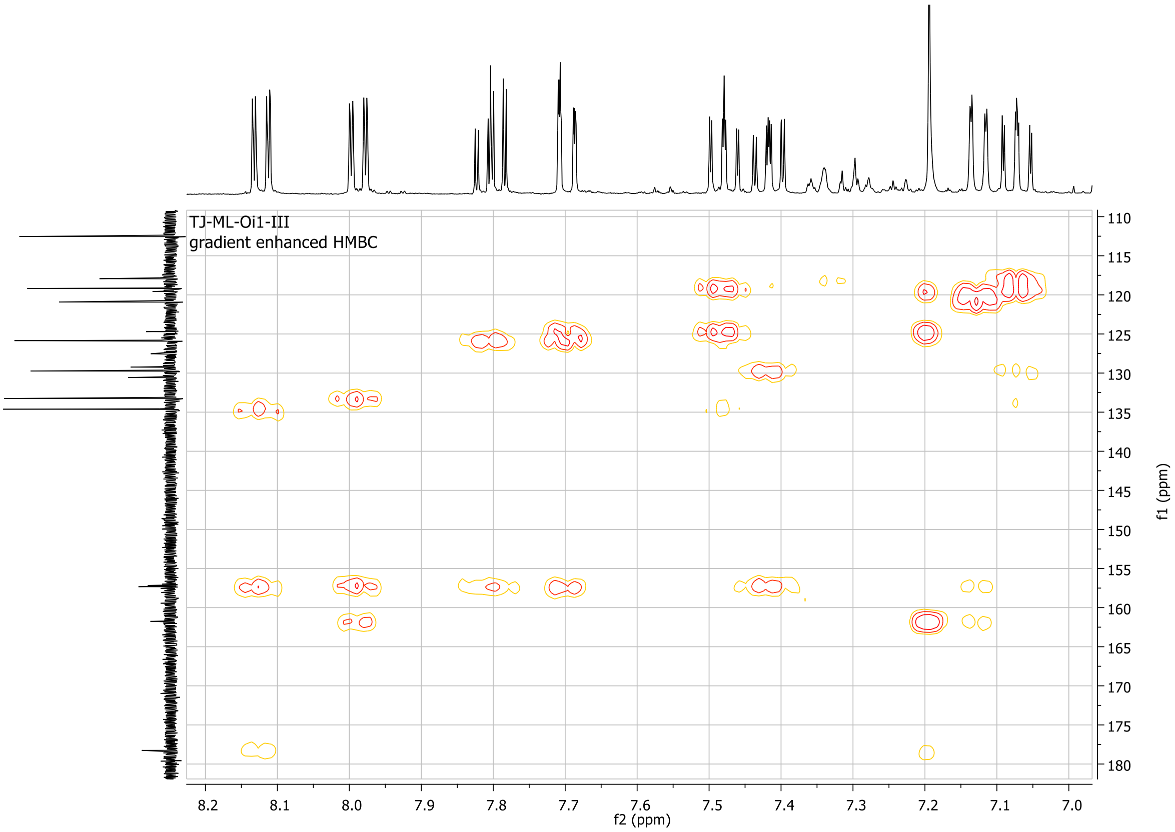

Fig.S34. MS analysis 3’-hydroxyflavone (**11**)

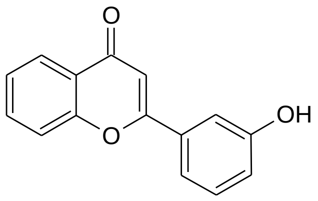


Molecular Formula: $C_{15}H_{10}O_3$

Formula Weight: 238.2381

Precursor: 239.2000

CE: -15.0

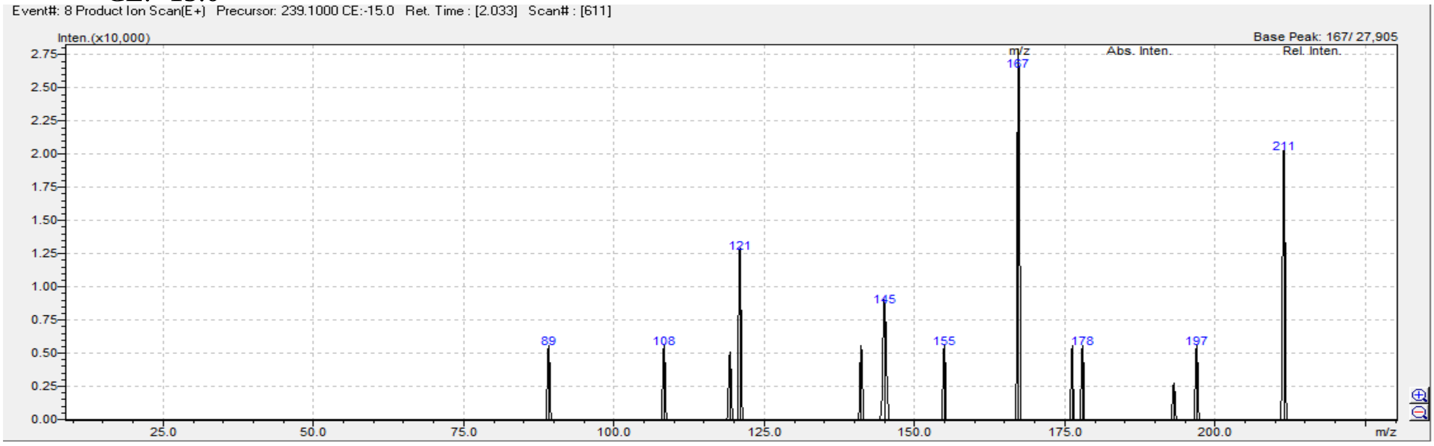


CE:-35.0

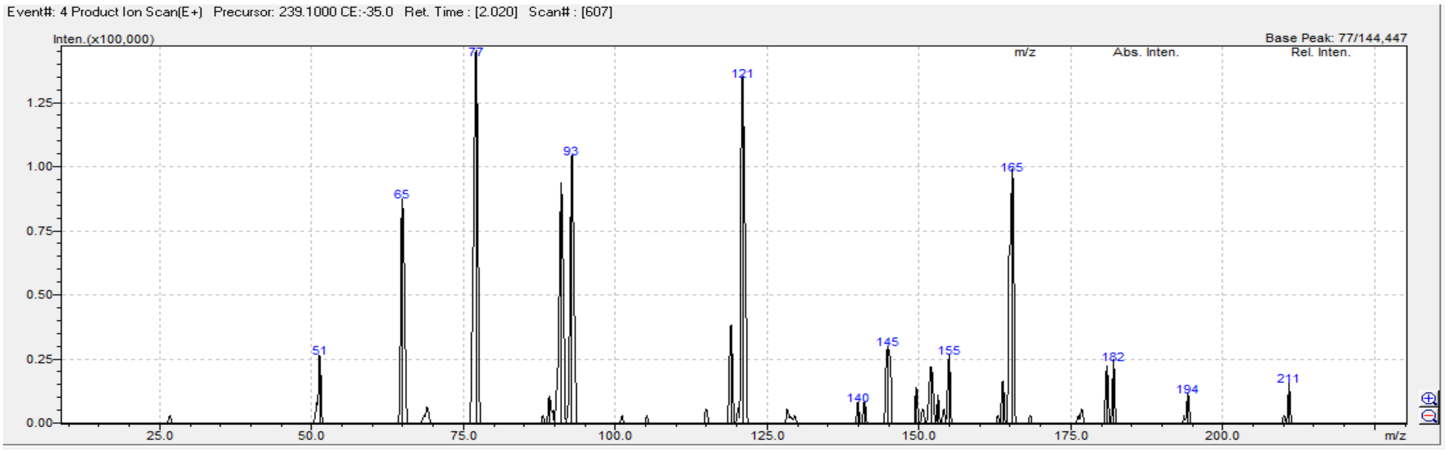


CE:-45.0

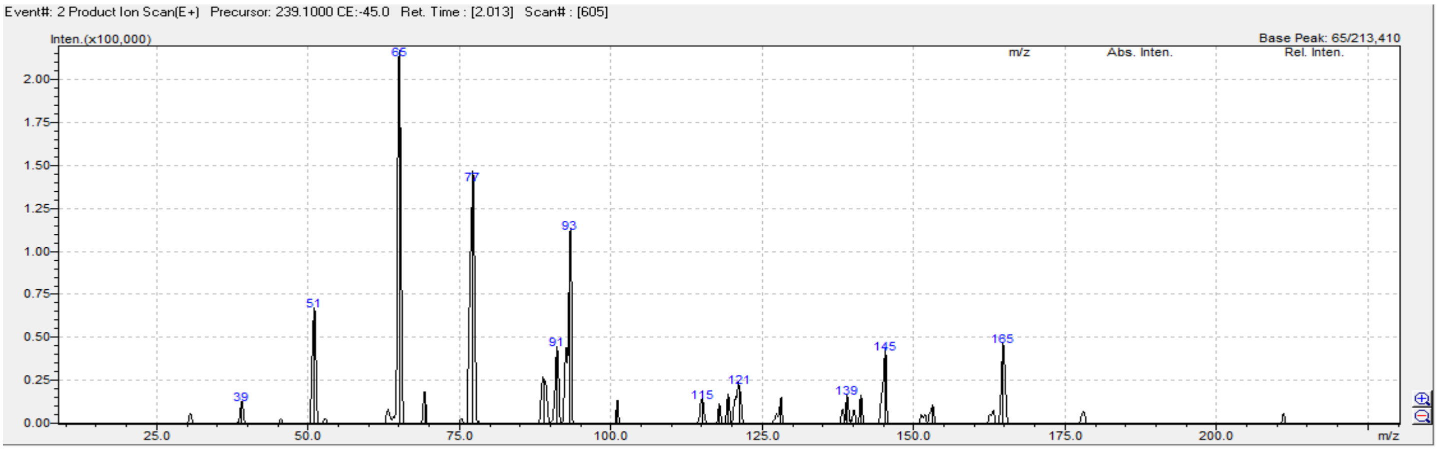

Fig.S35. $^{1}$H NMR spectral of 3’-hydroxyflavone (**11**) (DMSO-$d_6$, 600 MHz)

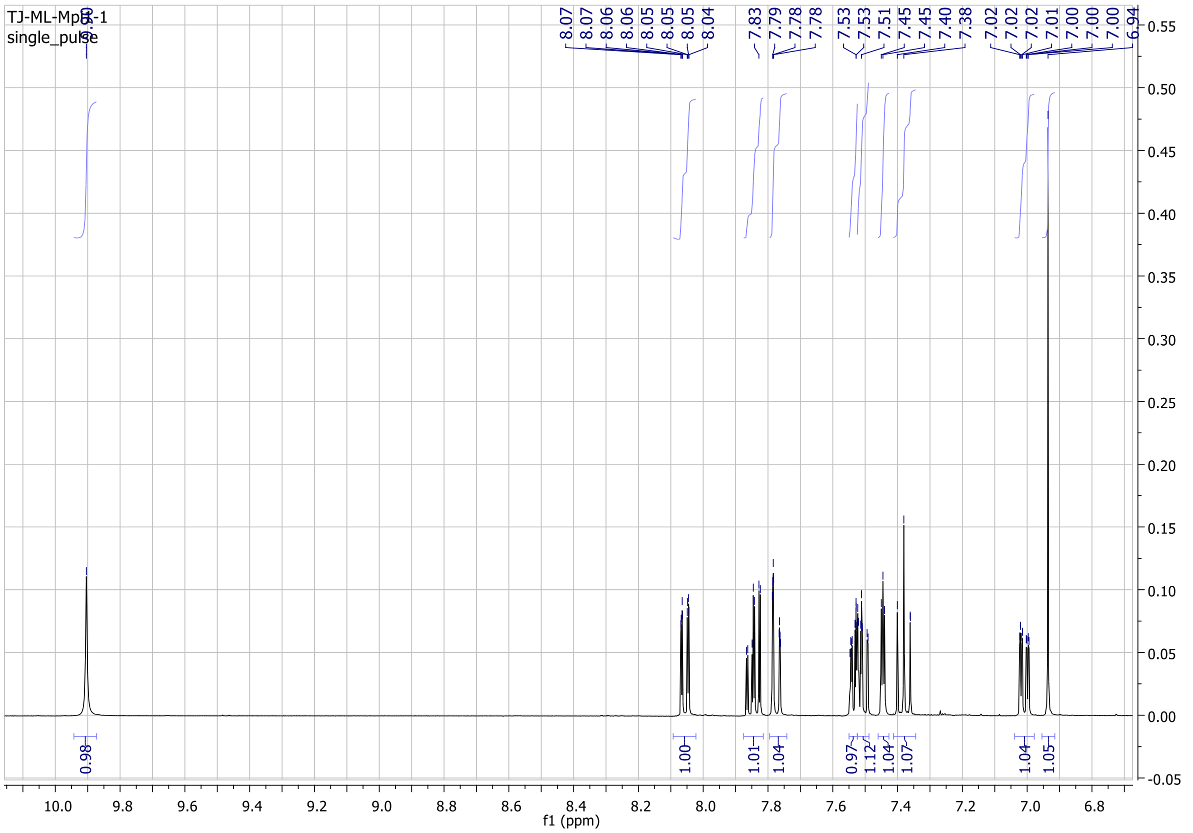

Fig.S36. $^{13}$C NMR spectral of 3’-hydroxyflavone (**11**) (DMSO-$d_6$, 151 MHz)

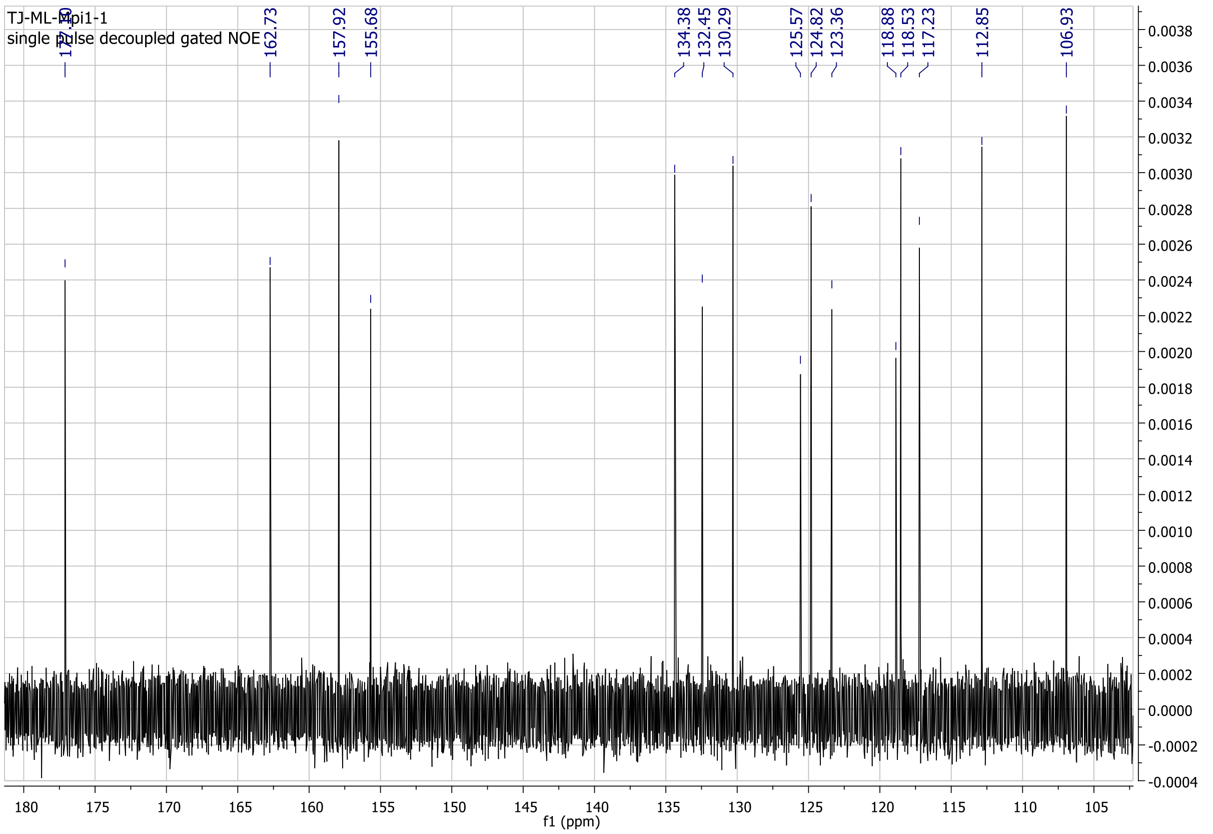

Fig.S37. COSY spectral of 3’-hydroxyflavone (**11**) (DMSO-$d_6$, 151 MHz)

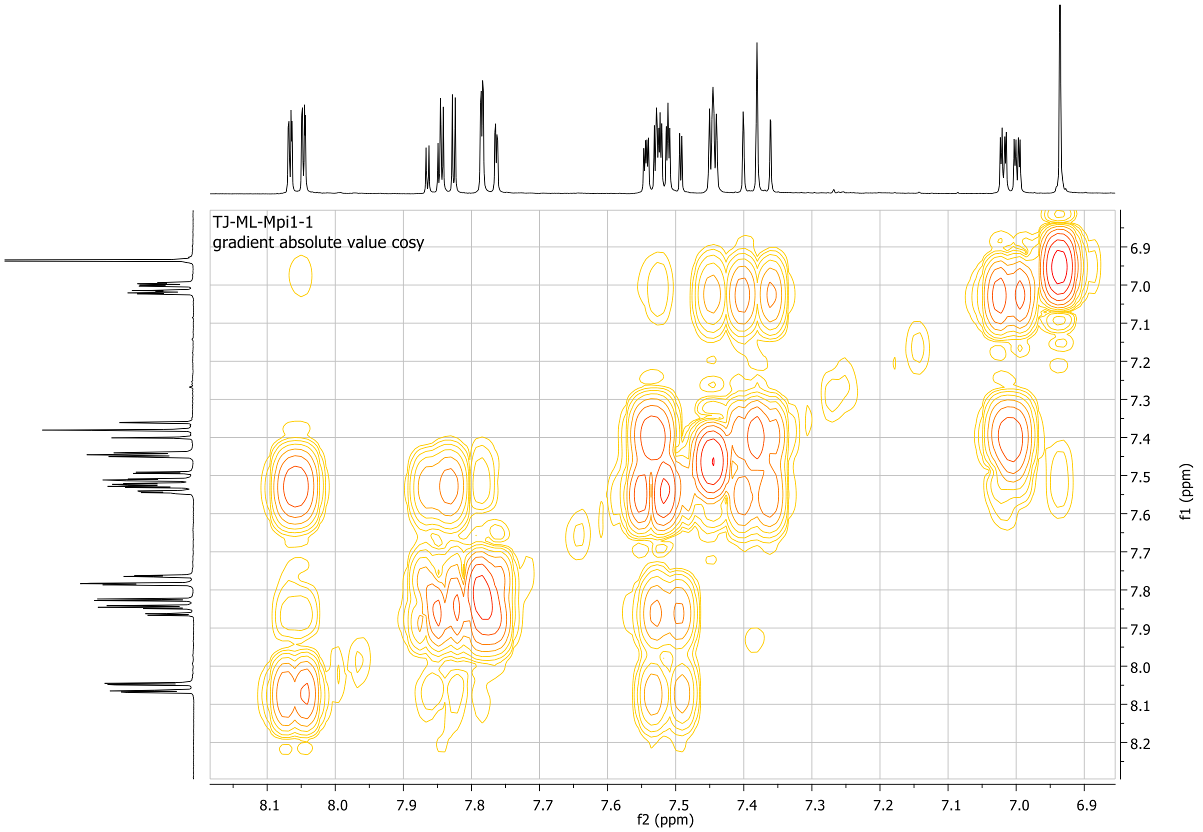


Fig.S38. HMQC spectral of 3’-hydroxyflavone (**11**) (DMSO-$d_6$, 151 MHz)

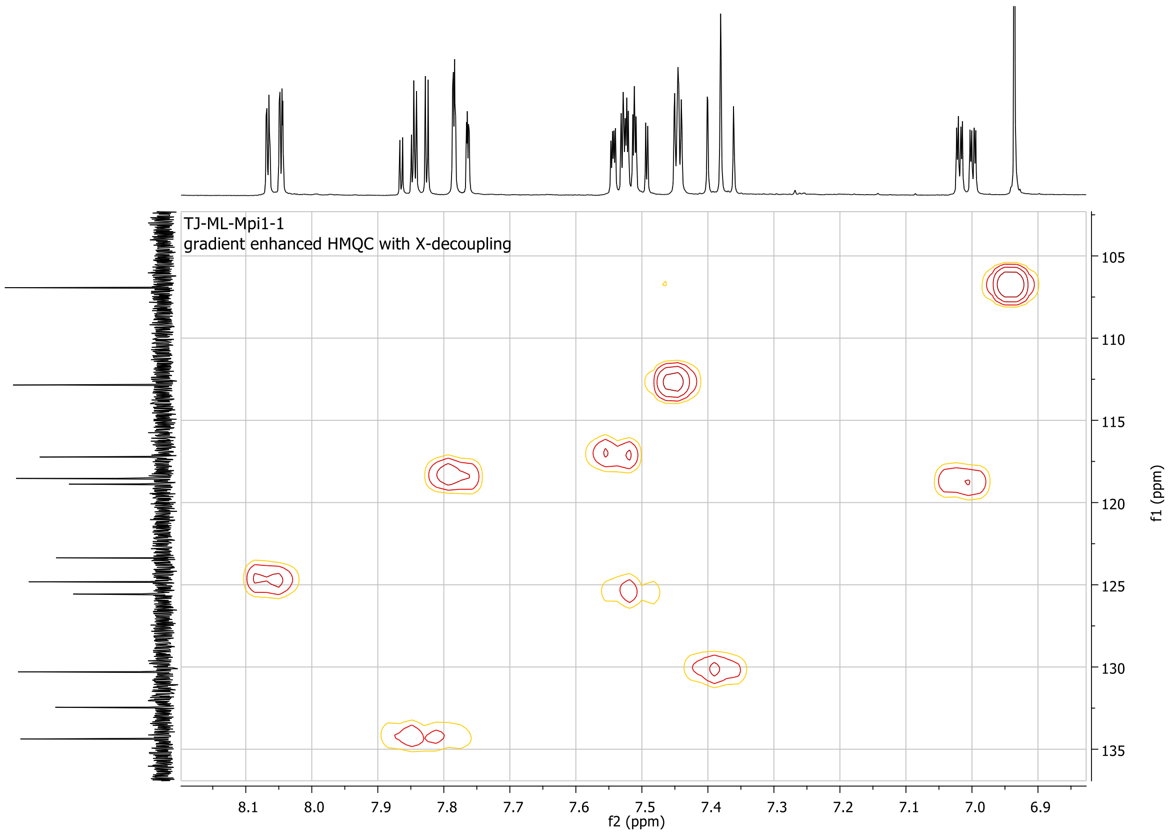

Fig.S39. HMBC spectral of 3’-hydroxyflavone (**11**) (DMSO-$d_6$, 151 MHz)

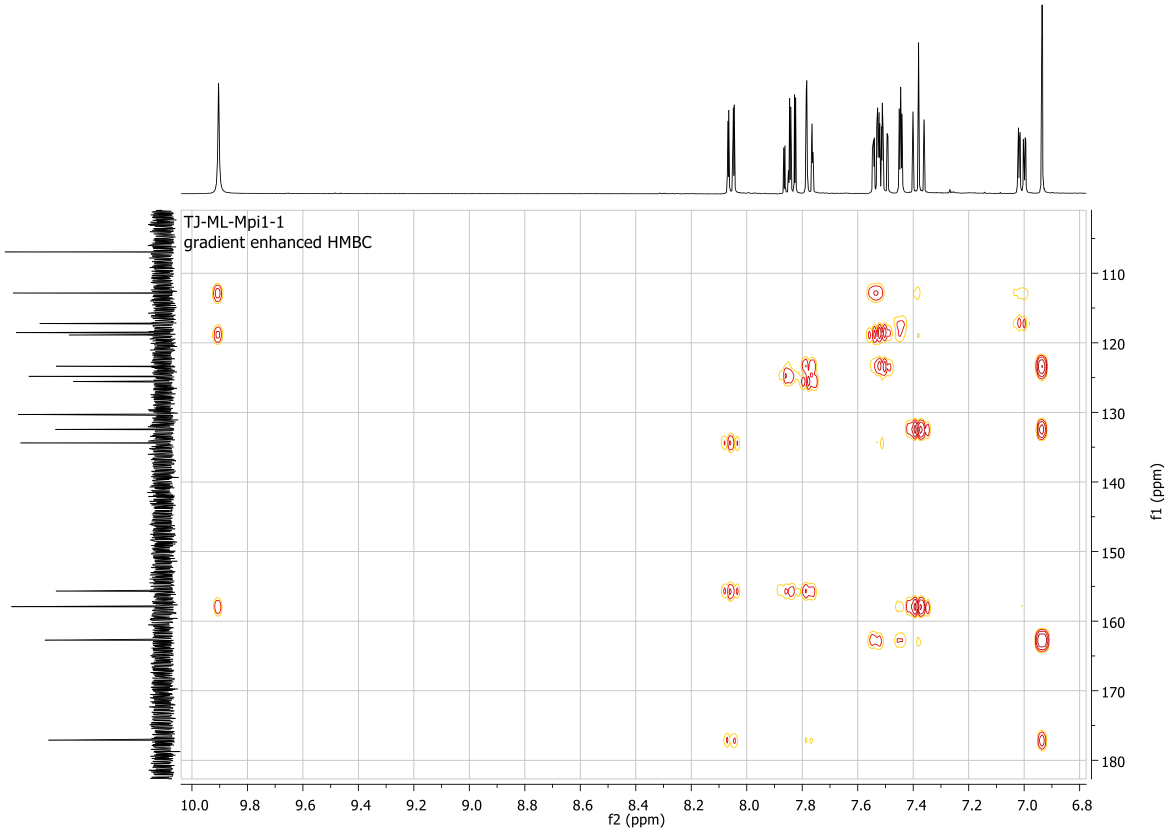

Fig.S40. MS analysis flavone 3’-*O*-β-D-(4”-*O*-methyl)-glucopyranoside (**12**)

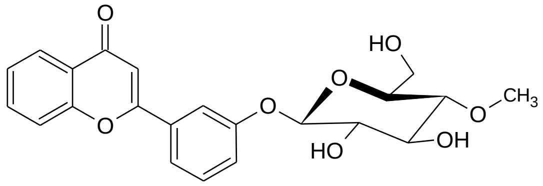


Molecular Formula = $C_{22}H_{22}O_8$
Formula Weight = 414.40528
Precursor =415.4000

CE: -15.0

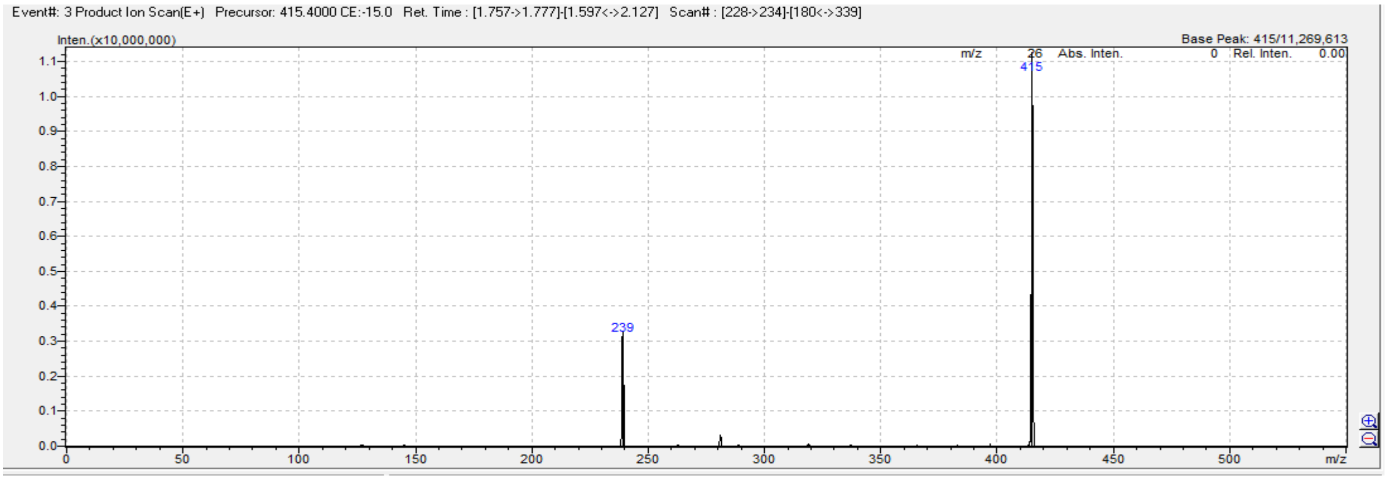


CE:-35.0

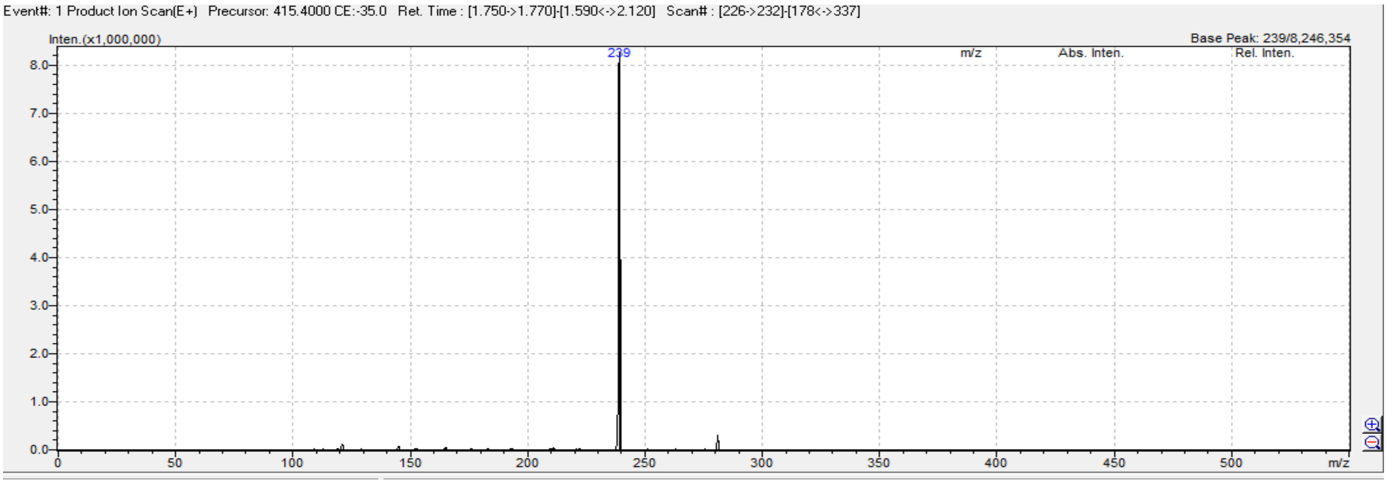


CE:-45.0

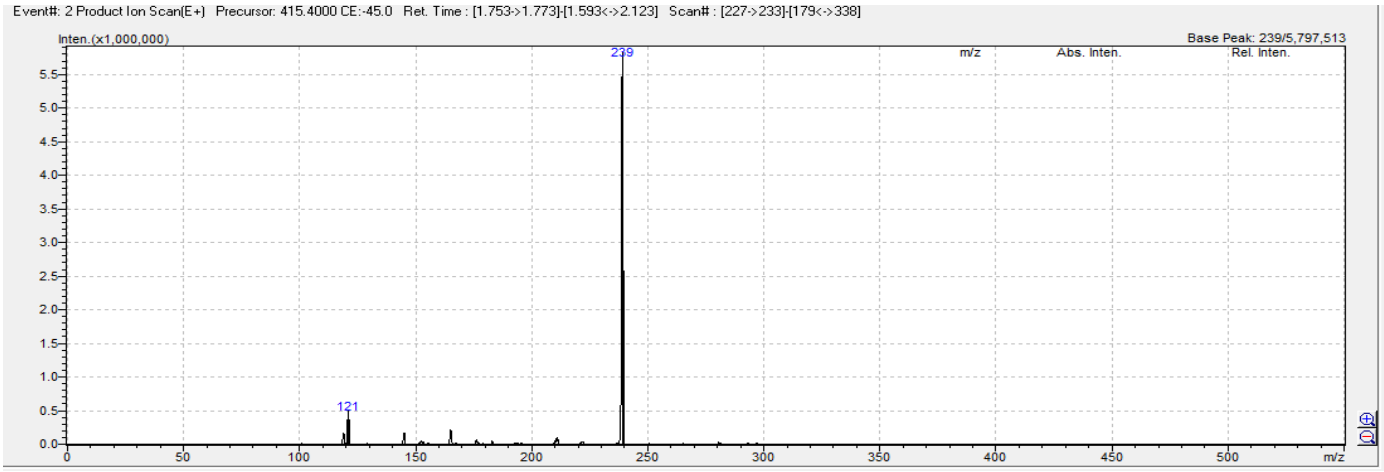

Fig.S41. $^{1}$H NMR spectral of flavone 3'-*O*-β-D-(4"-*O*-methyl)-glucopyranoside (**12**) (DMSO-$d_6$, 600 MHz)

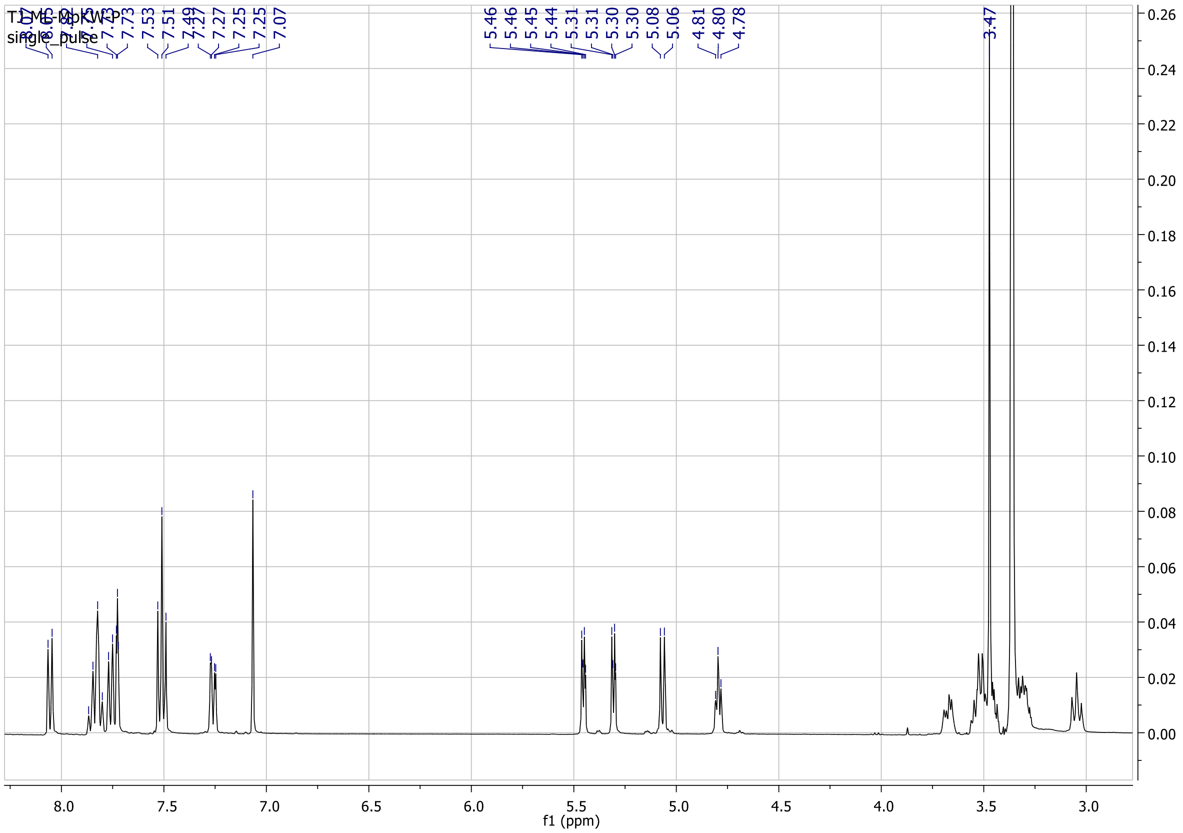


Fig.S42. Flavone part of the $^{1}$H NMR spectral flavone 3'-*O*-β-D-(4"-*O*-methyl)-glucopyranoside (**12**) (DMSO-$d_6$, 600 MHz)

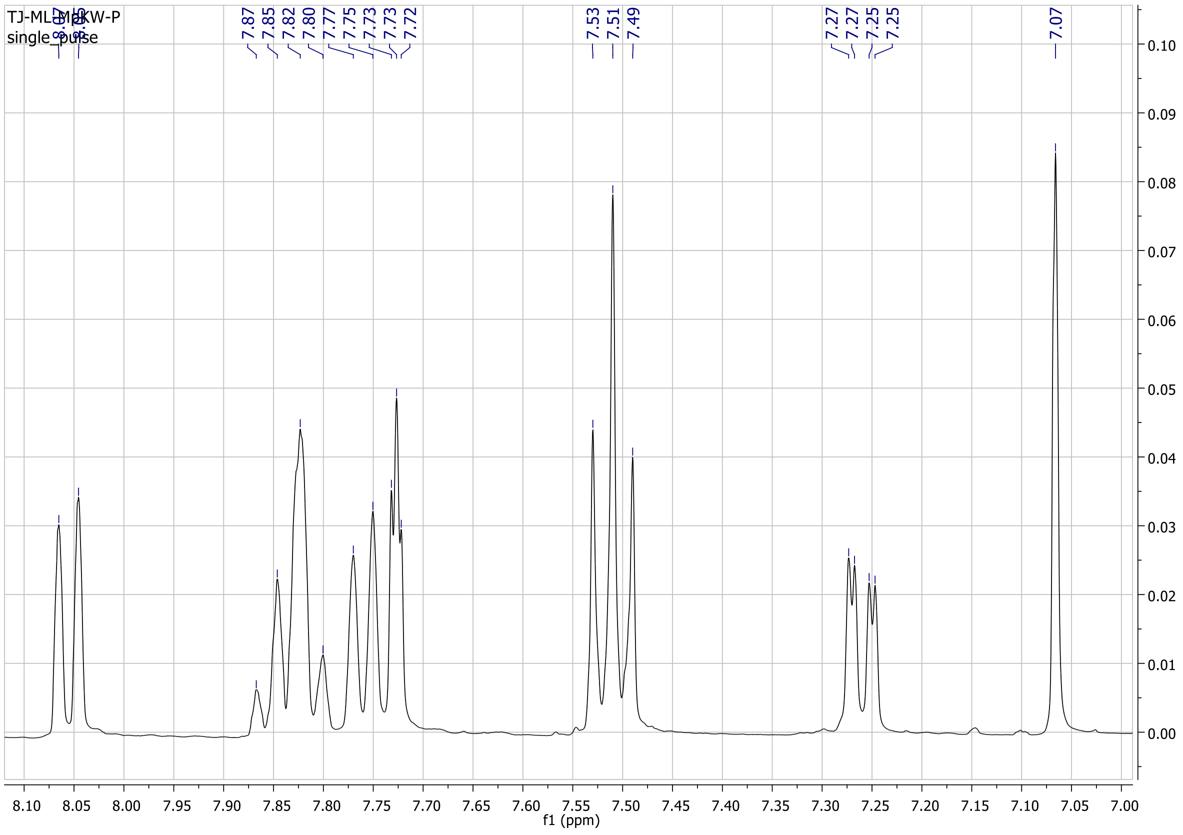

Fig.S43. Glucopyranoside part of the $^{1}$H NMR spectral flavone 3’-*O*-β-D-(4”-*O*-methyl)-glucopyranoside (**12**) (DMSO-$d_6$, 600 MHz)

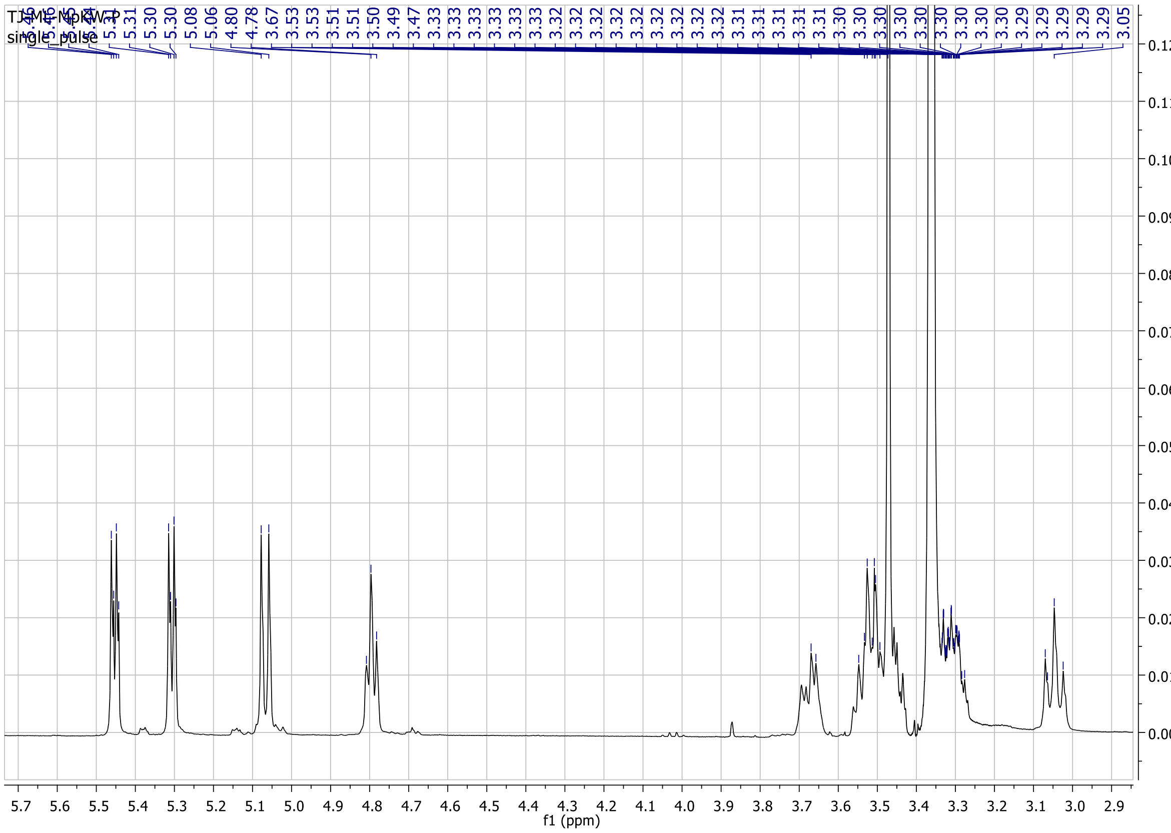

Fig.S44. $^{13}$C NMR spectral of flavone 3’-*O*-β-D-(4”-*O*-methyl)-glucopyranoside (**12**) (DMSO-$d_6$, 151 MHz)

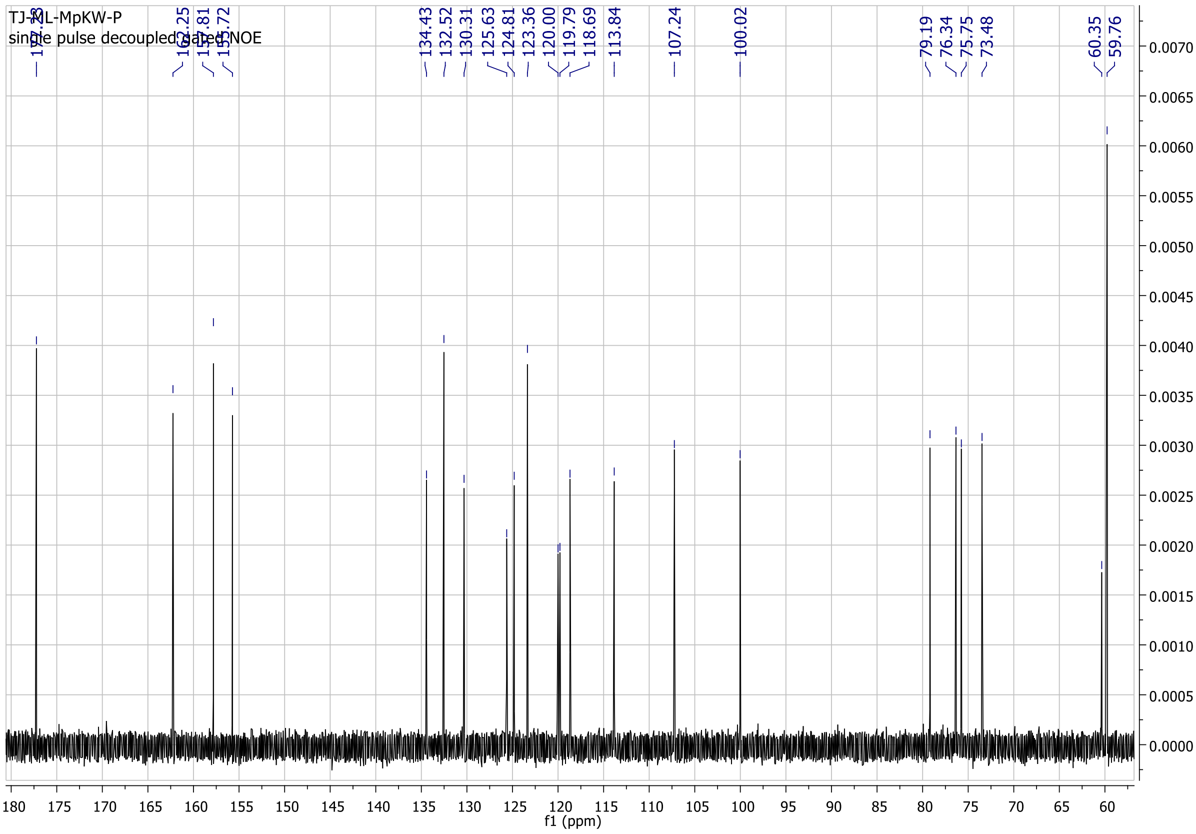

Fig.S45. HMQC spectral of flavone 3'-*O*-β-D-(4"-*O*-methyl)-glucopyranoside (**12**) (DMSO-$d_6$, 151 MHz)

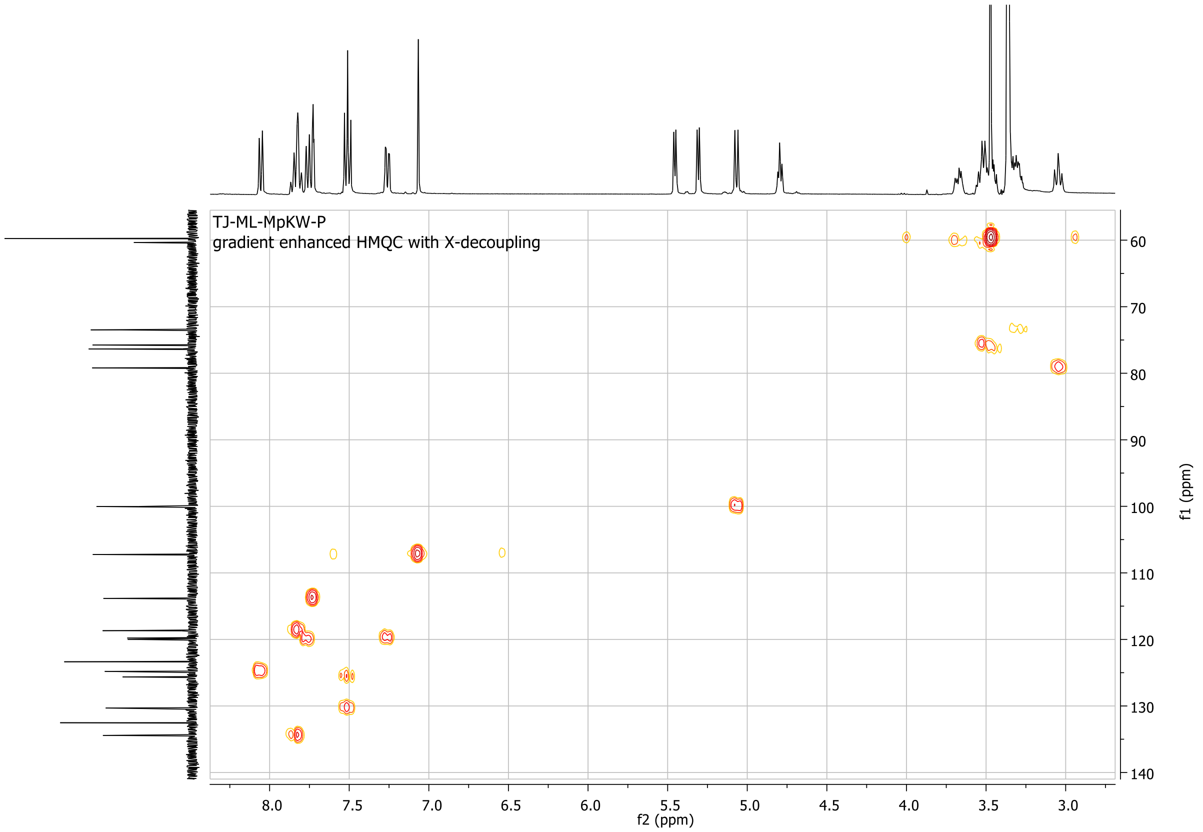


Fig.S46. HMBC spectral of flavone 3'-*O*-β-D-(4"-*O*-methyl)-glucopyranoside (**12**) (DMSO-$d_6$, 151 MHz)

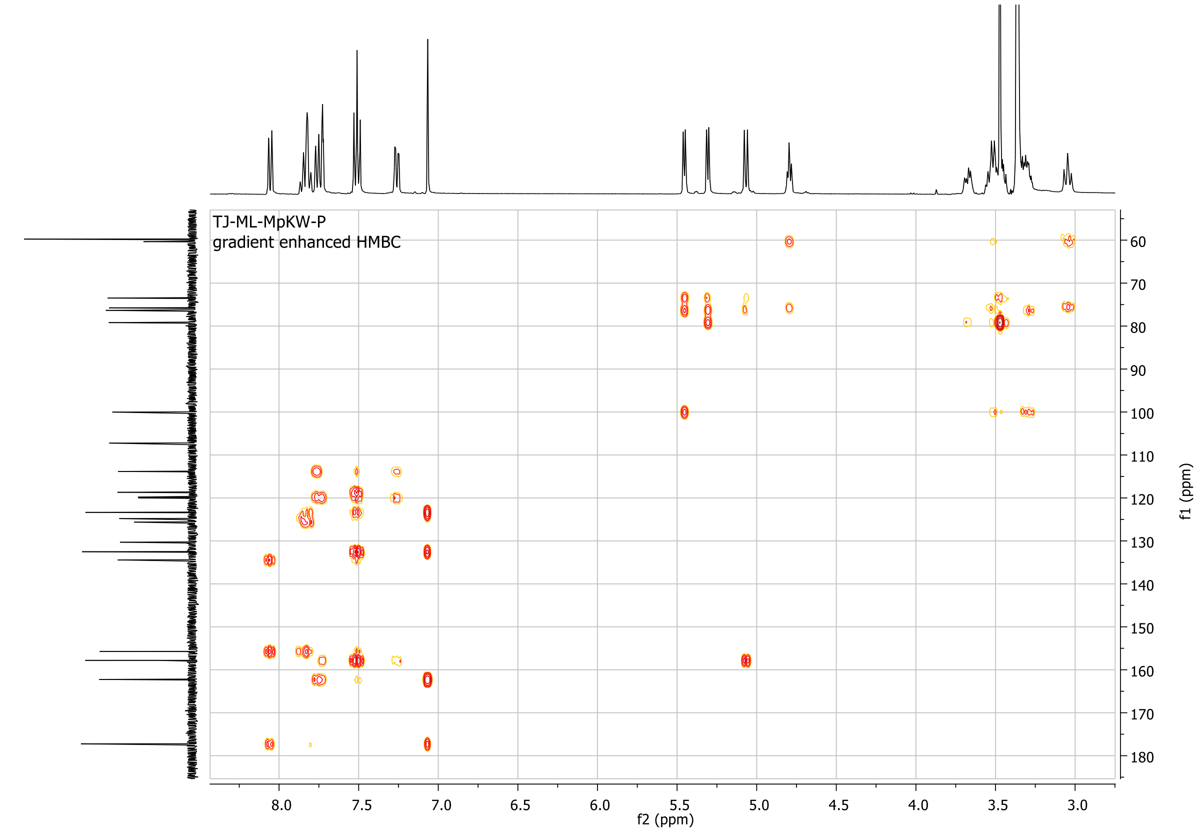

Fig.S47. MS analysis 4’-hydroxyflavone (**13**)

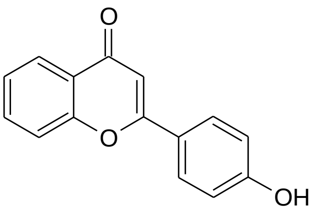


Molecular Formula: $C_{15}H_{10}O_3$
Formula Weight: 238.2381
Precursor: 239.2000

CE: -15.0

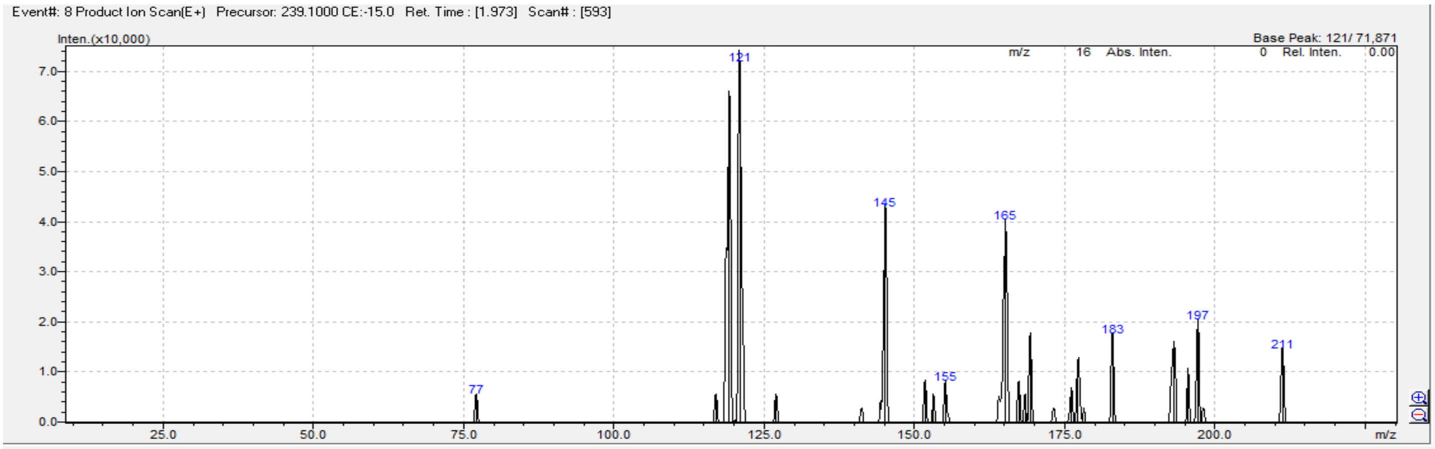


CE:-35.0

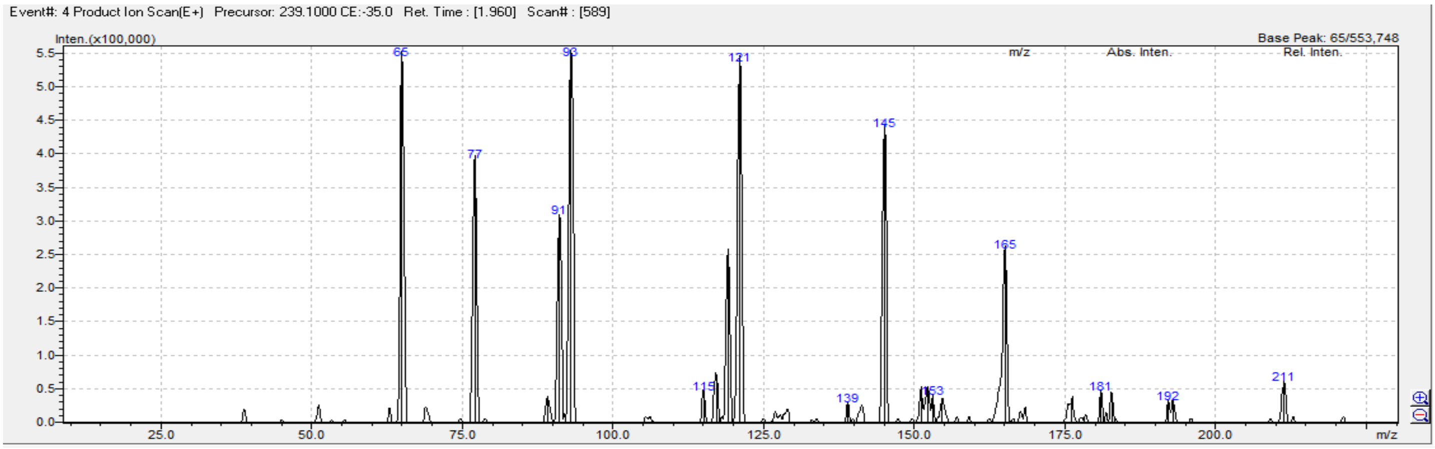


CE:-45.0

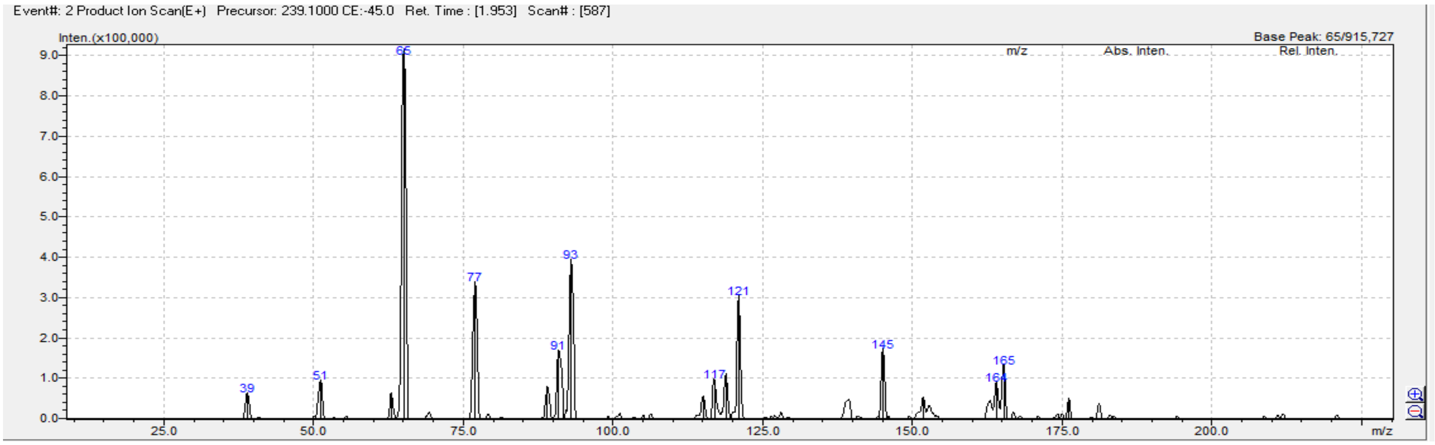

Fig.S48. $^{1}$H NMR spectral of 4'-hydroxyflavone (**13**) (DMSO-$d_6$, 600 MHz)

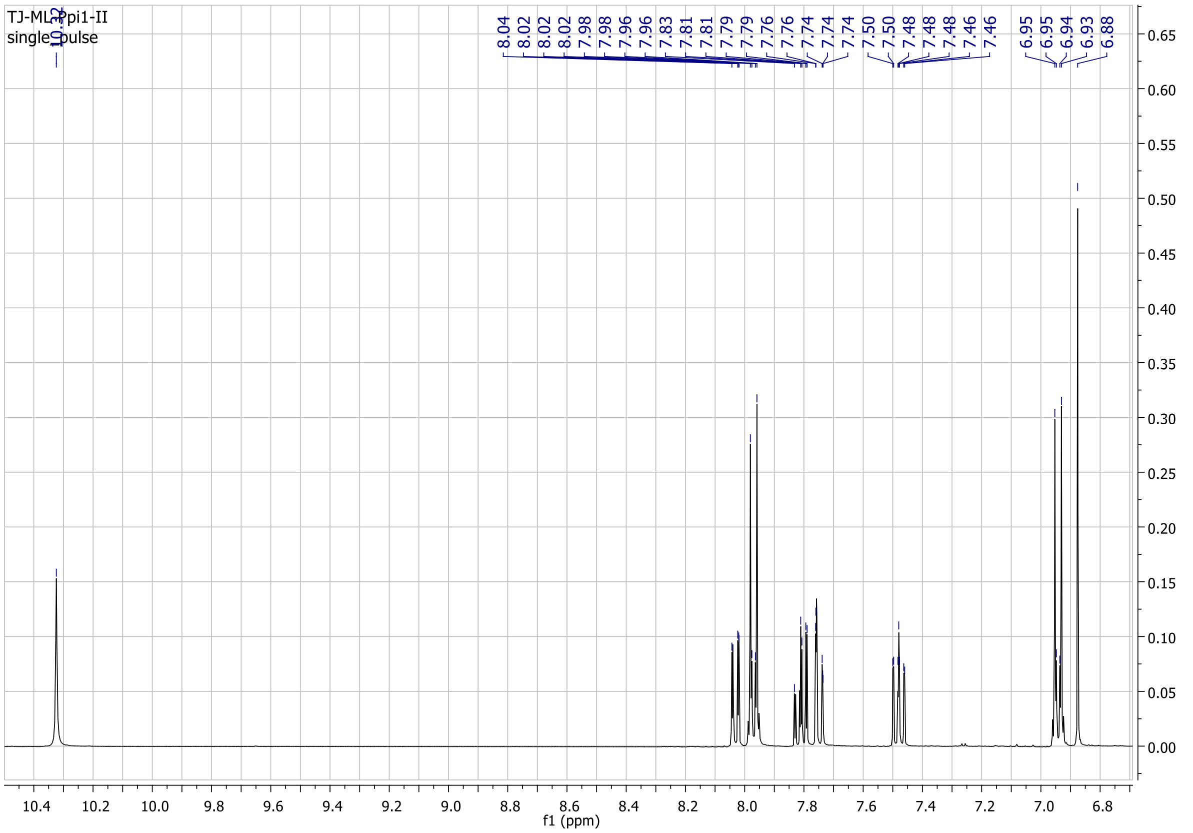


Fig.S49. $^{13}$C NMR spectral of 4'-hydroxyflavone (**13**) (DMSO-$d_6$, 151 MHz)

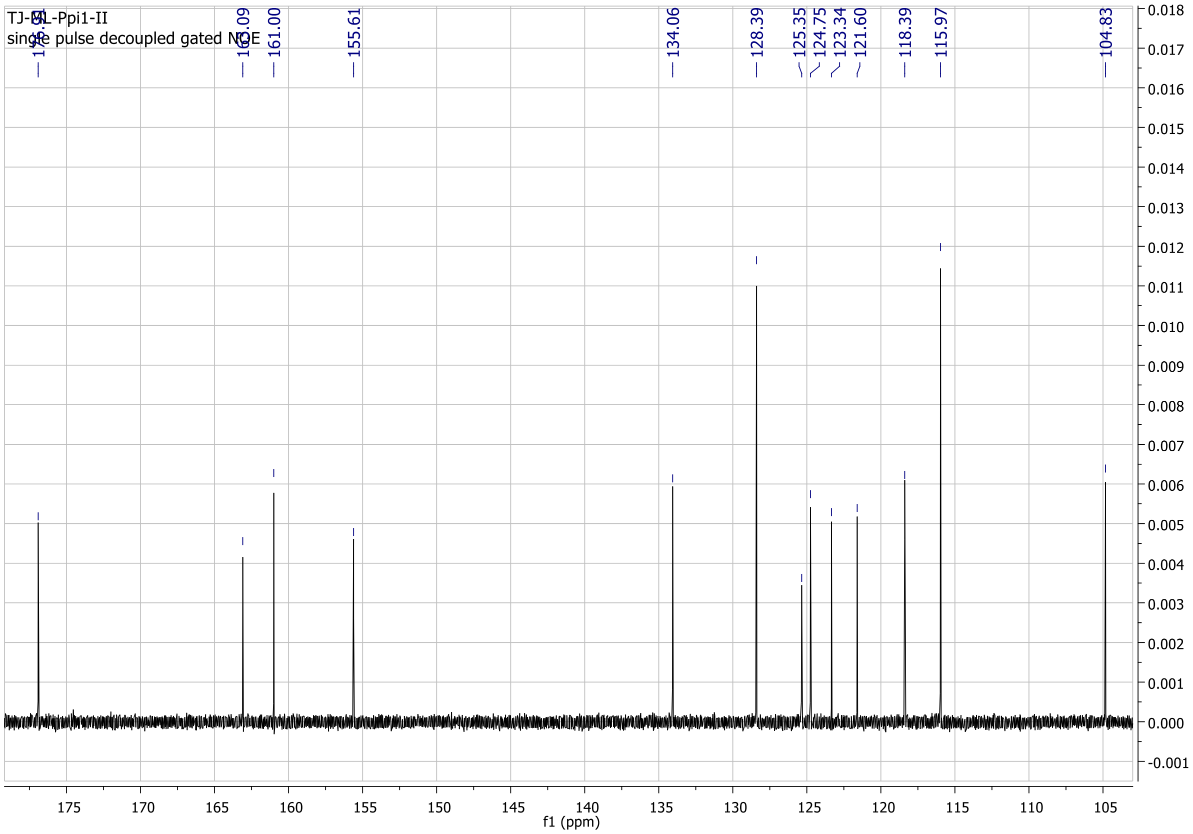

Fig.S50. COSY spectral of 4'-hydroxyflavone (**13**) (DMSO-$d_6$, 151 MHz)

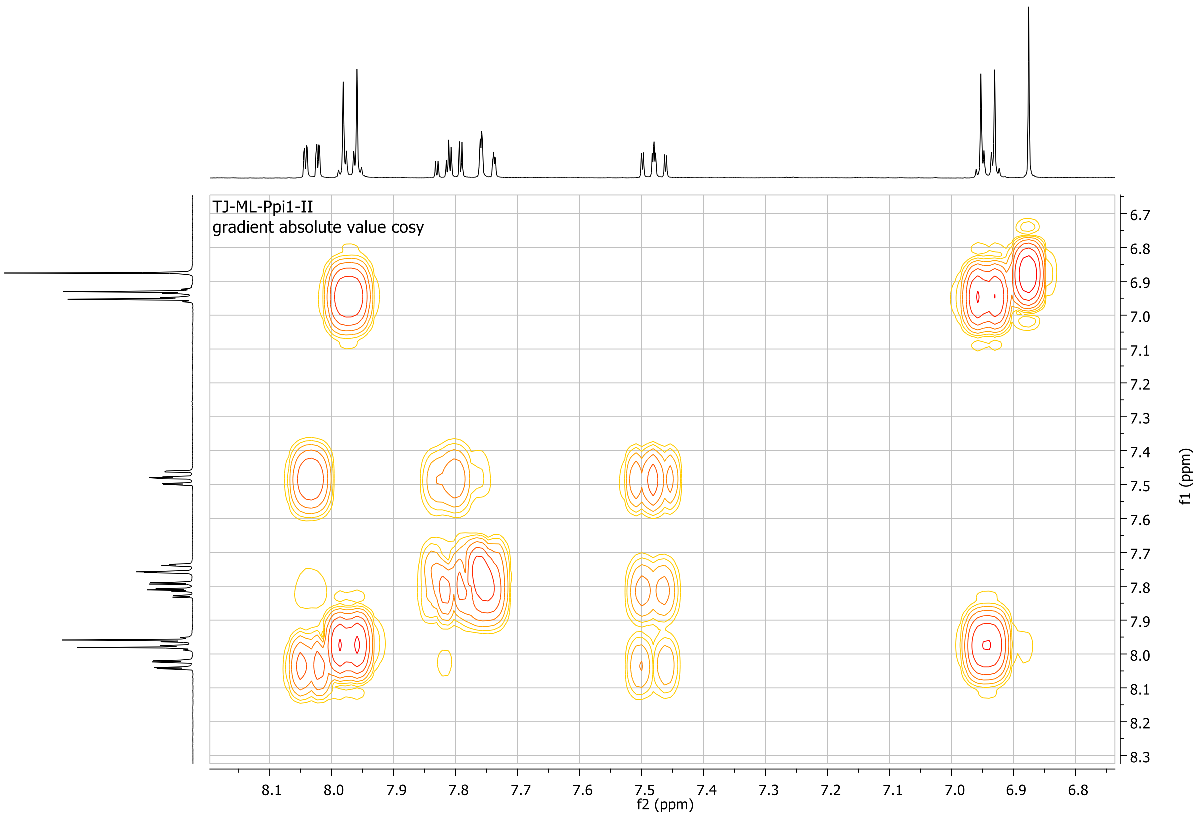


Fig.S51. HMQC spectral of 4'-hydroxyflavone (**13**) (DMSO-$d_6$, 151 MHz)

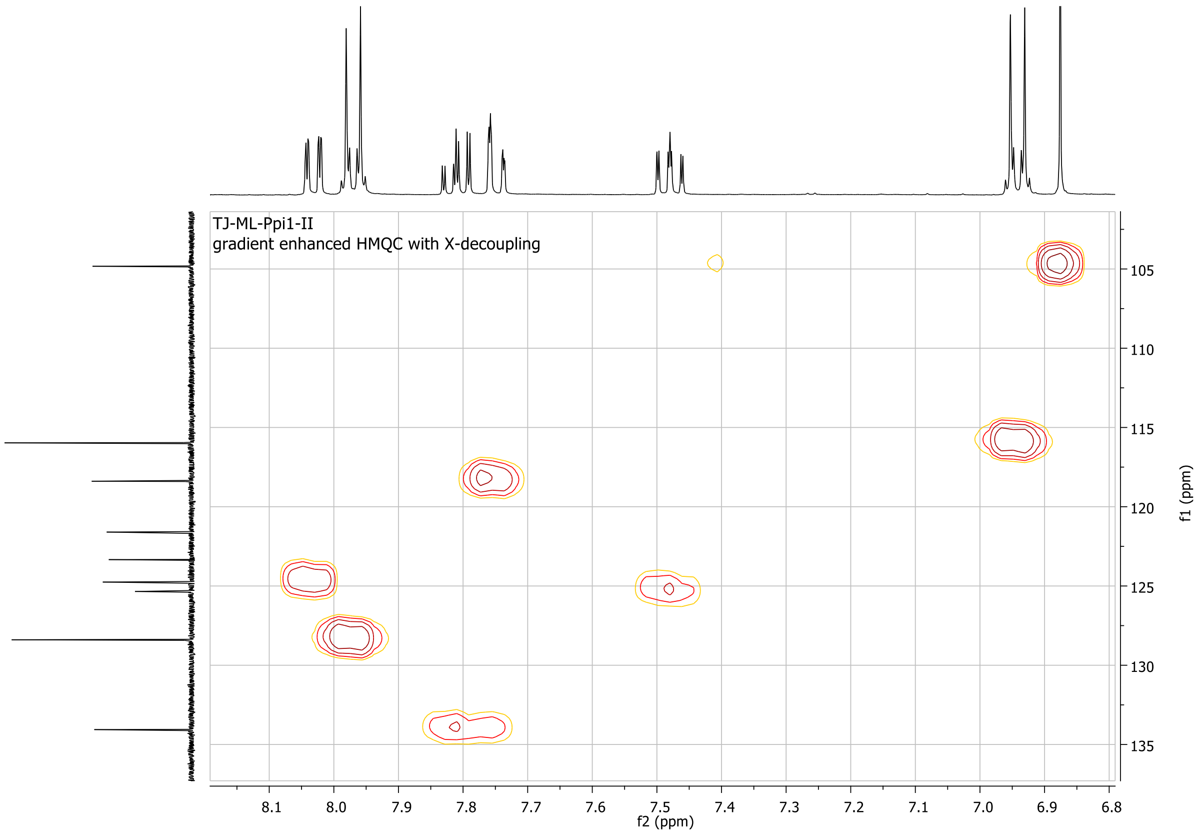

Fig.S52. HMBC spectral of 4’-hydroxyflavone (**13**) (DMSO-$d_6$, 151 MHz)

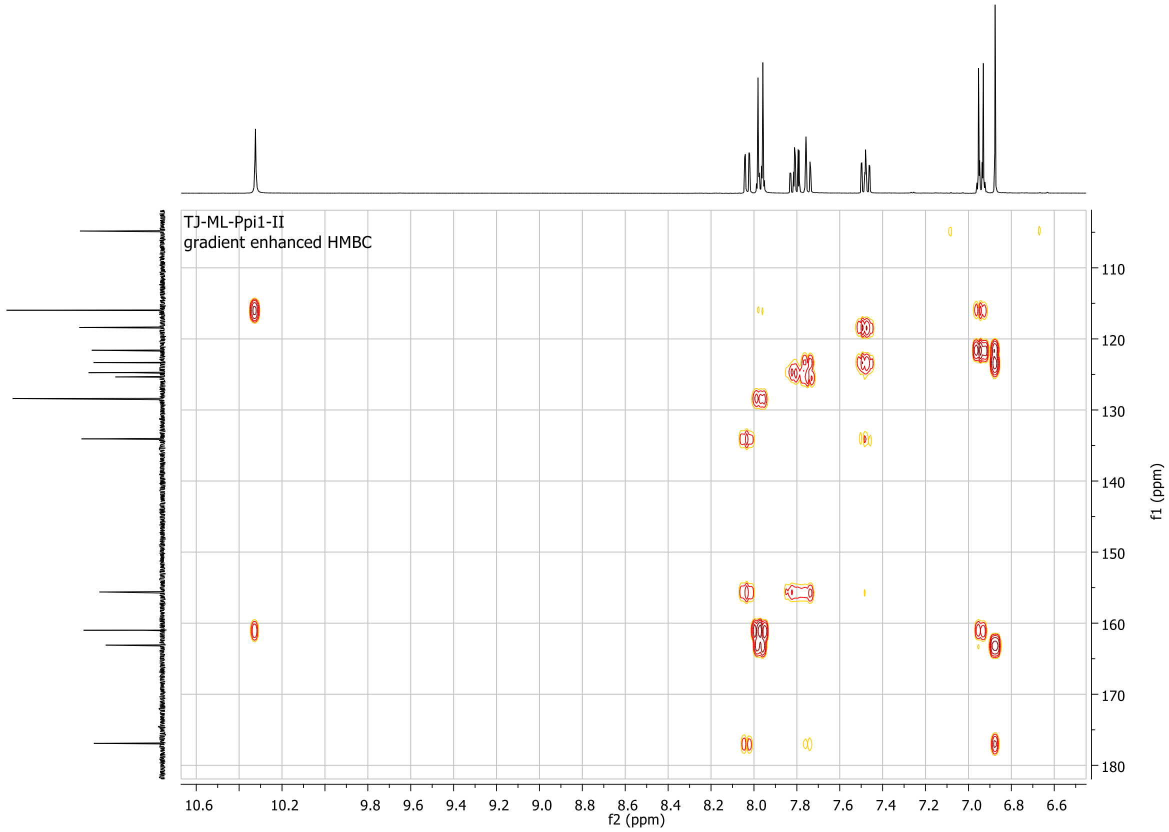

Fig.S53. MS analysis flavone 4'-*O*-β-D-(4''-*O*-methyl)-glucopyranoside (**14**)

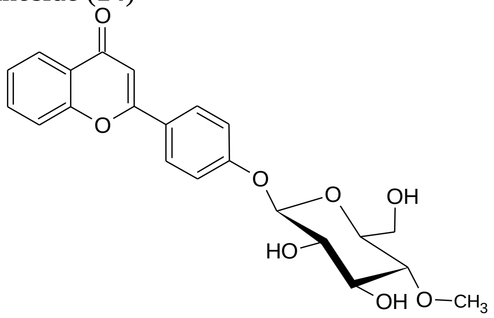


Molecular Formula = $C_{22}H_{22}O_8$
Formula Weight = 414.40528
Precursor =415.4000

CE: -15.0

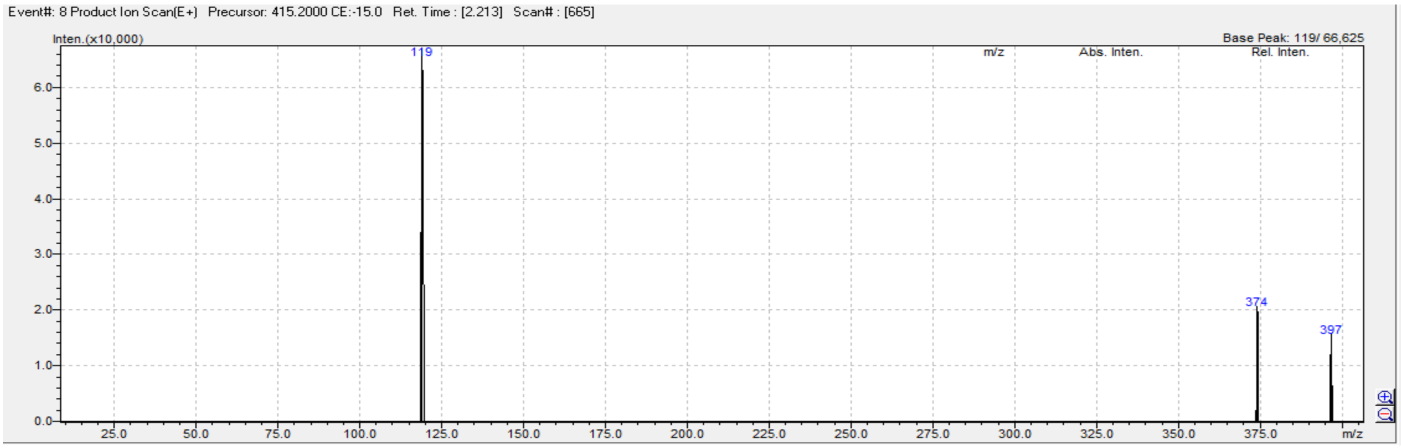


CE:-35.0

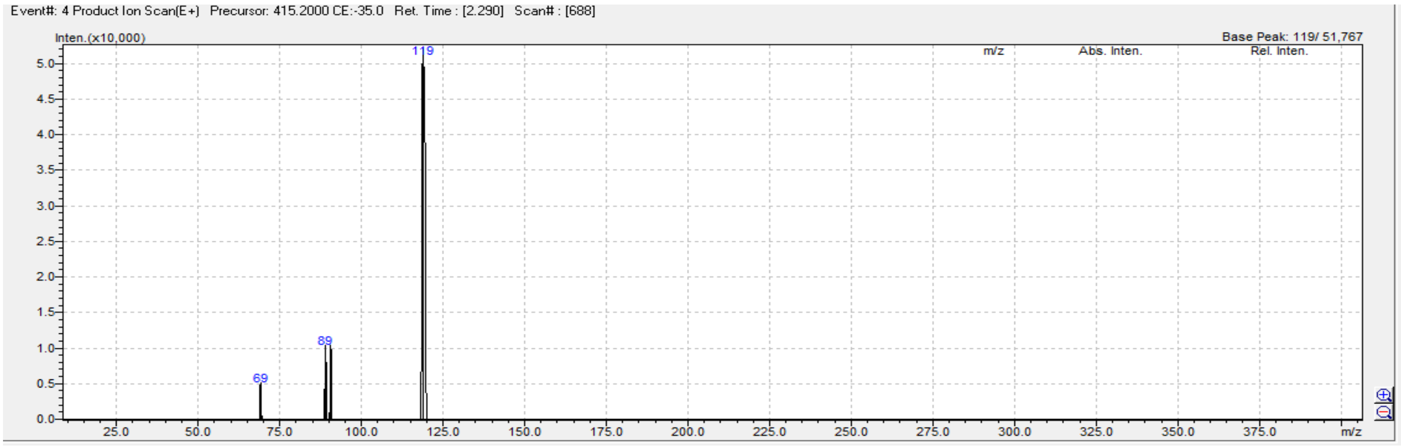


CE:-45.0

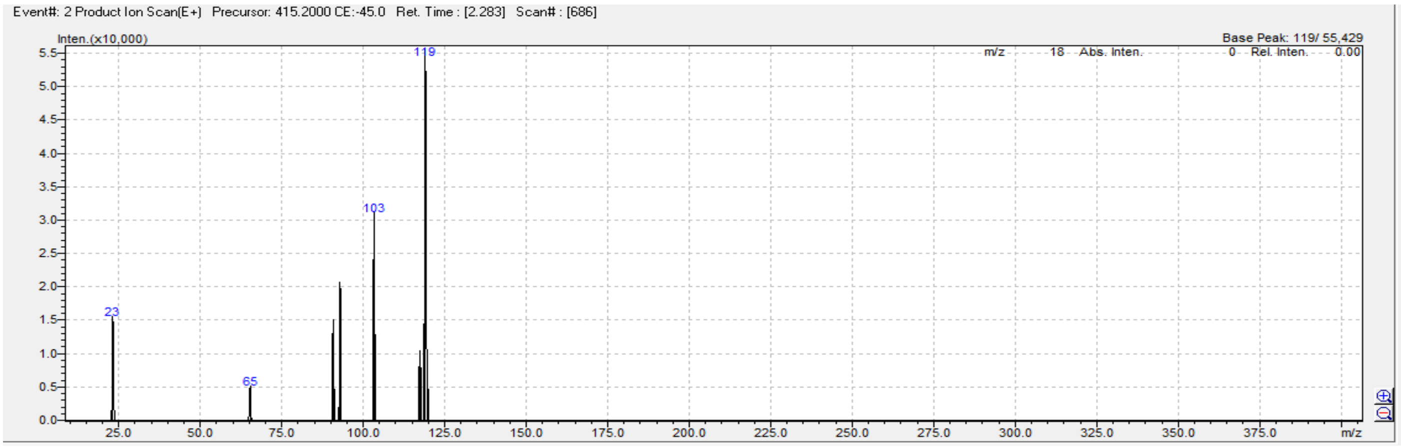

Fig.S54. $^{1}$H NMR spectral of flavone 4'-*O*-β-D-(4''-*O*-methyl)-glucopyranoside (**14**) (DMSO-$d_6$, 600 MHz)

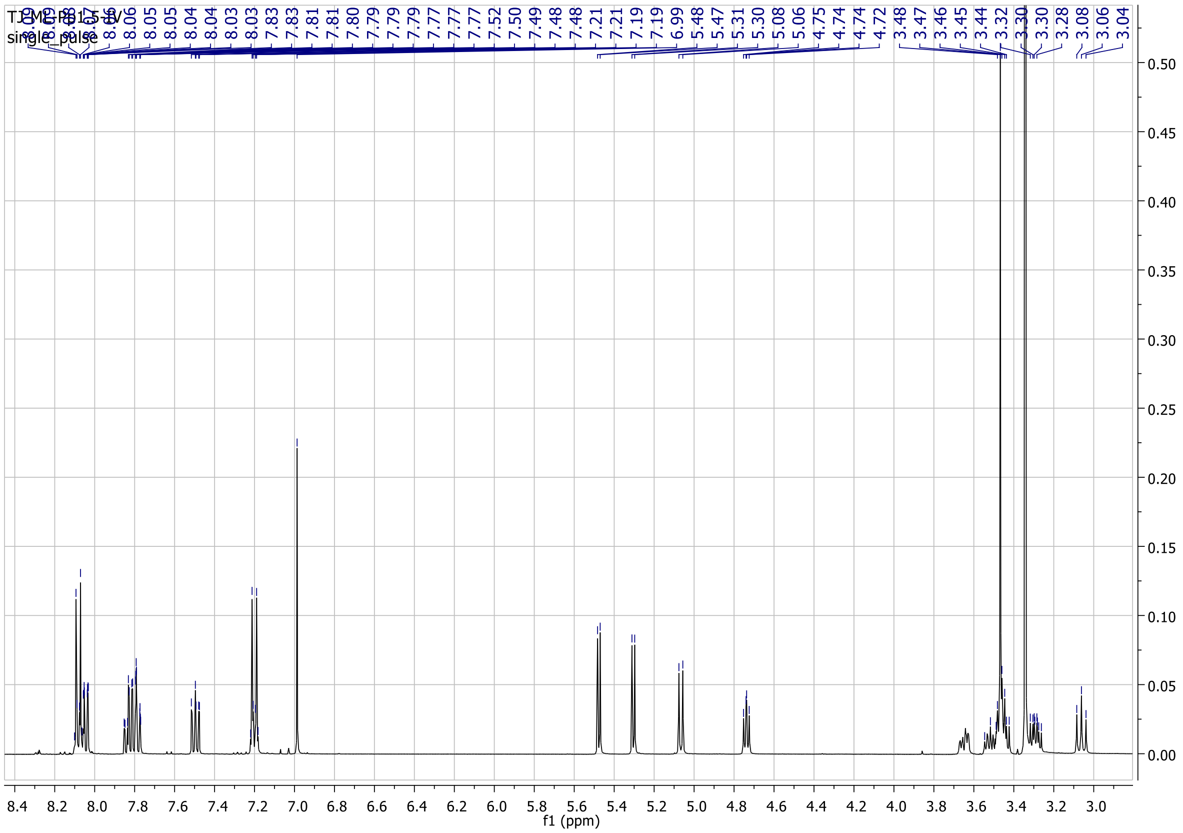


Fig.S55. Flavone part of the $^{1}$H NMR spectral flavone 4'-*O*-β-D-(4''-*O*-methyl)-glucopyranoside (**14**) (DMSO-$d_6$, 600 MHz)

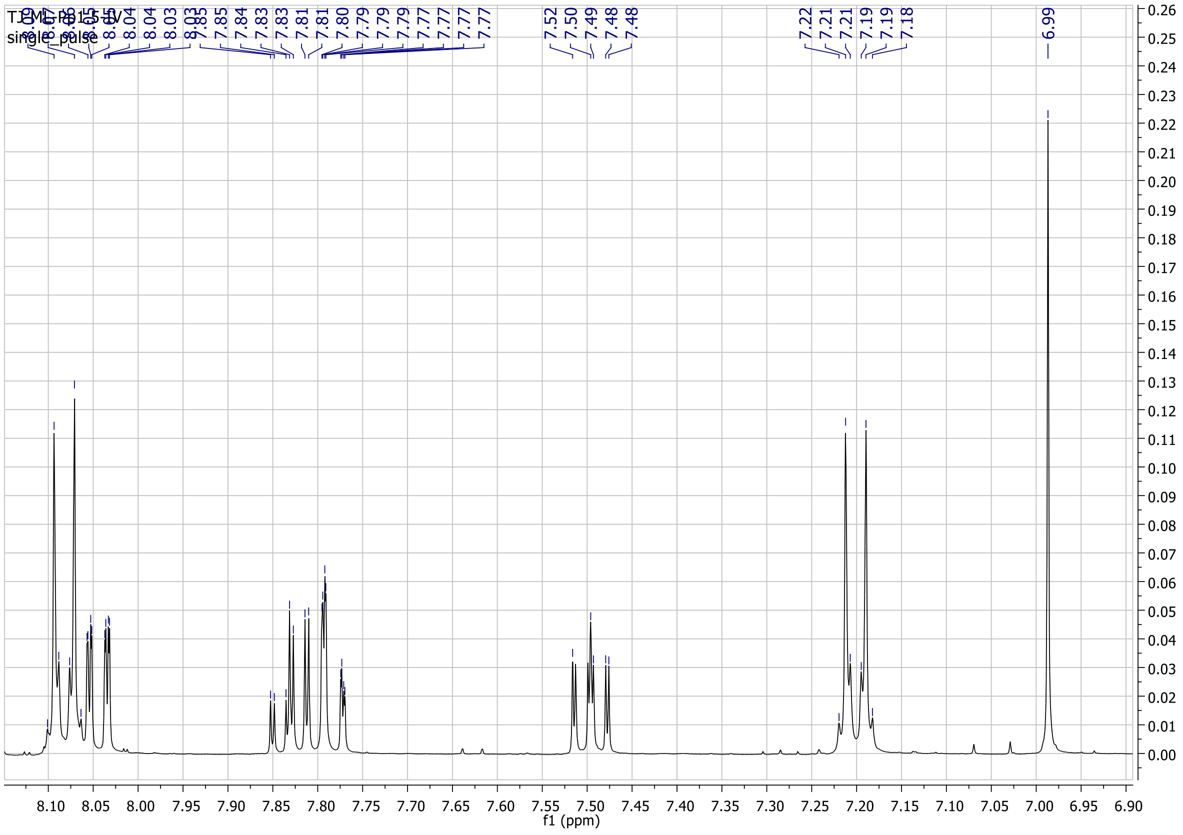

Fig.S56. Glucopyranoside part of the $^{1}$H NMR spectral flavone 4'-*O*-β-D-(4"-*O*-methyl)-glucopyranoside (**14**) (DMSO-$d_6$, 600 MHz)

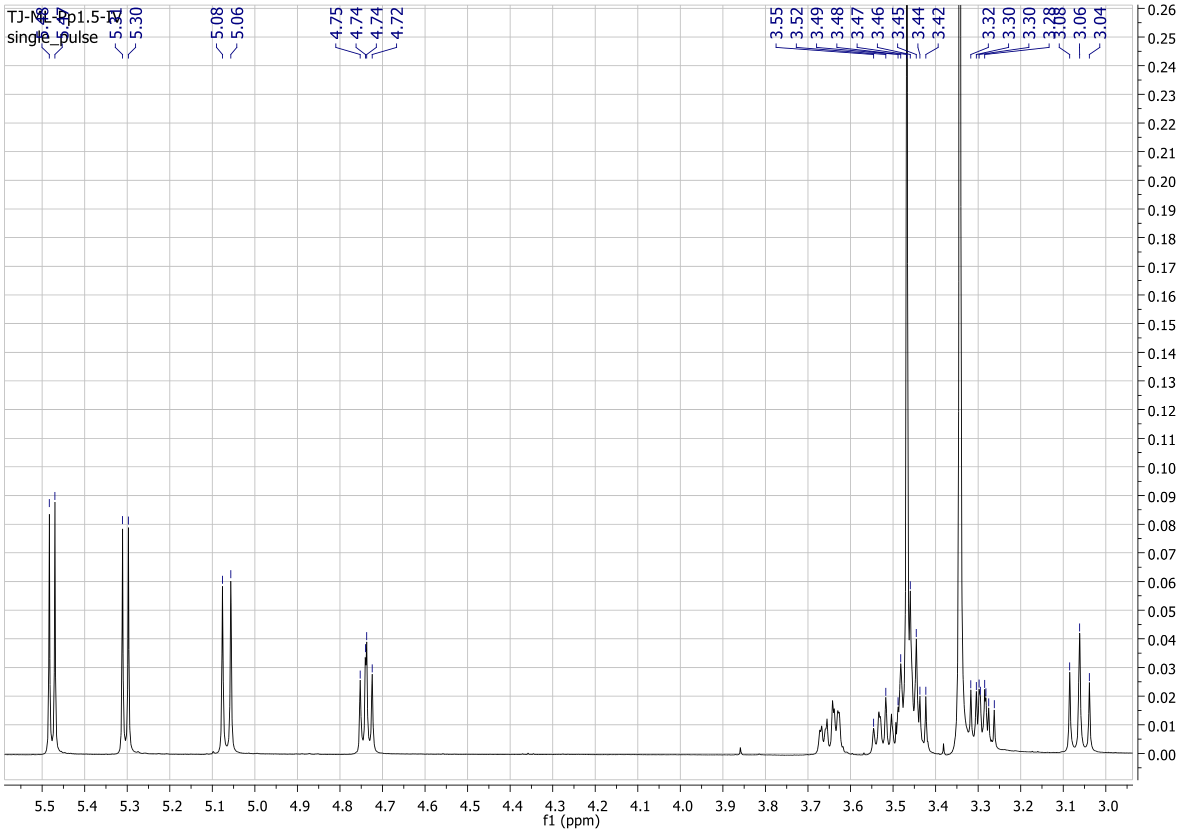


Fig.S57. $^{13}$C NMR spectral of flavone 4'-*O*-β-D-(4"-*O*-methyl)-glucopyranoside (**14**) (DMSO-$d_6$, 151 MHz)

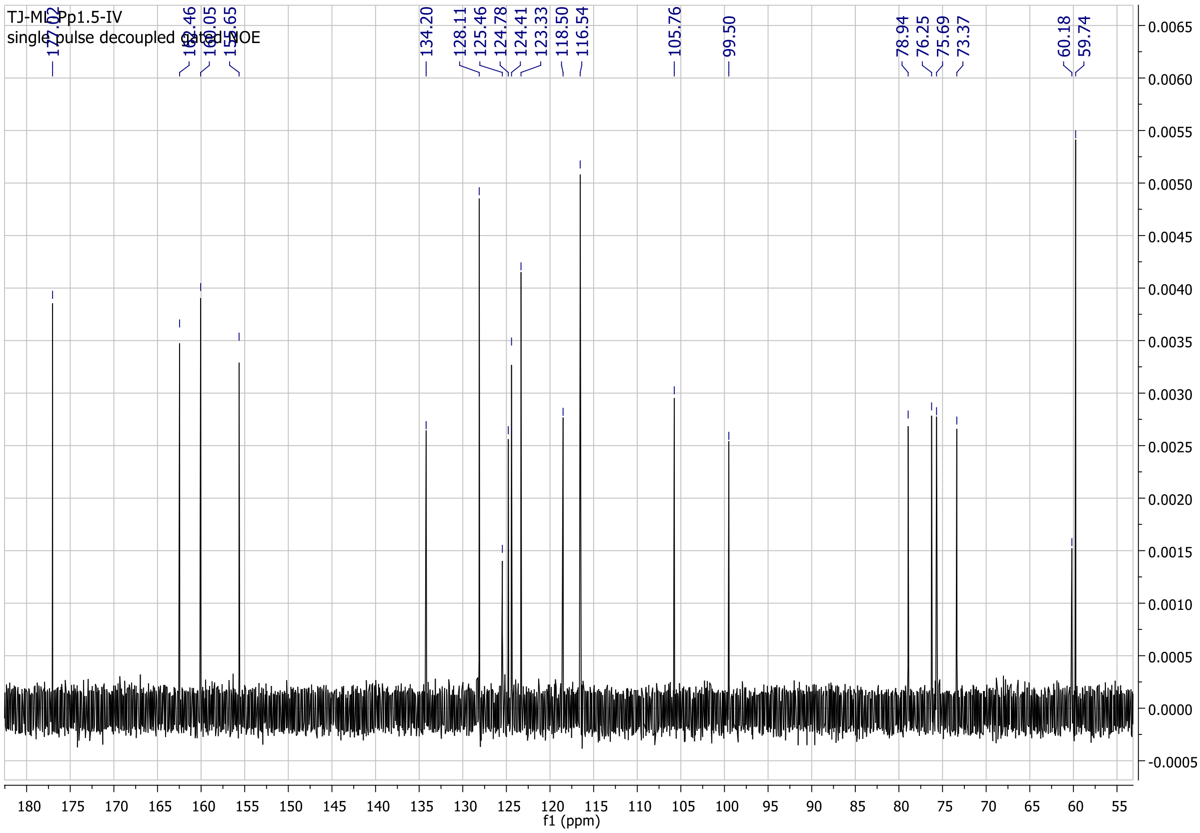

Fig.S58. COSY spectral of flavone 4’-*O*-β-D-(4”-*O*-methyl)-glucopyranoside (**14**) (DMSO-$d_6$, 151 MHz)

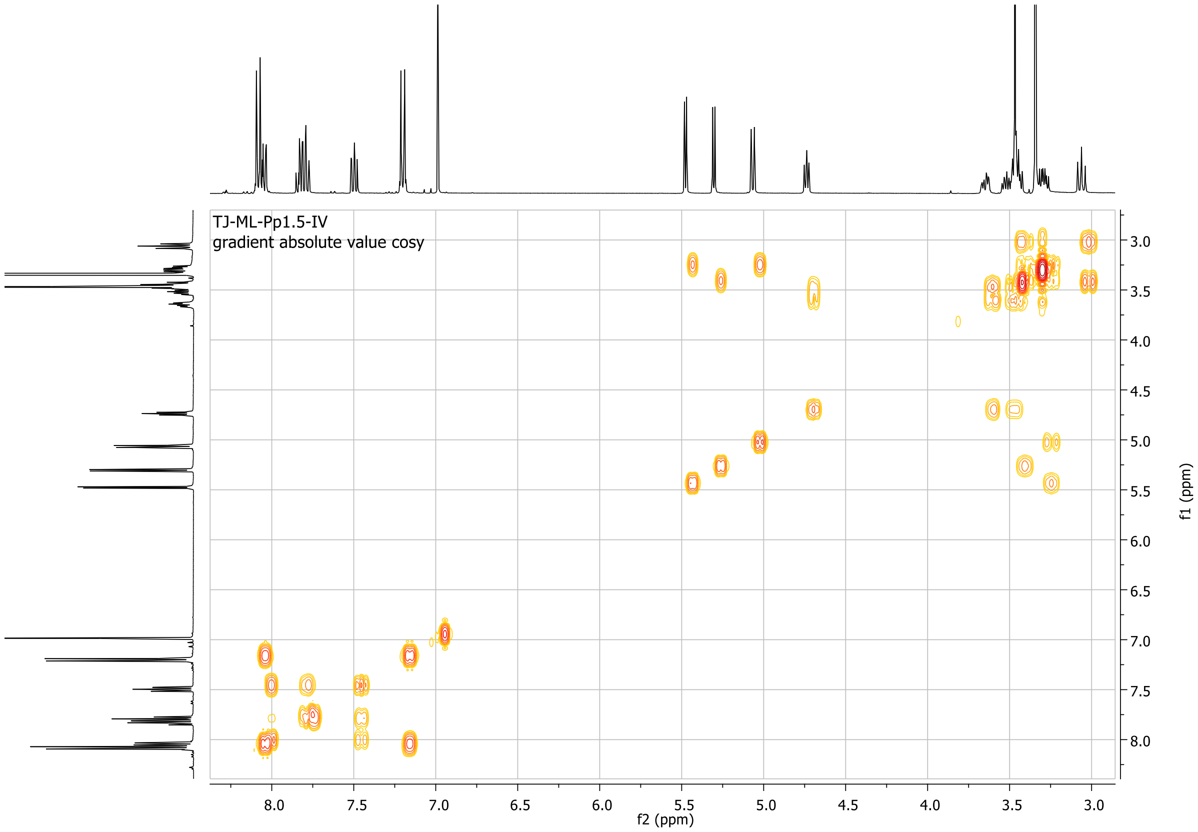


Fig.S59. HMQC spectral of flavone 4’-*O*-β-D-(4”-*O*-methyl)-glucopyranoside (**14**) (DMSO-$d_6$, 151 MHz)

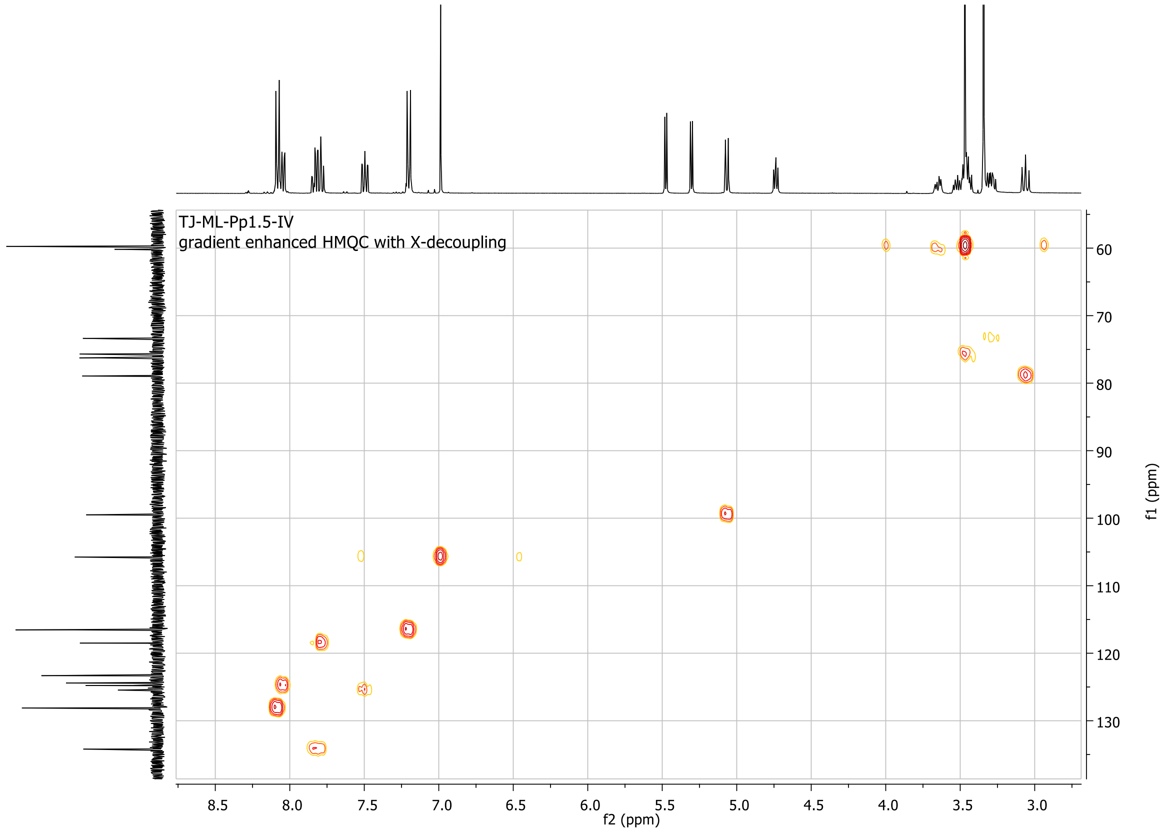

Fig.S60. HMBC spectral of flavone 4'-*O*-β-D-(4''-*O*-methyl)-glucopyranoside (**14**) (DMSO-$d_6$, 151 MHz)

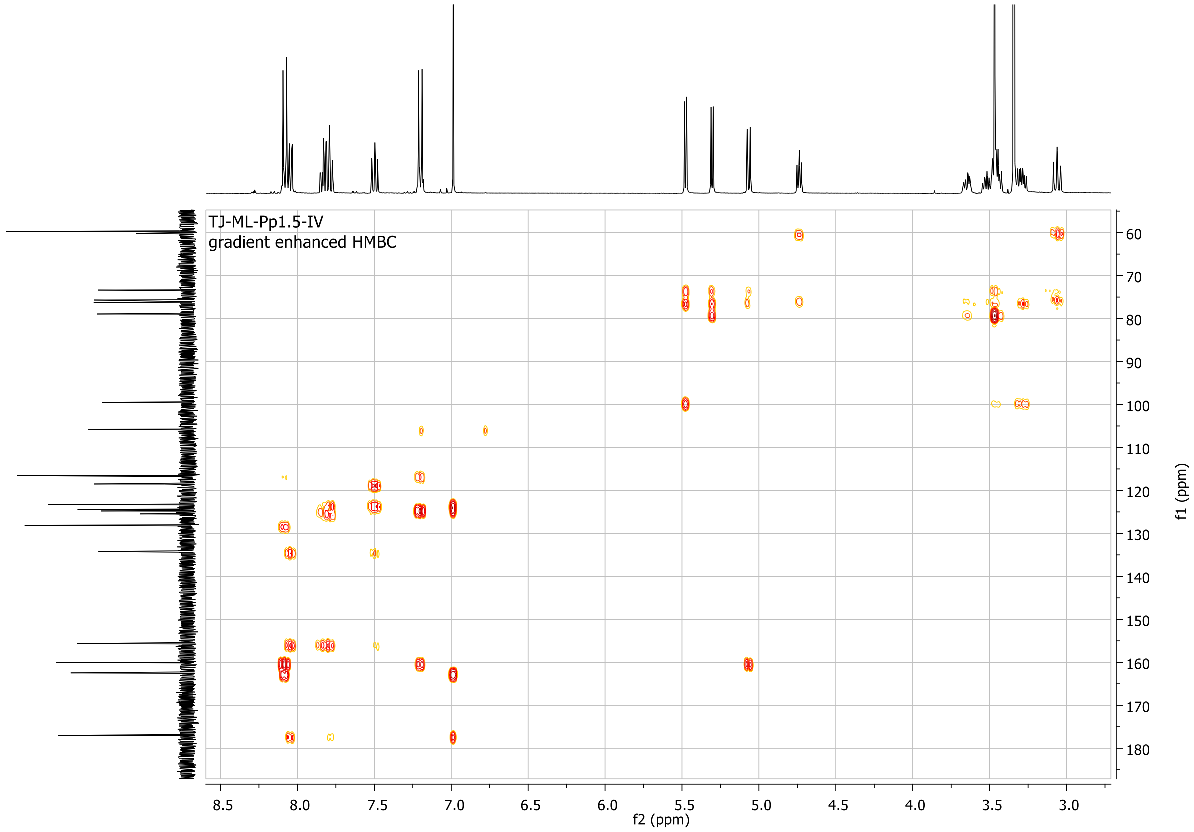

Fig.S61. MS analysis 5’-methoxyflavone 2’-*O*-β-D-(4”-*O*-methyl)-glucopyranoside (**15**)

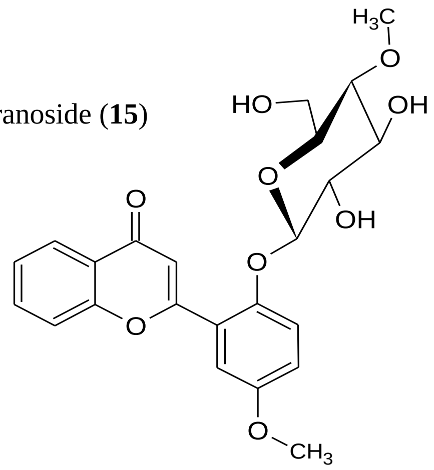


Molecular Formula = $C_{23}H_{24}O_9$
Formula Weight = 444.43126
Precursor =445.4000

CE: -15.0

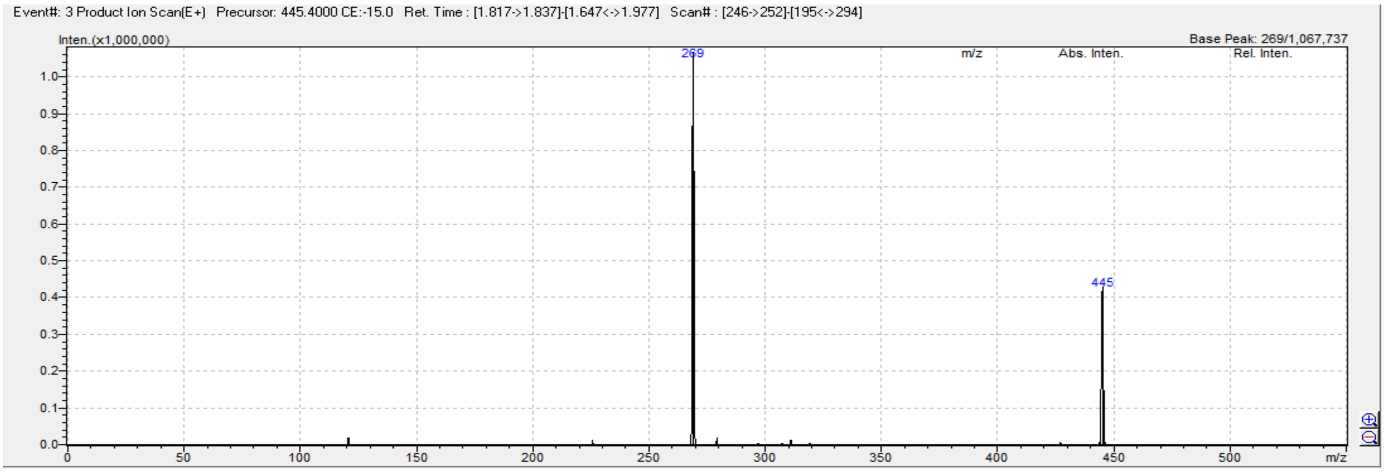


CE:-35.0

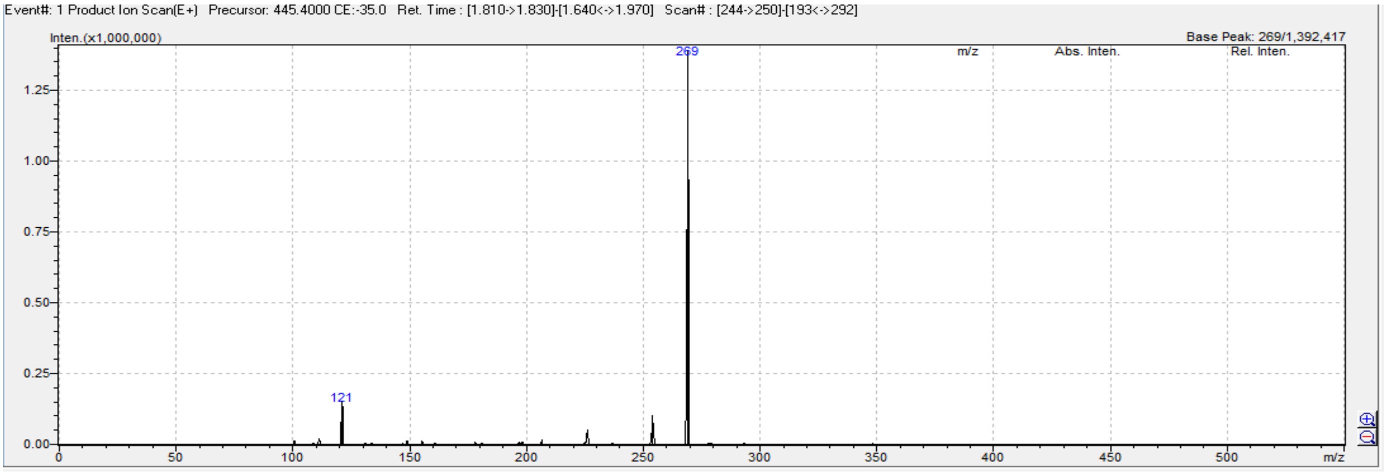


CE:-45.0

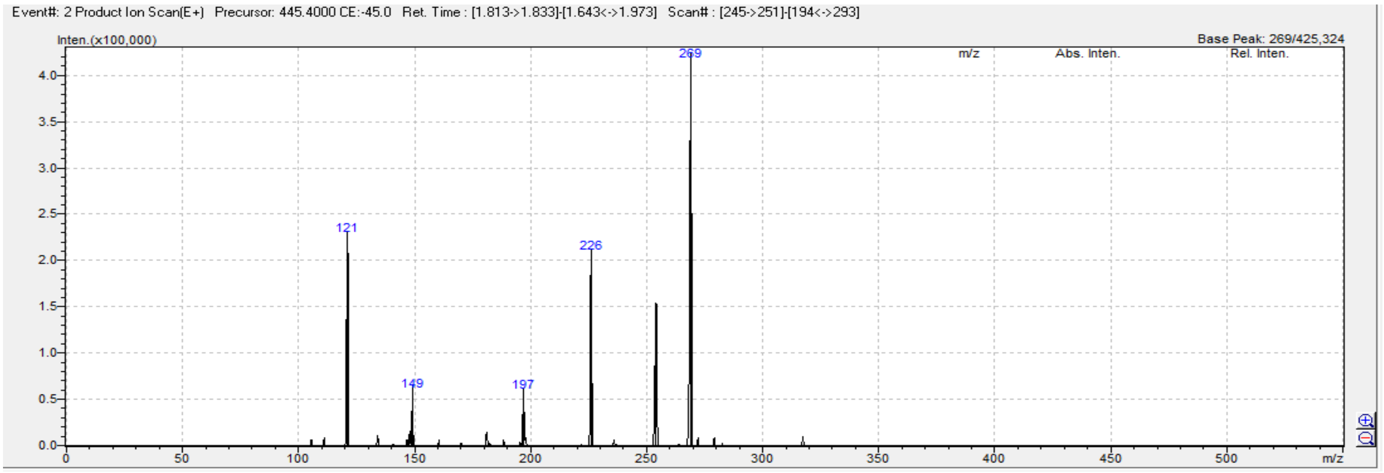

Fig.S62. $^{1}$H NMR spectral of 5'-methoxyflavone 2'-*O*-β-D-(4"-*O*-methyl)-glucopyranoside (**15**) (DMSO-$d_6$, 600 MHz)

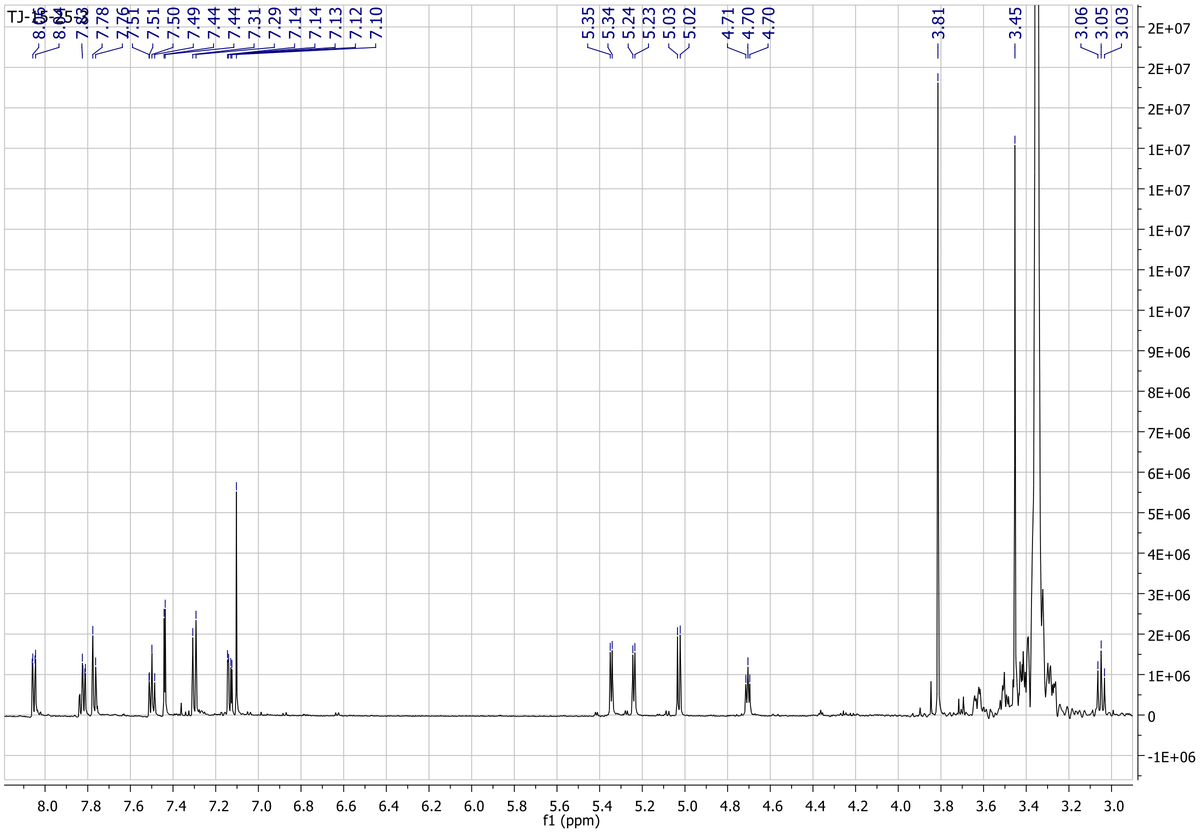

Fig.S63. Flavone part of the $^{1}$H NMR spectral 5'-methoxyflavone 2'-*O*-β-D-(4"-*O*-methyl)-glucopyranoside (**15**) (DMSO-$d_6$, 600 MHz)

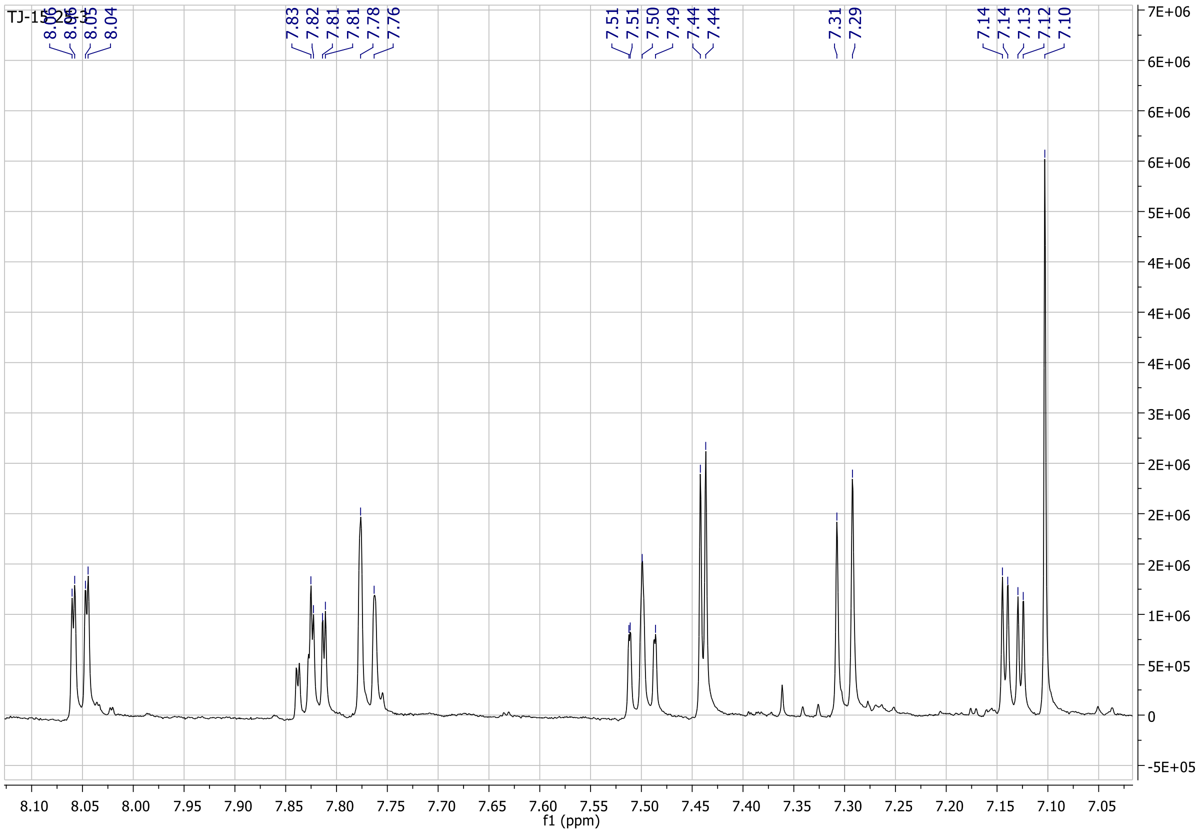

Fig.S64. Glucopyranoside part of the $^{1}$H NMR spectral 5'-methoxyflavone 2'-*O*-β-D-(4"-*O*-methyl)-glucopyranoside (**15**) (DMSO-$d_6$, 600 MHz)

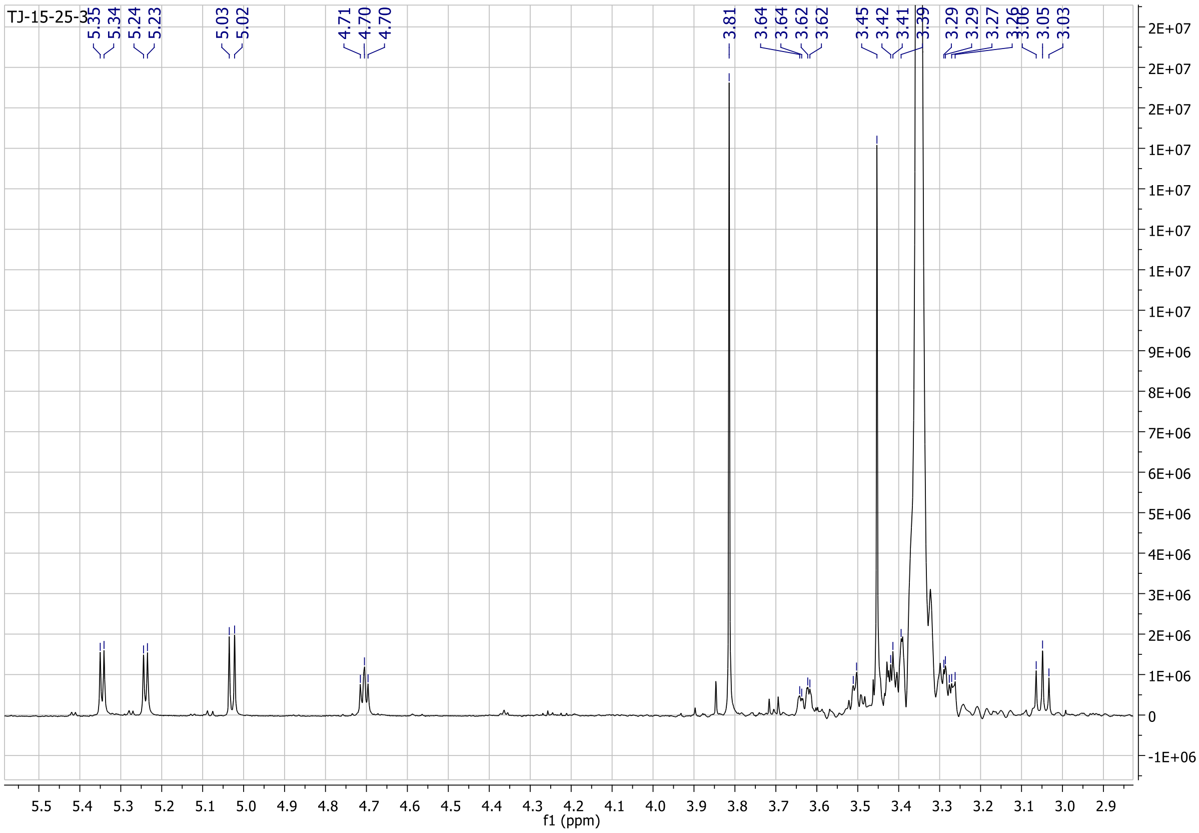


Fig.S65. $^{13}$C NMR spectral of 5'-methoxyflavone 2'-*O*-β-D-(4"-*O*-methyl)-glucopyranoside (**15**) (DMSO-$d_6$, 151 MHz)

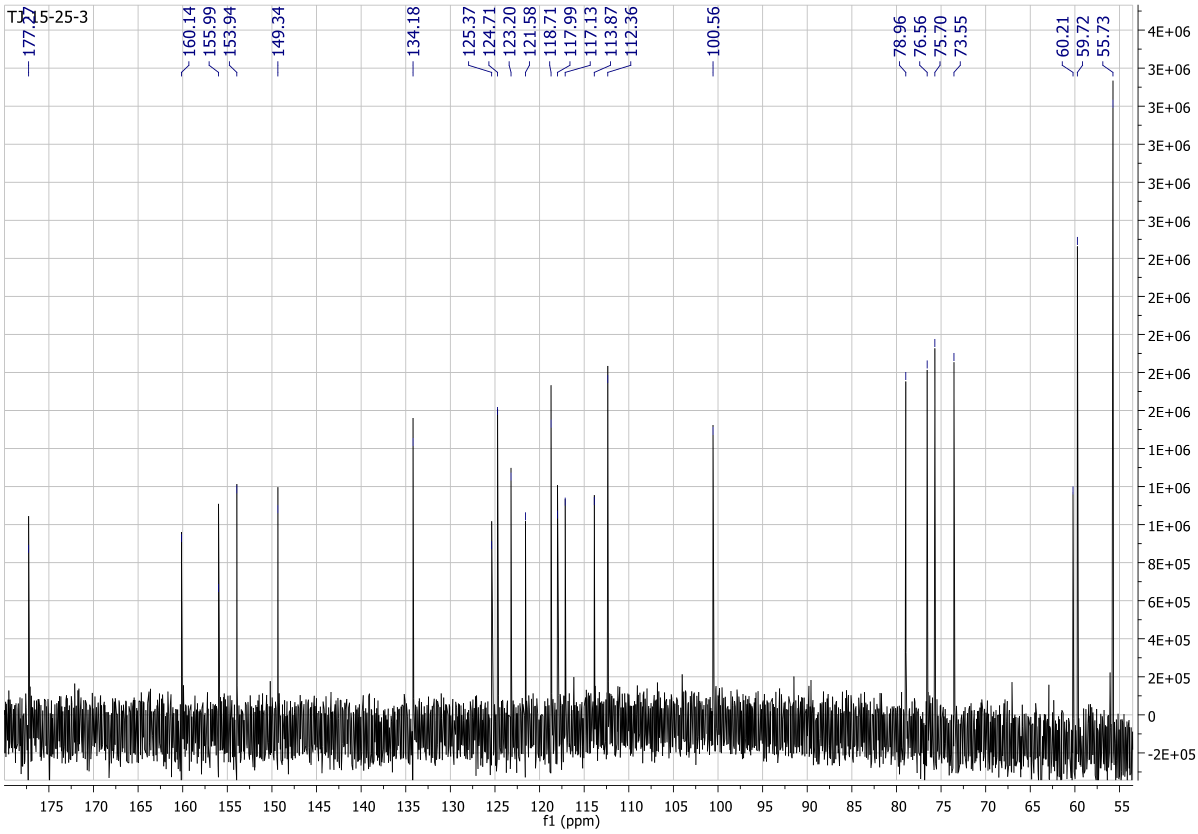

Fig.S66. COSY spectral of 5’-methoxyflavone 2’-*O*-β-D-(4”-*O*-methyl)-glucopyranoside (**15**) (DMSO-$d_6$, 151 MHz)

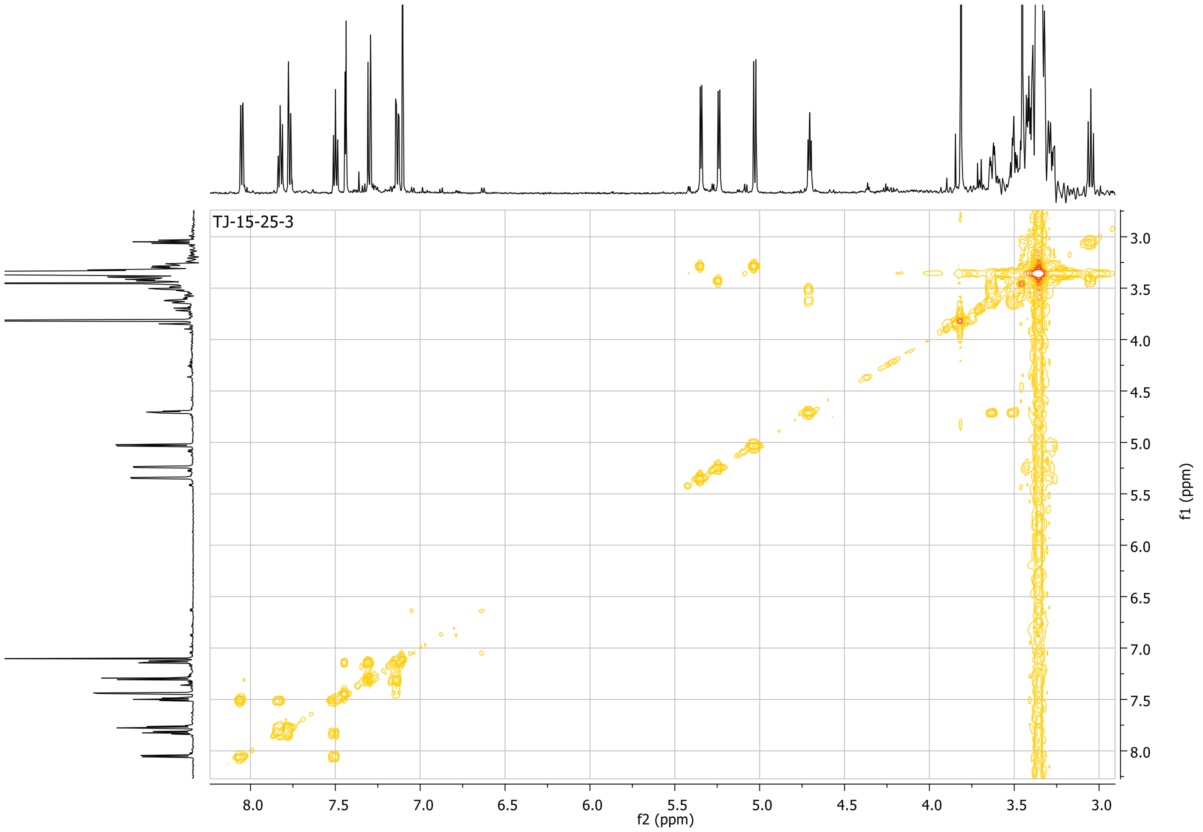


Fig.S67. HMQC spectral of 5’-methoxyflavone 2’-*O*-β-D-(4”-*O*-methyl)-glucopyranoside (**15**) (DMSO-$d_6$, 151 MHz)

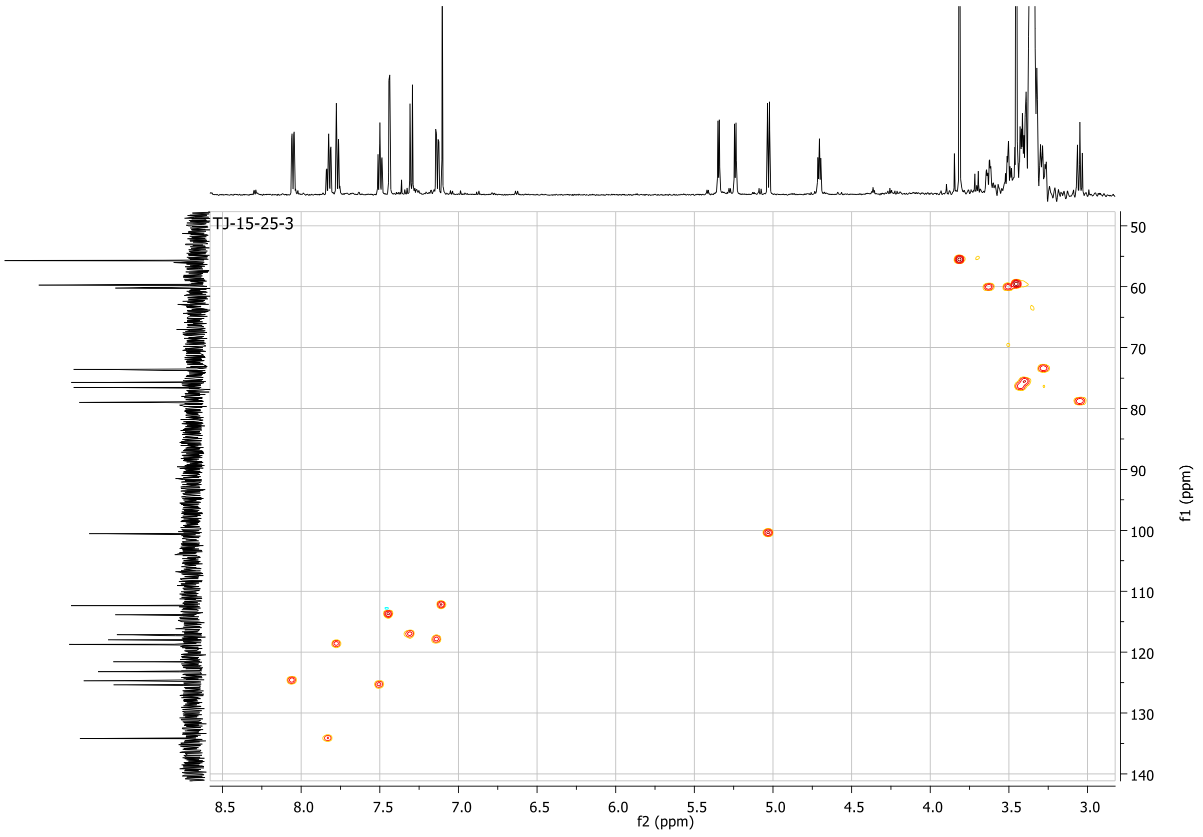

Fig.S68. HMBC spectral of 5’-methoxyflavone 2’-*O*-β-D-(4”-*O*-methyl)-glucopyranoside (**15**) (DMSO-$d_6$, 151 MHz)

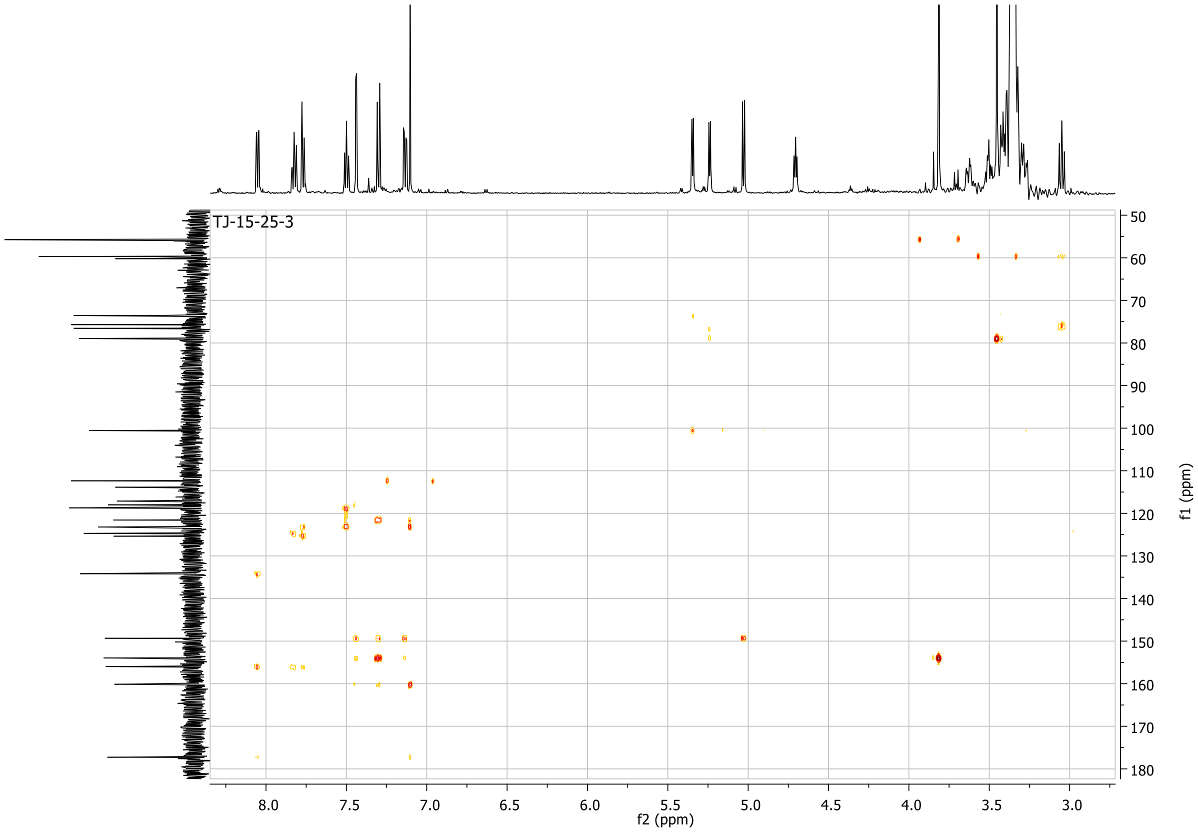

Fig.S69. MS analysis 2’,5’-dimethoxyflavone 4’-*O*-β-D-(4”-*O*-methyl)-glucopyranoside (**16**)

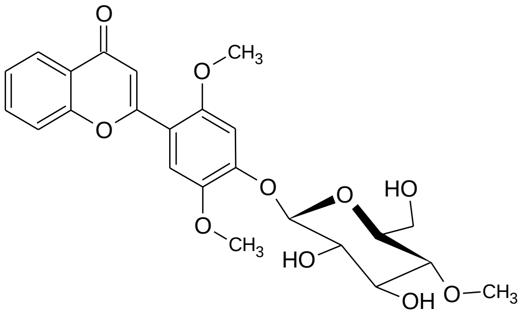


Molecular Formula = $C_{24}H_{26}O_{10}$
Formula Weight = 474.45724
Precursor: =475.4000

CE: -15

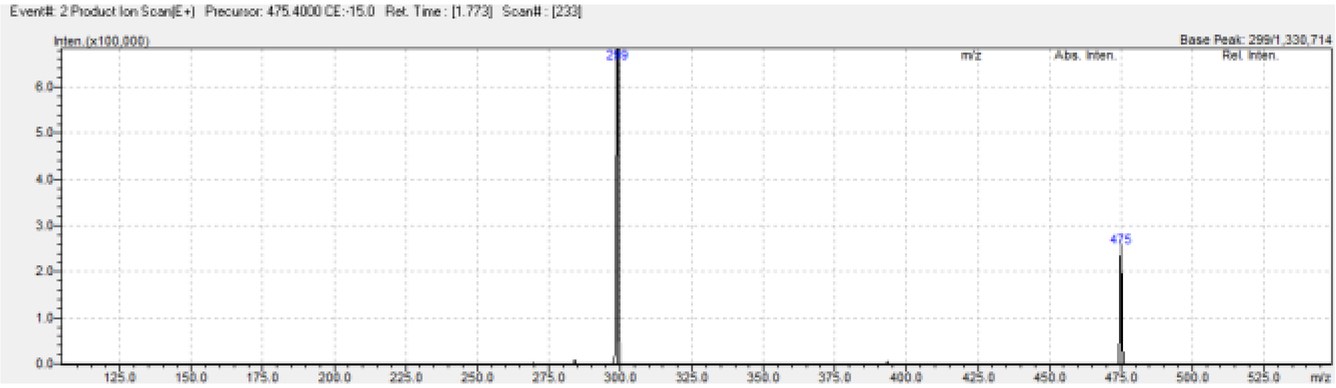


CE:-35

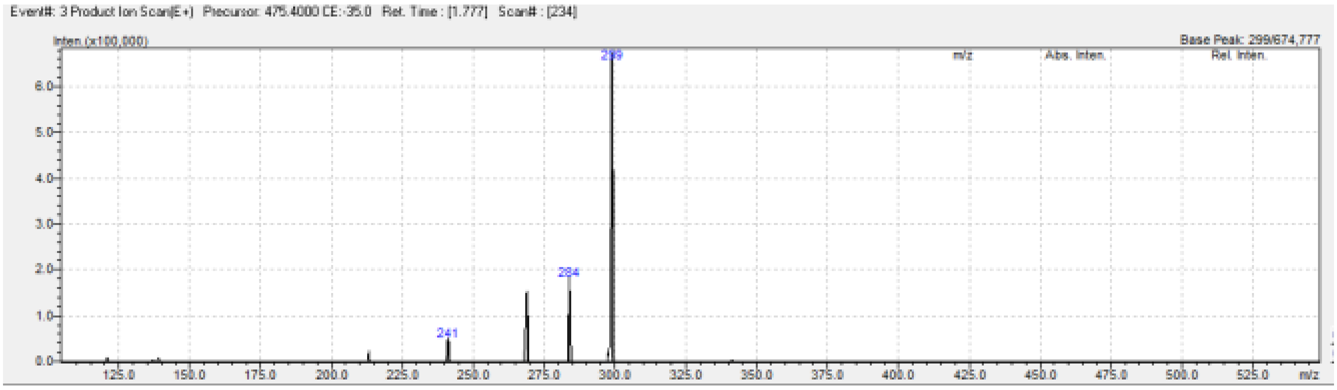


CE:-45

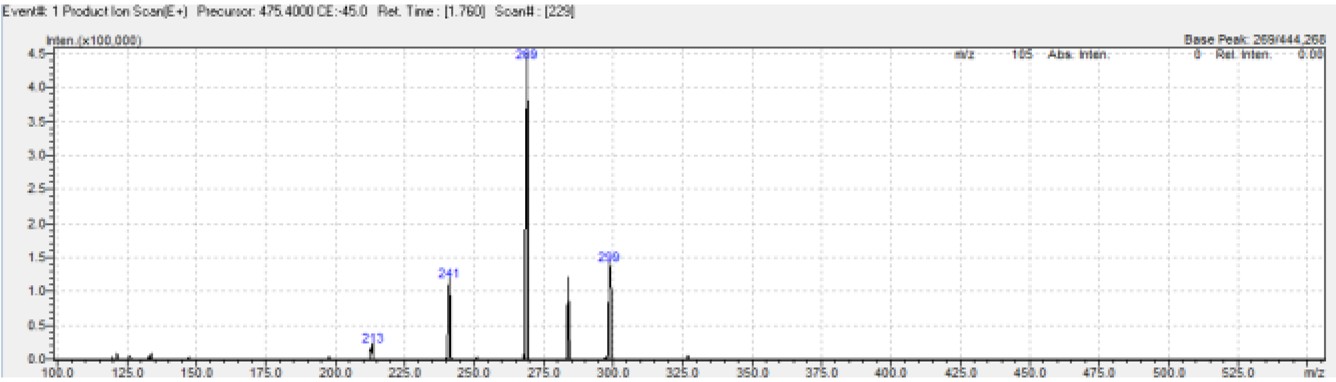

Fig.S70. $^{1}$H NMR spectral of 2’,5’-dimethoxyflavone 4’-*O*-β-D-(4”-*O*-methyl)-glucopyranoside (**16**) (DMSO-$d_6$, 600 MHz)

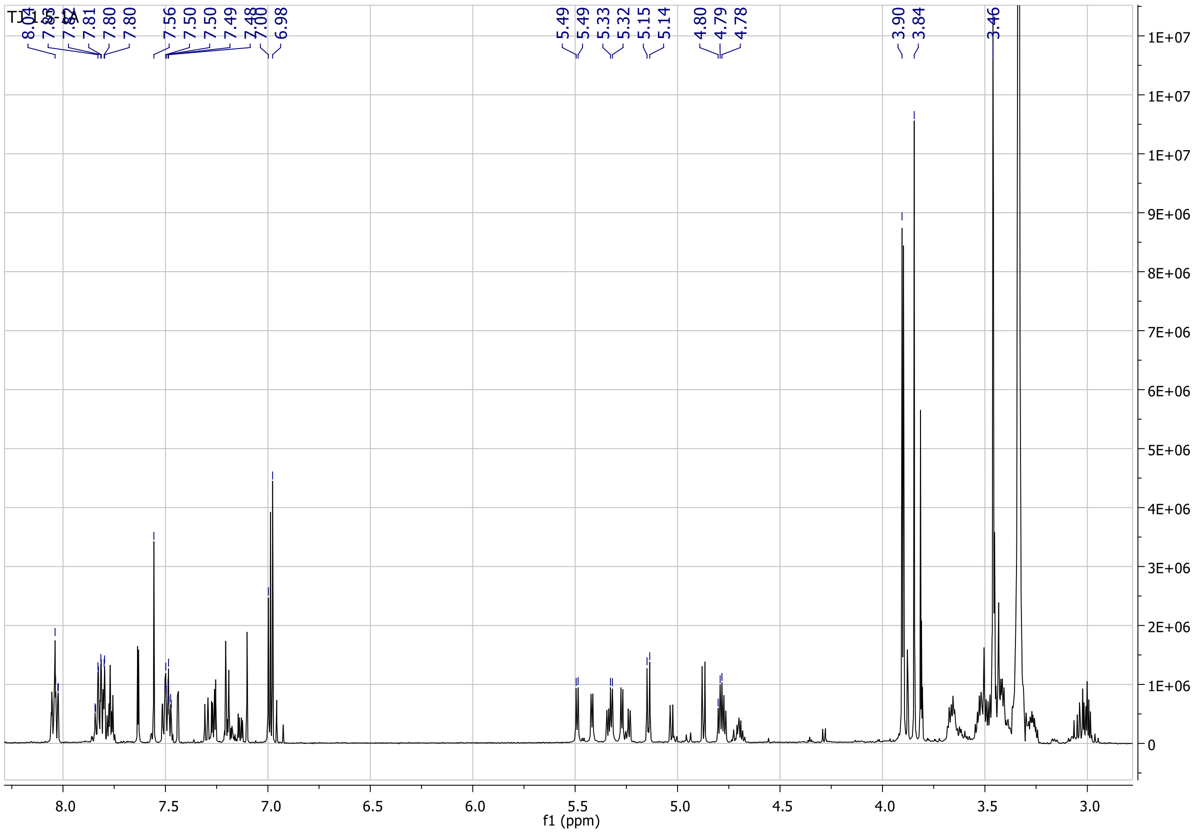


Fig.S71. Flavone part of the $^{1}$H NMR spectral 2’,5’-dimethoxyflavone 4’-*O*-β-D-(4”-*O*-methyl)-glucopyranoside (**16**) (DMSO-$d_6$, 600 MHz)

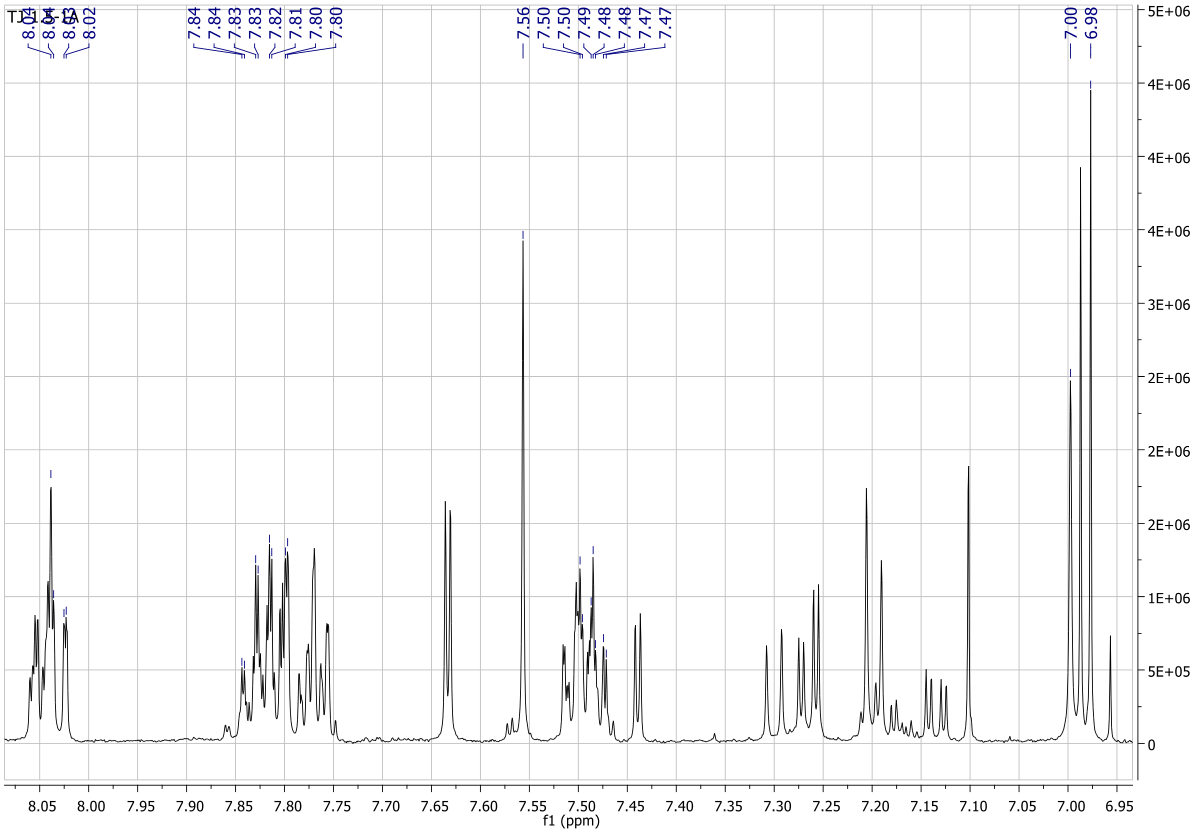

Fig.S72. Glucopyranoside part of the $^{1}$H NMR spectral 2',5'-dimethoxyflavone 4'-*O*-β-D-(4''-*O*-methyl)-glucopyranoside (**16**) (DMSO-$d_6$, 600 MHz)

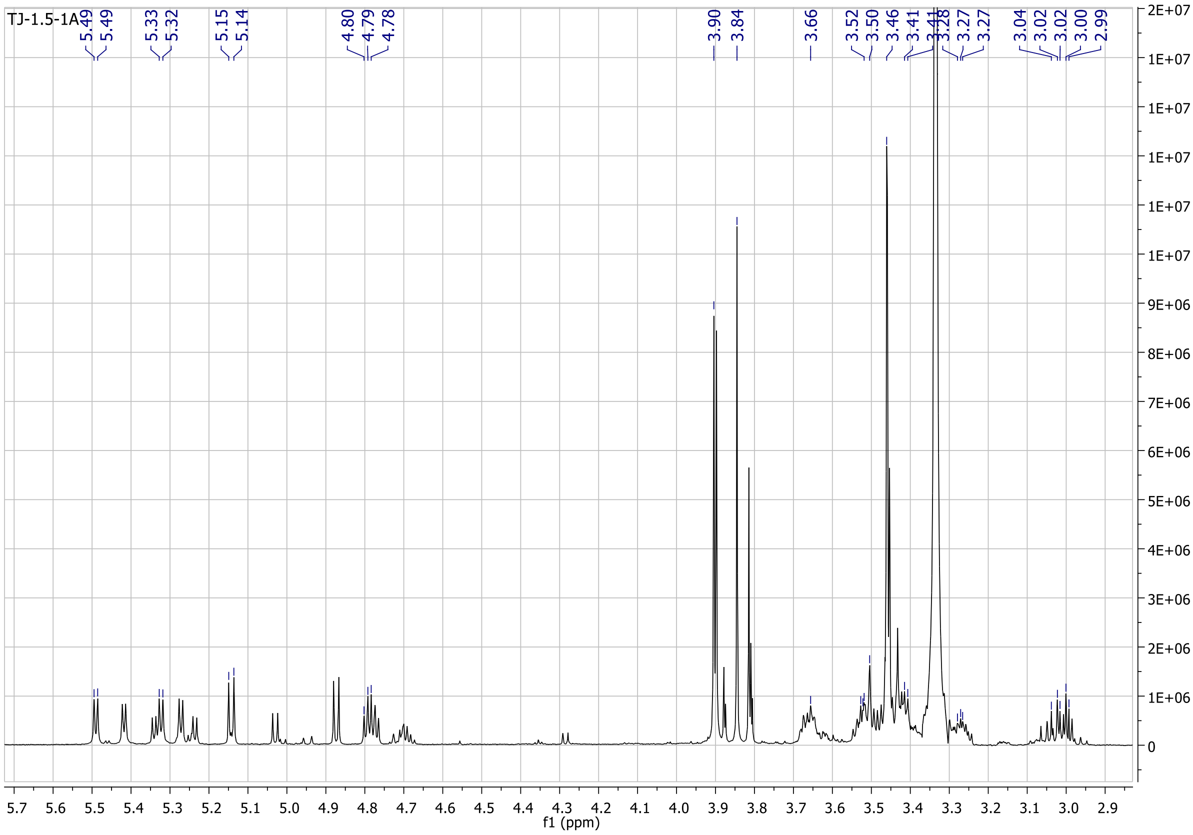


Fig.S73. $^{13}$C NMR spectral of 2',5'-dimethoxyflavone 4'-*O*-β-D-(4''-*O*-methyl)-glucopyranoside (**16**) (DMSO-$d_6$, 151 MHz)

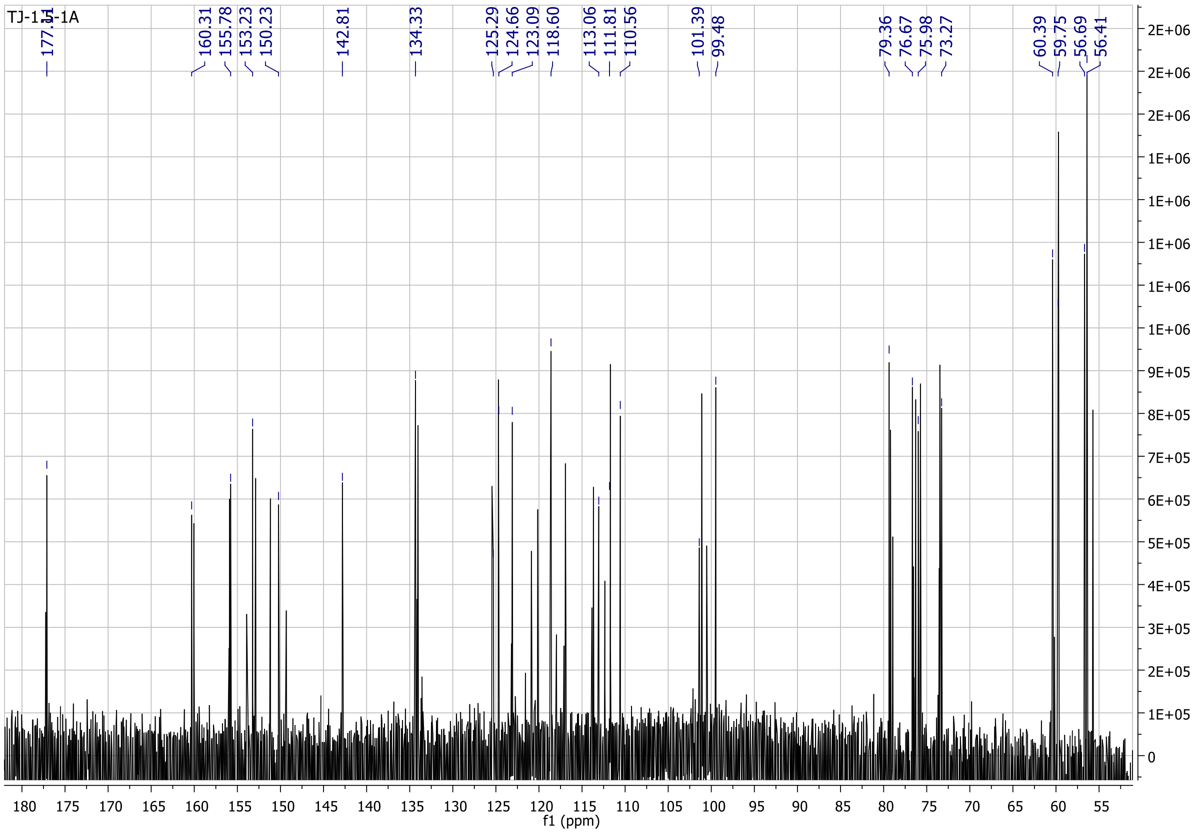

Fig.S74. COSY spectral of 2’,5’-dimethoxyflavone 4’-*O*-β-D-(4”-*O*-methyl)-glucopyranoside (**16**) (DMSO-$d_6$, 151 MHz)

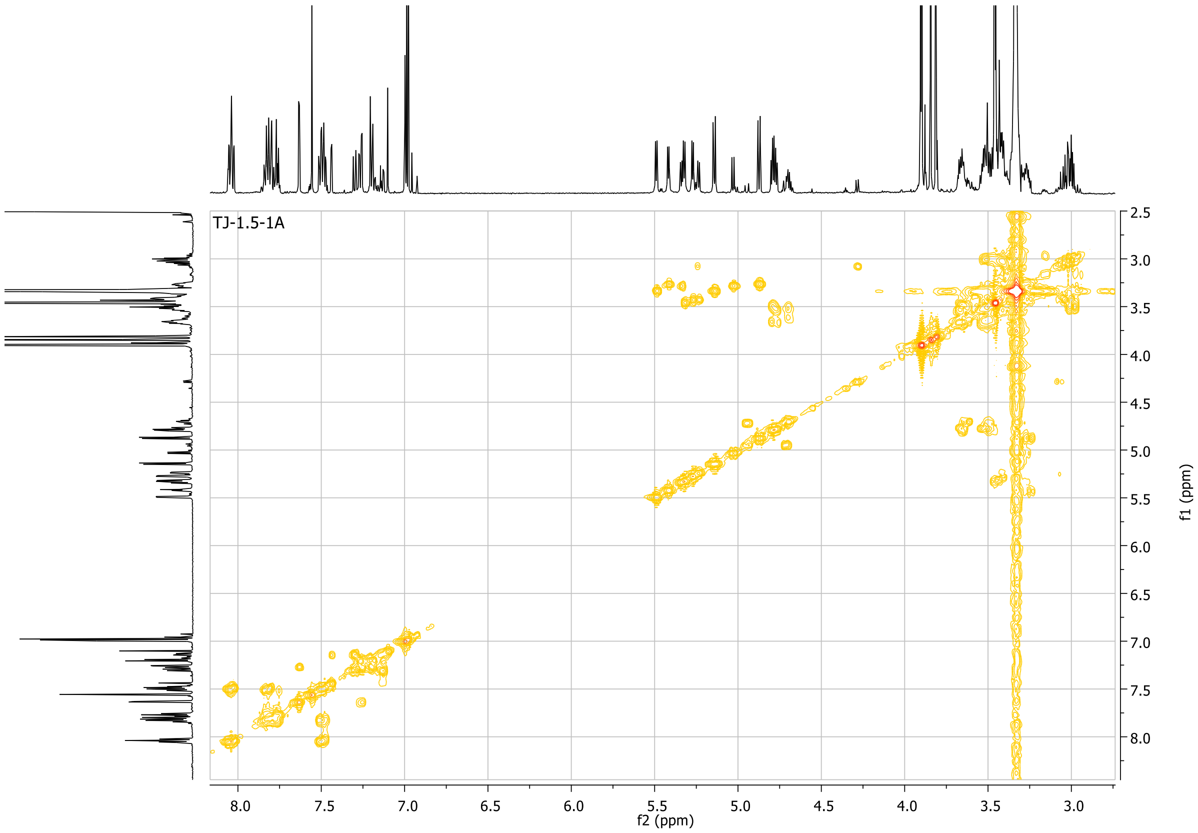


Fig.S75. HMQC spectral of 2’,5’-dimethoxyflavone 4’-*O*-β-D-(4”-*O*-methyl)-glucopyranoside (**16**) (DMSO-$d_6$, 151 MHz)

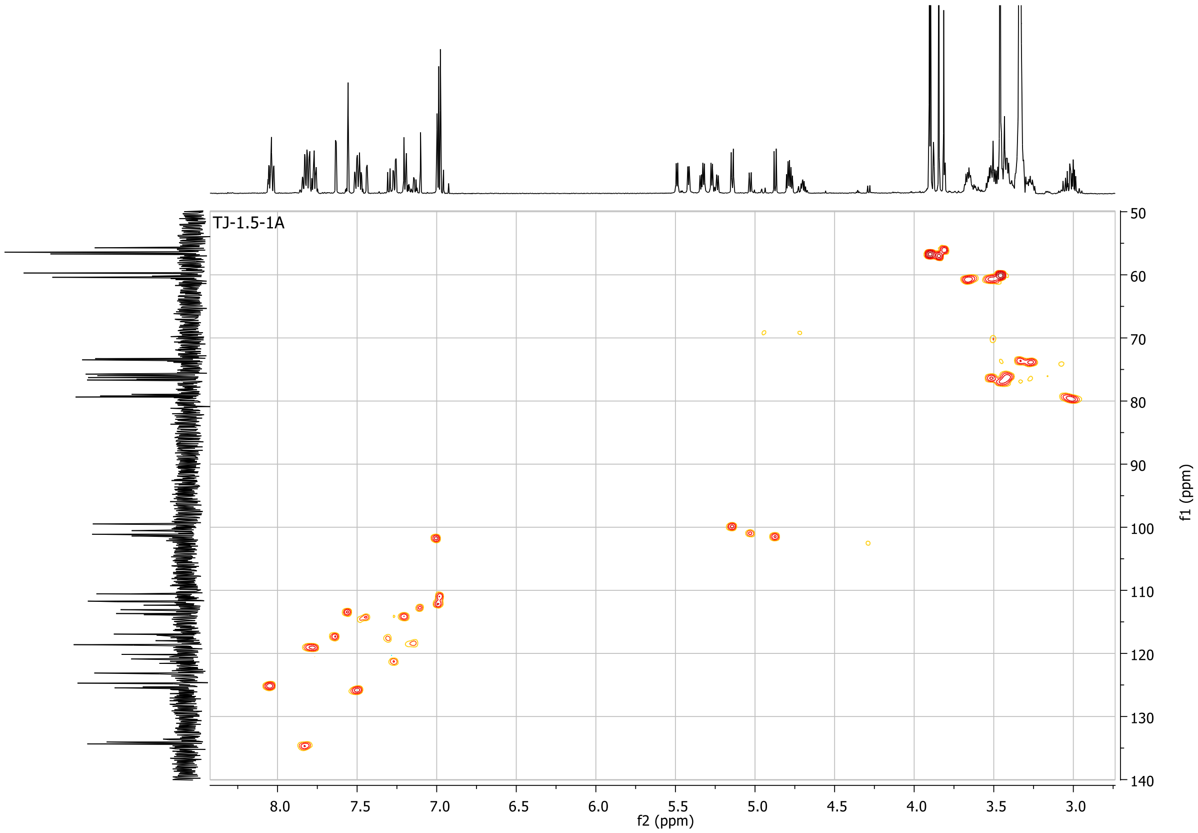

Fig.S76. HMBC spectral of 2',5'-dimethoxyflavone 4'-*O*-β-D-(4''-*O*-methyl)-glucopyranoside (**16**) (DMSO-$d_6$, 151 MHz)

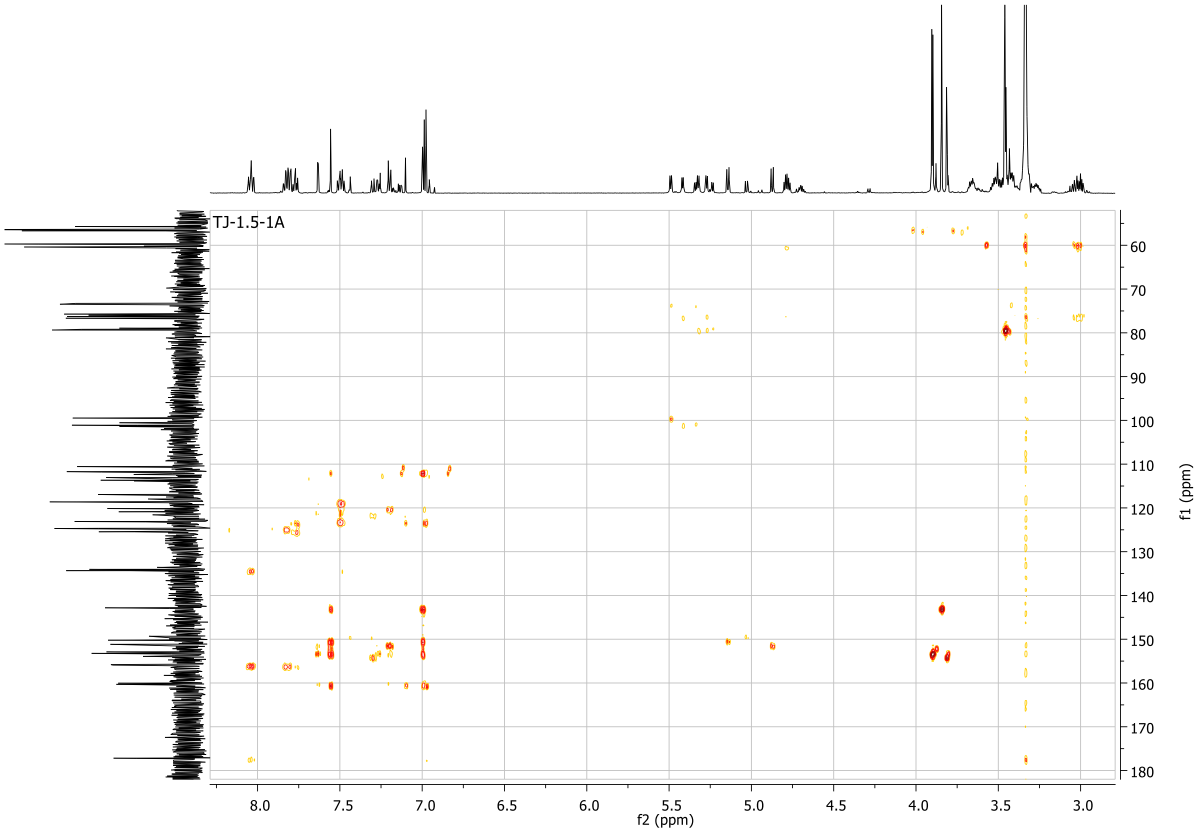

Fig.S77. MS analysis 5’-hydroxy-2’-methoxyflavone (**17**)

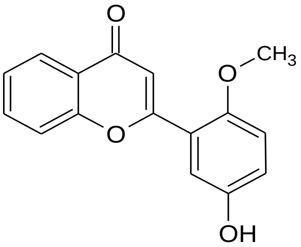


Molecular Formula: $C_{16}H_{12}O_4$
Formula Weight: 268.26408
Precursor: 269.2000

CE: -15.0

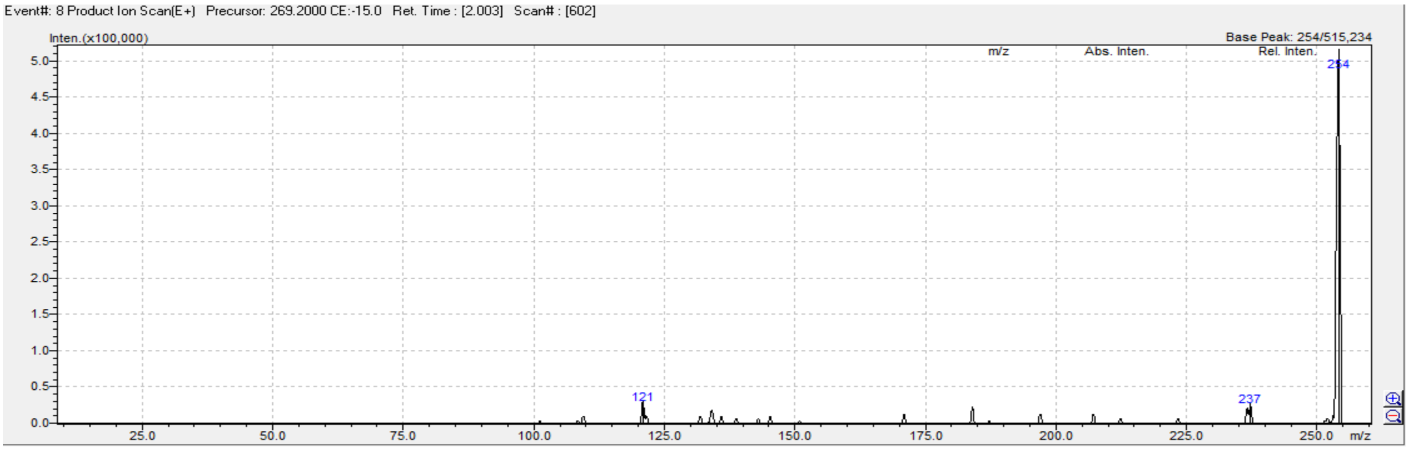


CE:-35.0

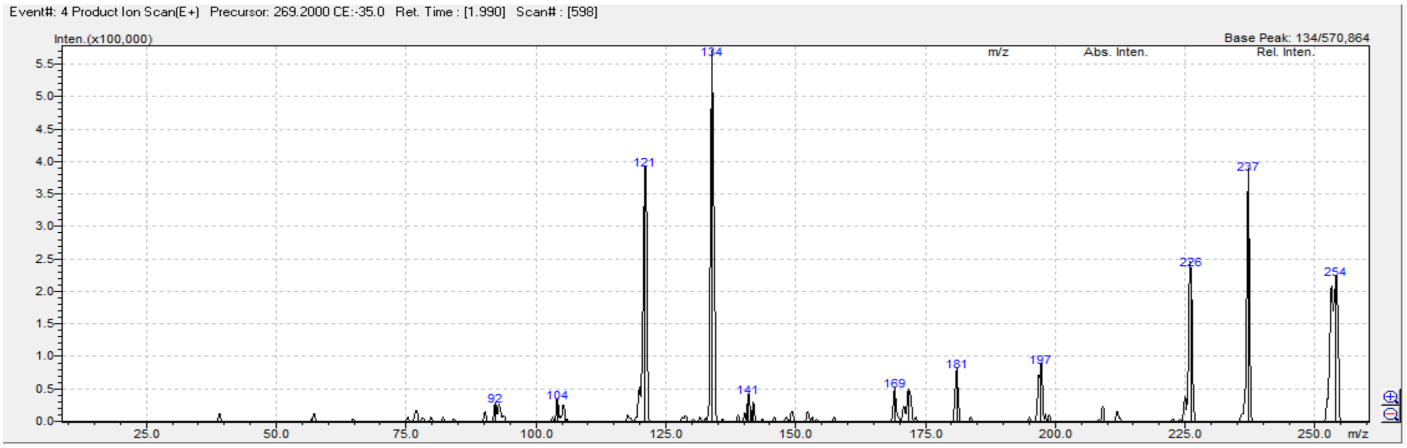


CE:-45.0

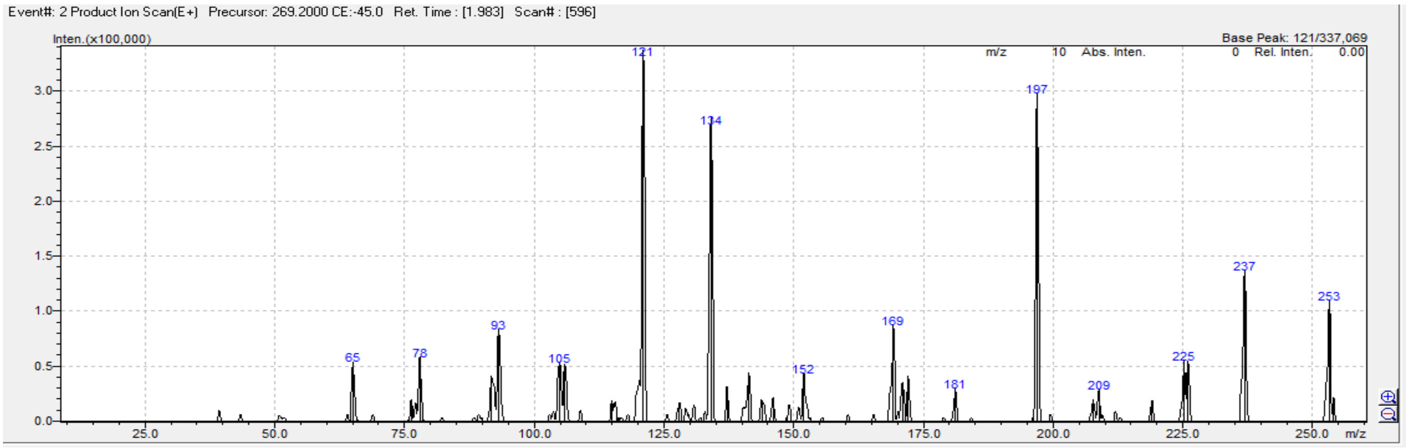

Fig.S78. $^{1}$H NMR spectral of 5’-hydroxy-2’-methoxyflavone (**17**) (DMSO-$d_6$, 600 MHz)

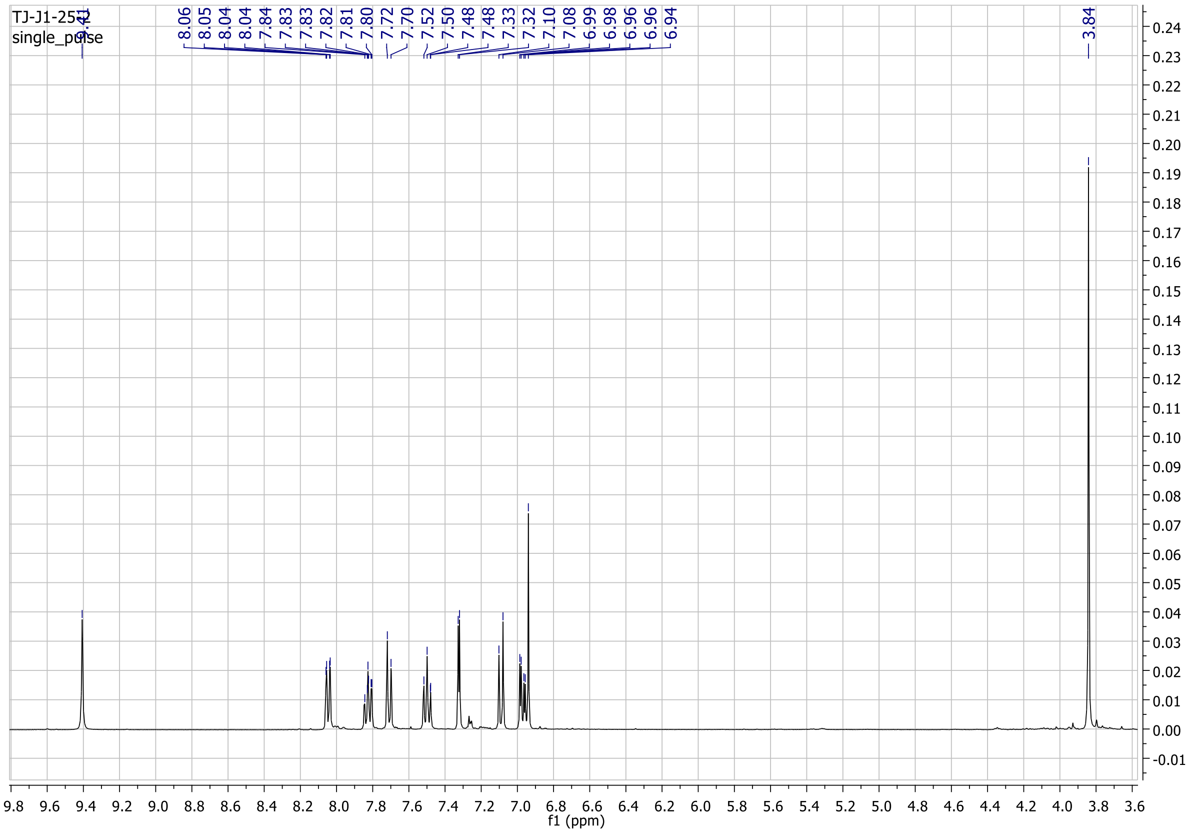


Fig.S79. $^{13}$C NMR spectral of 5’-hydroxy-2’-methoxyflavone (**17**) (DMSO-$d_6$, 151 MHz)

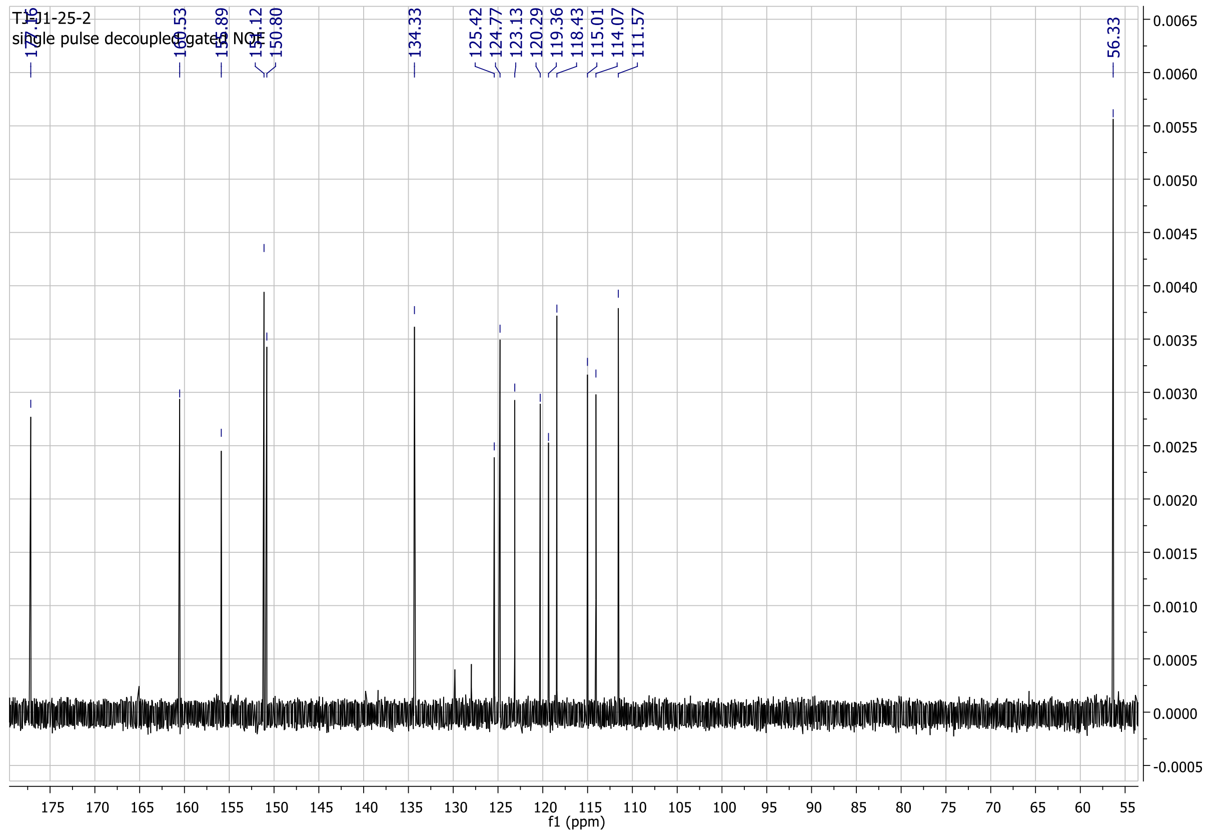

Fig.S80. COSY spectral of 5’-hydroxy-2’-methoxyflavone (**17**) (DMSO-$d_6$, 151 MHz)

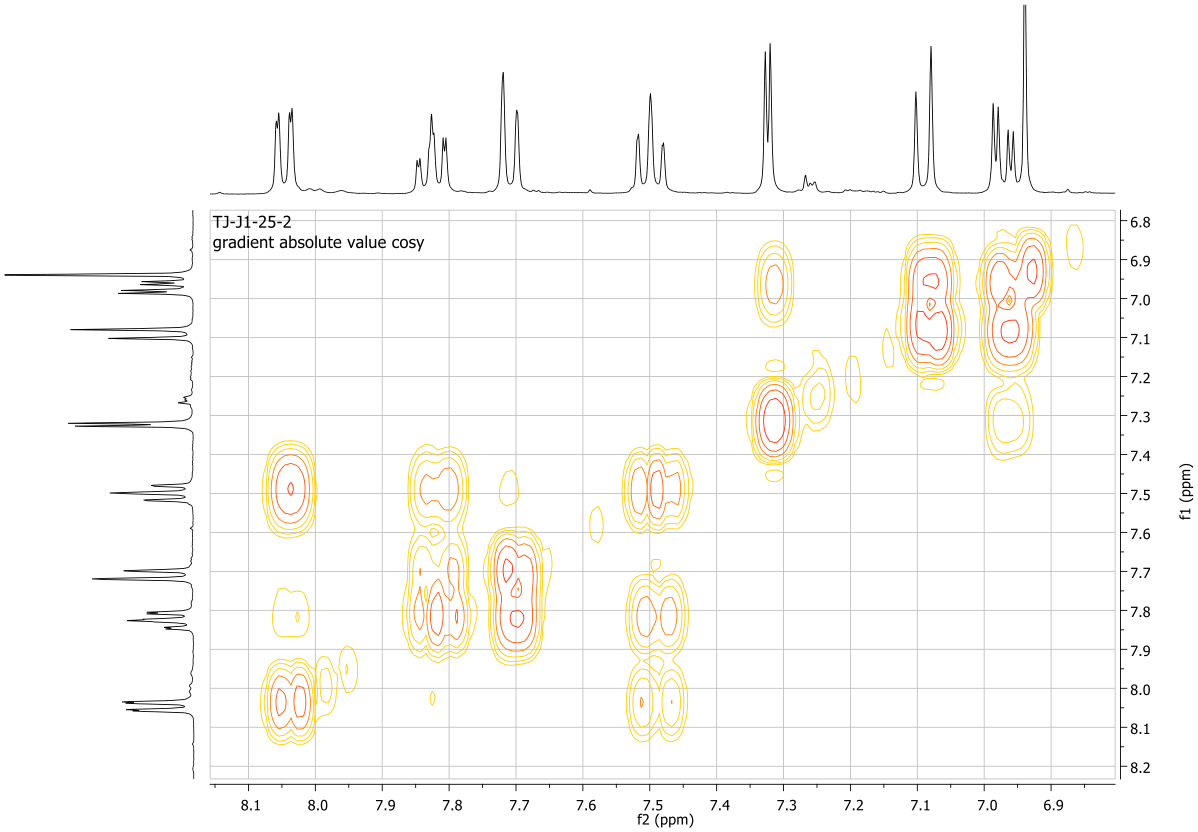


Fig.S81. HMQC spectral of 5’-hydroxy-2’-methoxyflavone (**17**) (DMSO-$d_6$, 151 MHz)

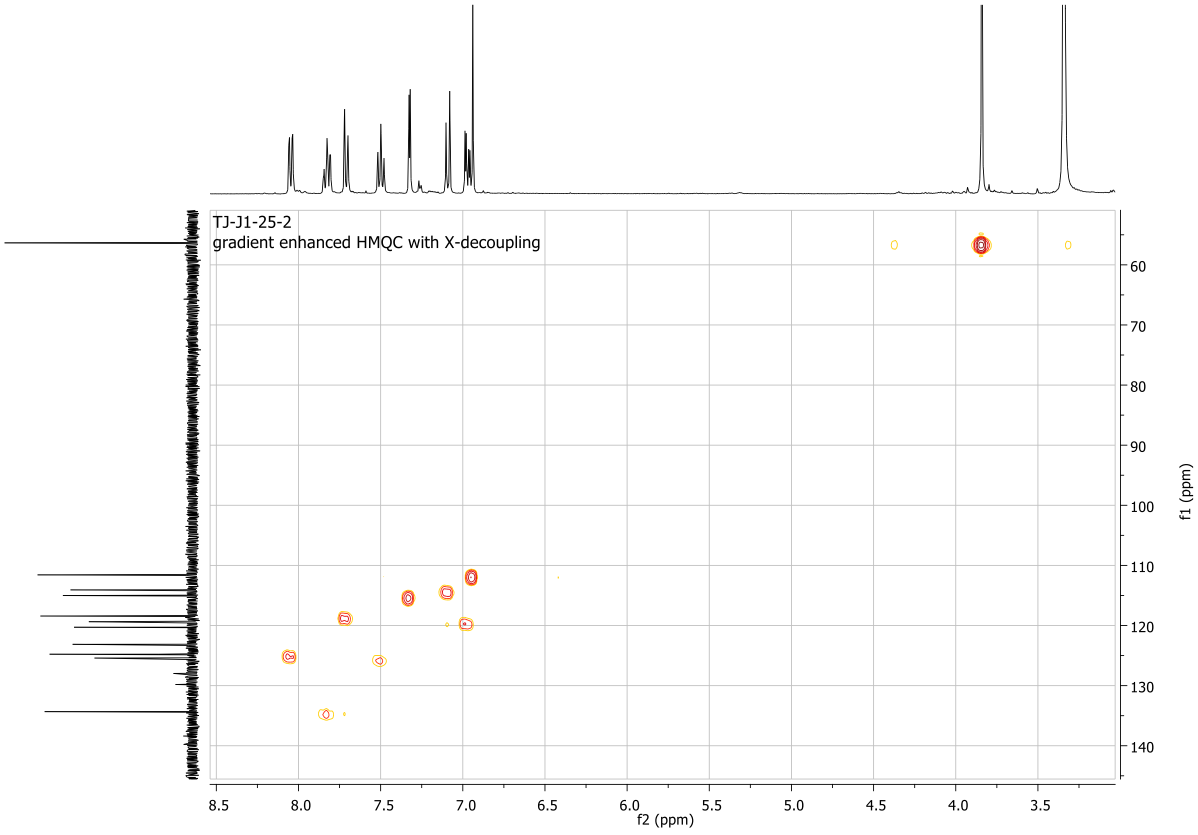

Fig.S82. HMBC spectral of 5'-hydroxy-2'-methoxyflavone (**17**) (DMSO-$d_6$, 151 MHz)

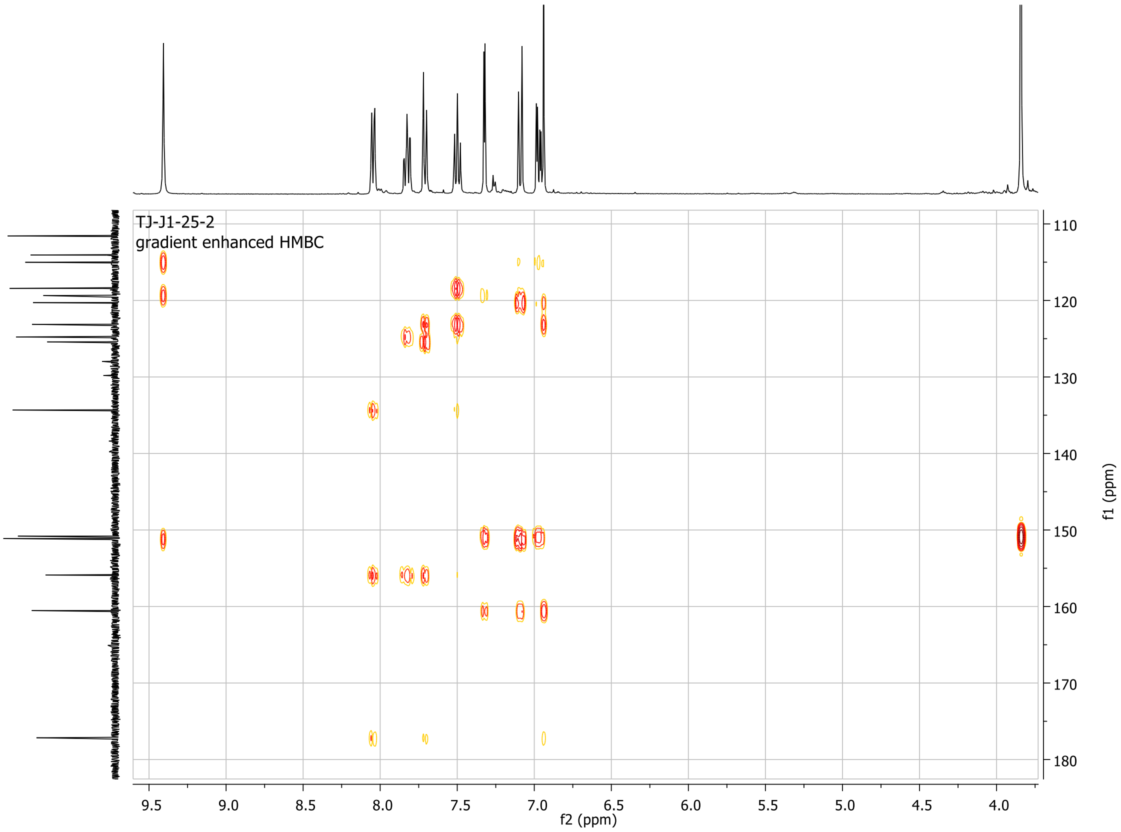

Fig.S83. MS analysis 4'-hydroxy-2',5'-dimethoxyflavone (**18**)

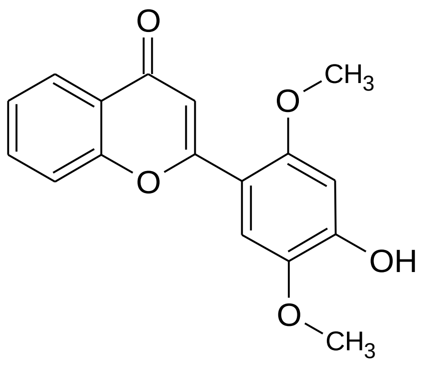

Molecular Formula = $C_{17}H_{14}O_5$
Formula Weight = 298.29006
Precursor: = 299.2000

CE: -15

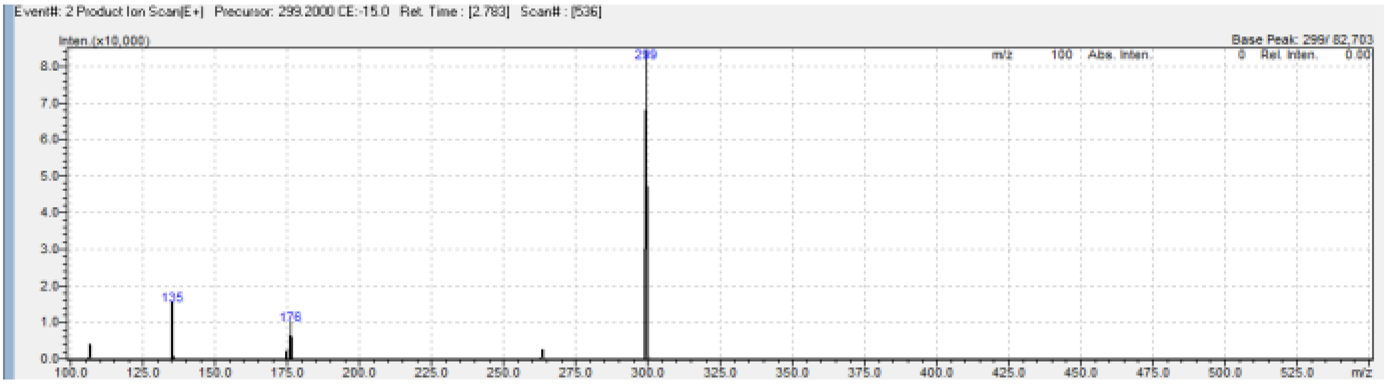


CE:-35

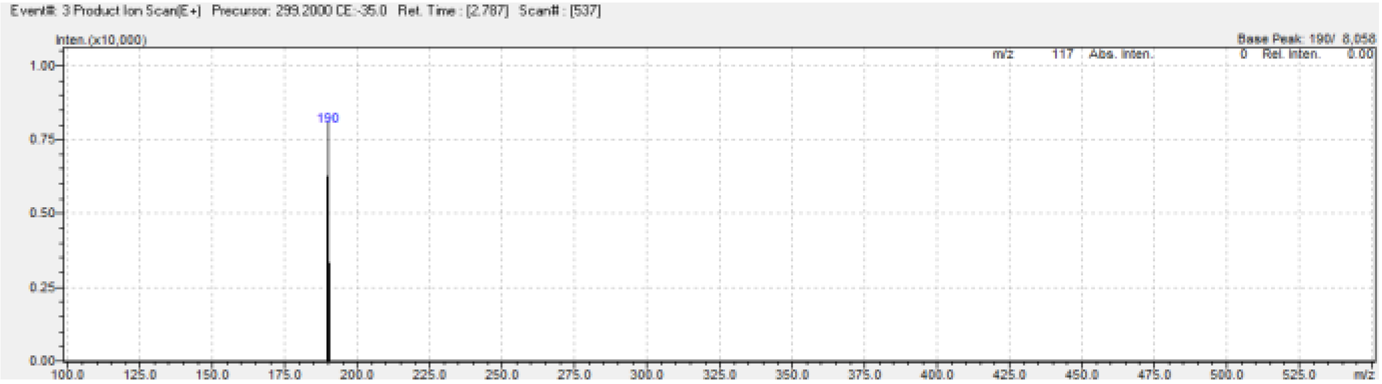


CE:-45

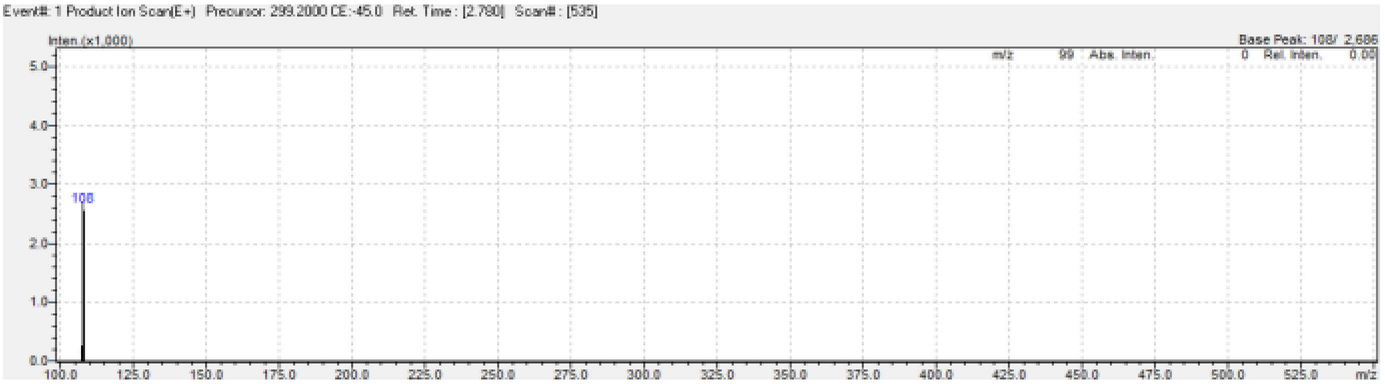

Fig.S84. $^{1}$H NMR spectral of 5’-hydroxy-2’-methoxyflavone (**17**) and 4’-hydroxy-2’,5’-dimethoxyflavone (**18**) (DMSO-$d_6$, 600 MHz)

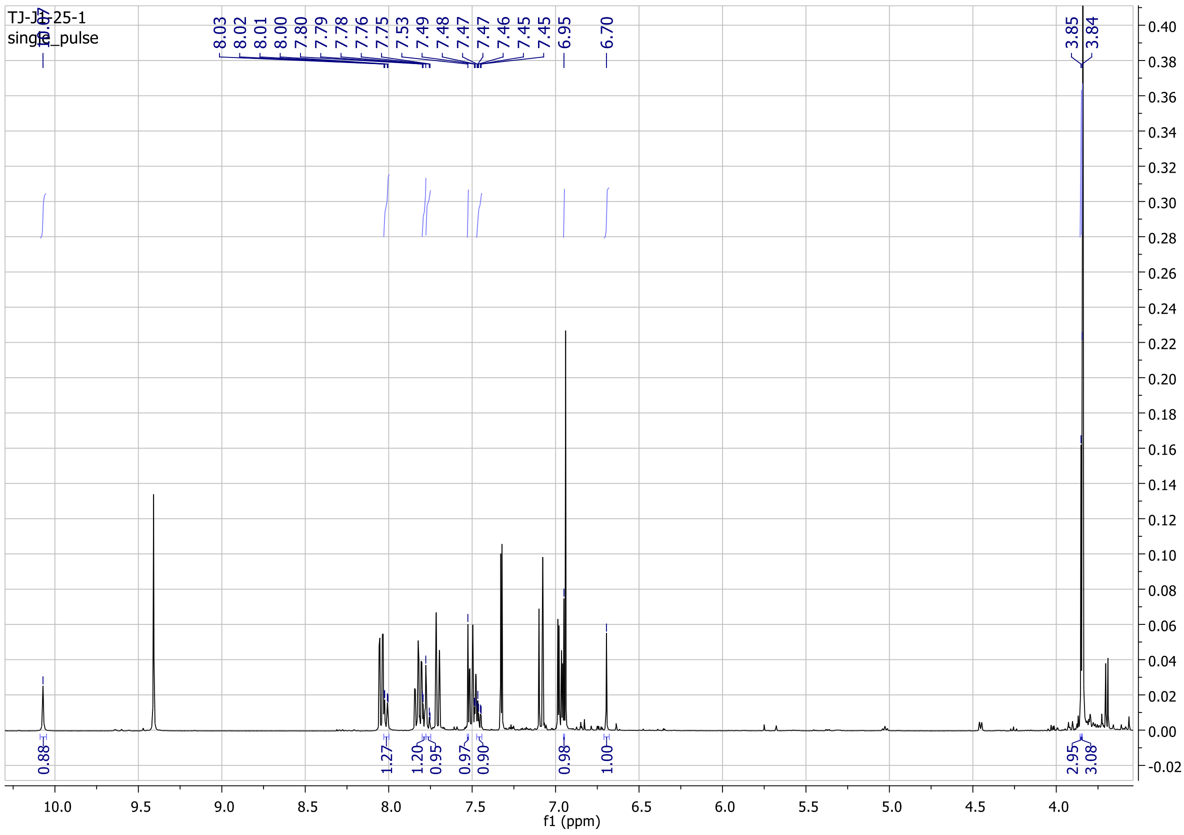


Fig.S85. Part of the $^{1}$H NMR spectral 5’-hydroxy-2’-methoxyflavone (**17**) and 4’-hydroxy-2’,5’-dimethoxyflavone (**18**) (DMSO-$d_6$, 600 MHz)

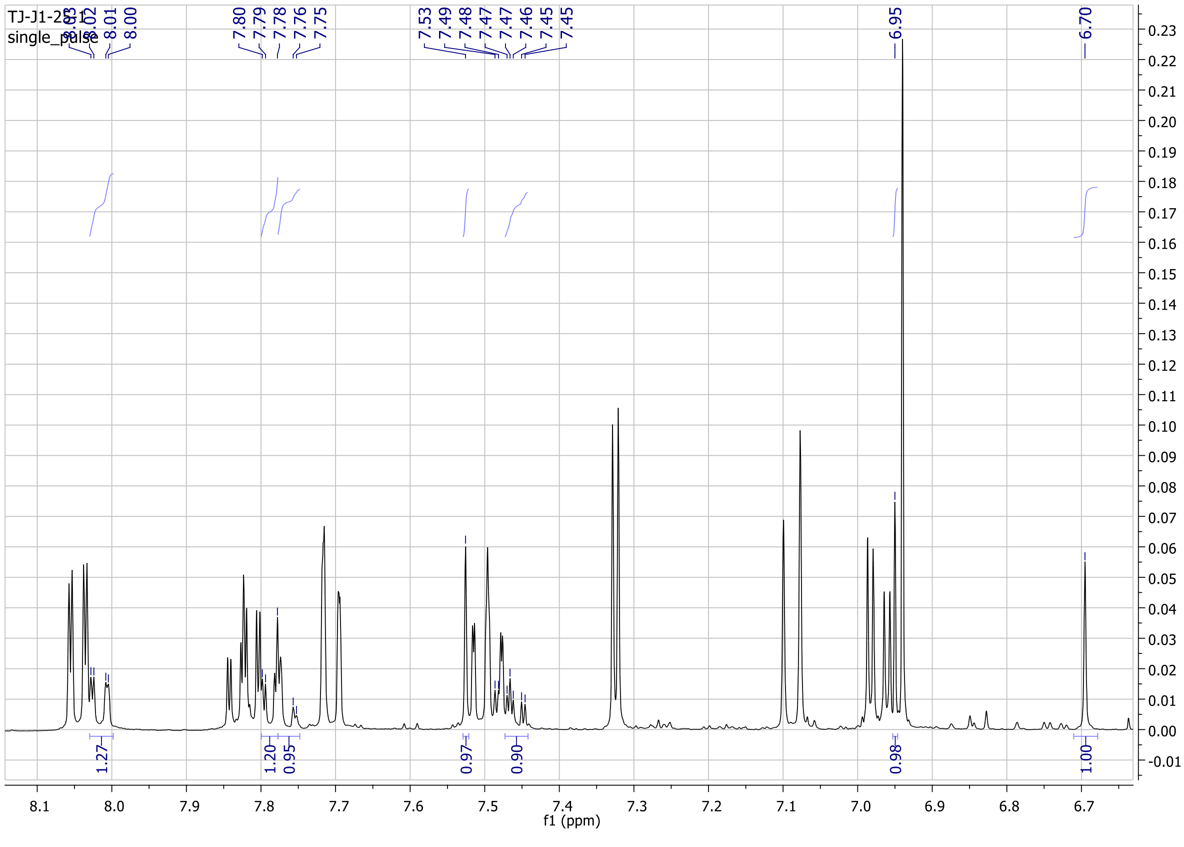

Fig.S86. $^{13}$C NMR spectral of 5'-hydroxy-2'-methoxyflavone (**18**) and 4'-hydroxy-2',5'-dimethoxyflavone (**18**) (DMSO-$d_6$, 151 MHz)

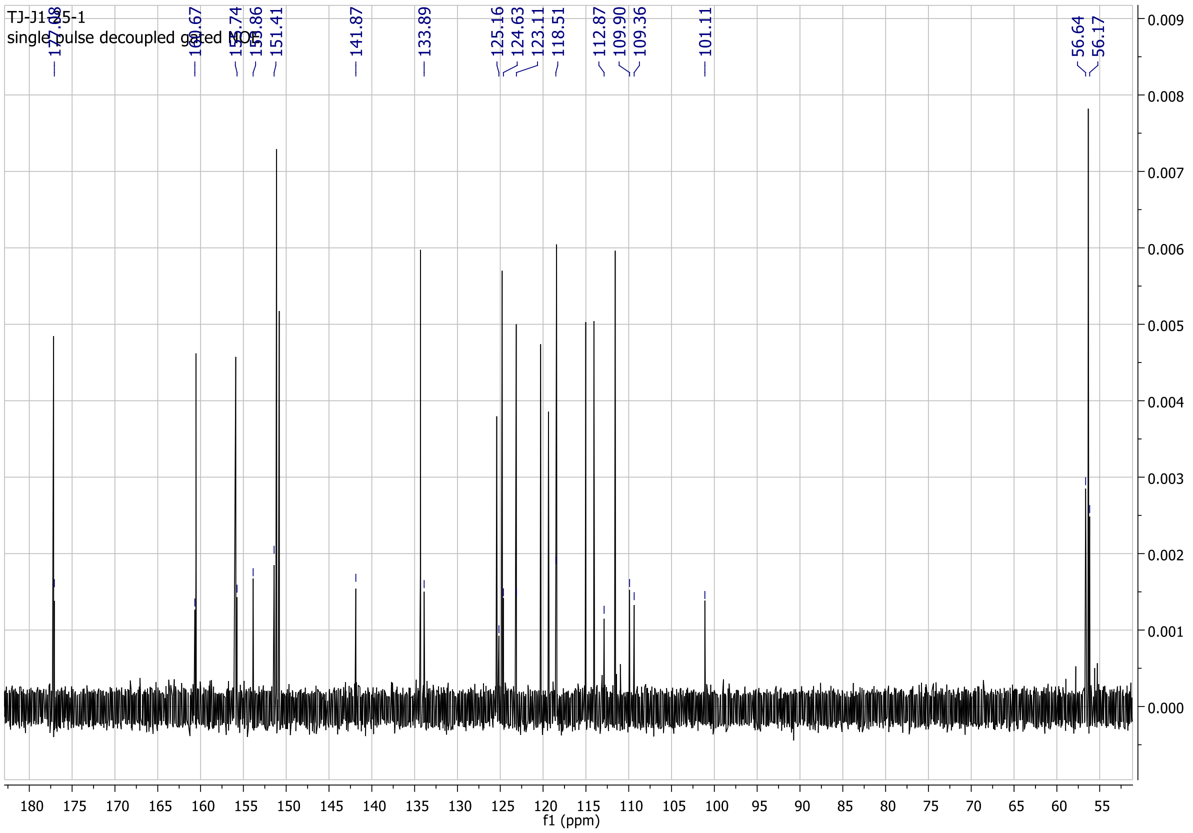


Fig.S87. HMQC spectral of 5'-hydroxy-2'-methoxyflavone (**17**) and 4'-hydroxy-2',5'-dimethoxyflavone (**18**) (DMSO-$d_6$, 151 MHz)

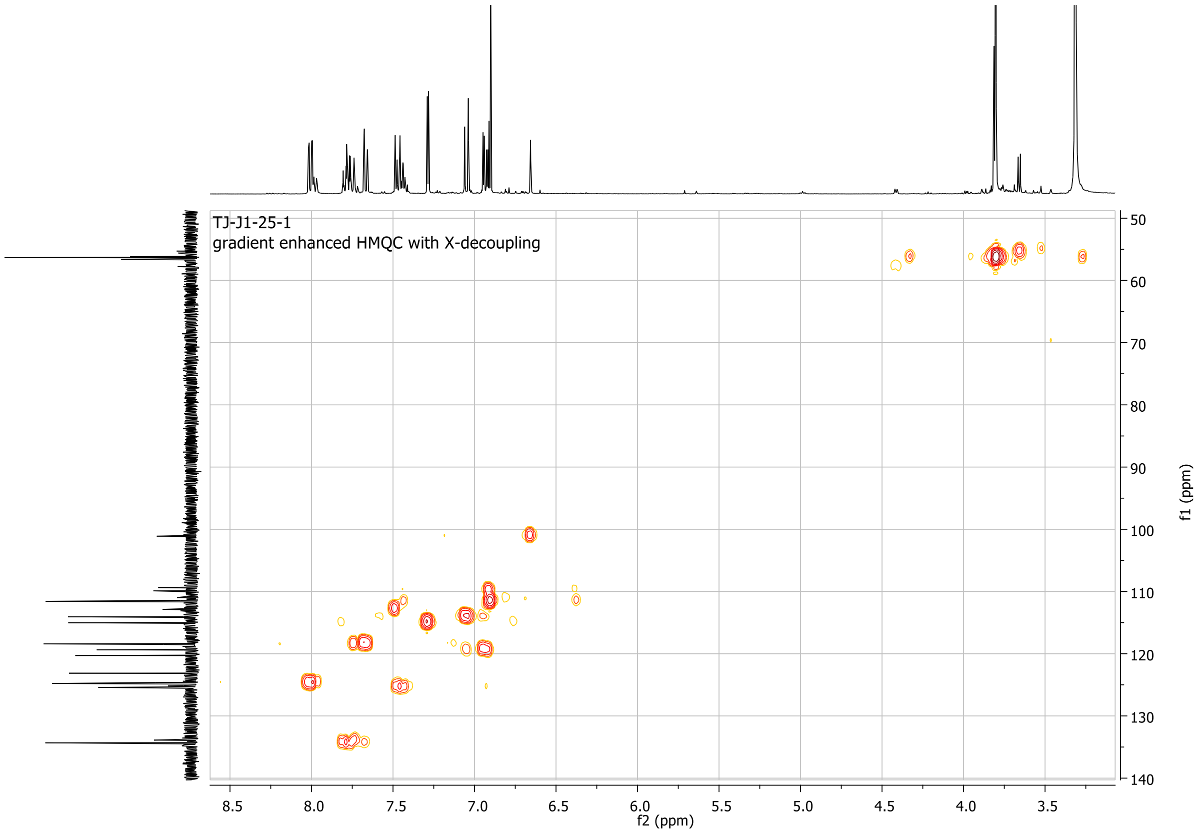

Fig.S88. HMBC spectral of 5’-hydroxy-2’-methoxyflavone (**17**) and 4’-hydroxy-2’,5’-dimethoxyflavone (**18**) (DMSO-$d_6$, 151 MHz)

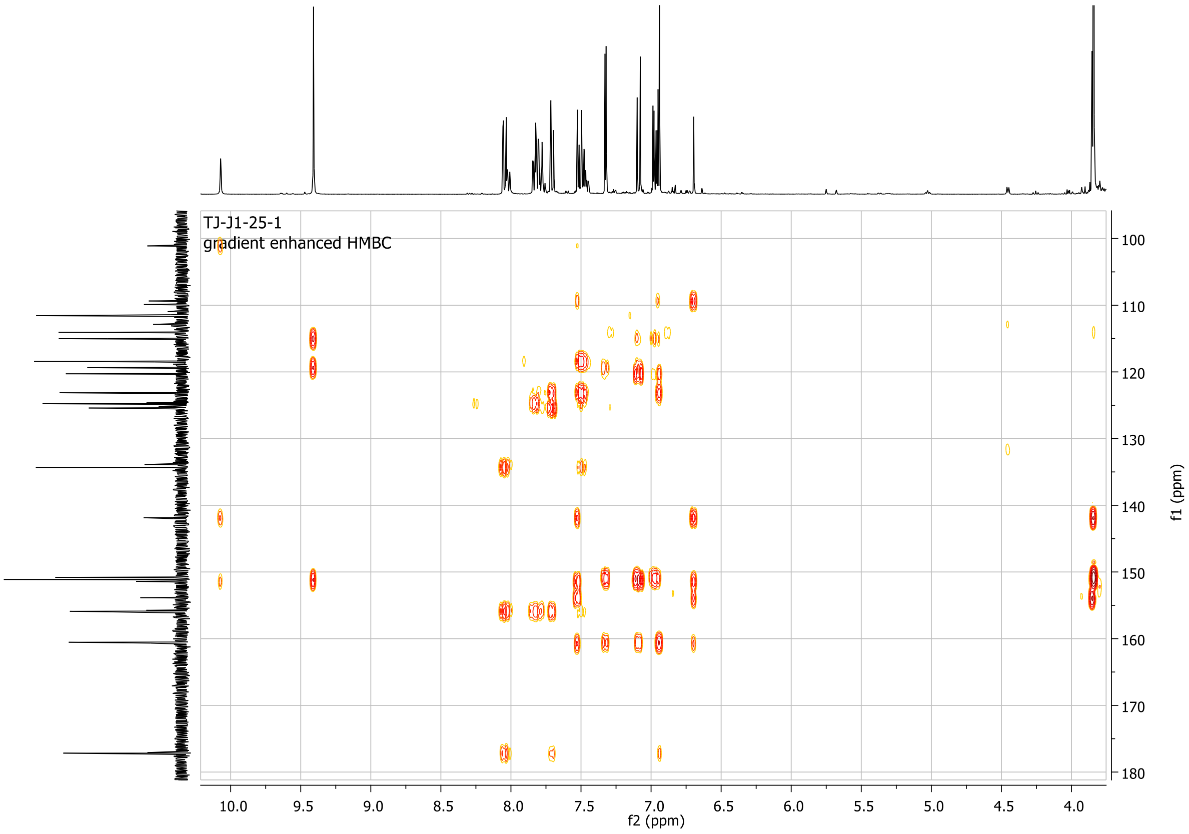

Fig.S89. MS analysis 4’,5’-dimethoxyflavone 5’-*O*-β-D-(4”-*O*-methyl)-glucopyranoside (**19**) and 3’,5’-dimethoxyflavone 4’-*O*-β-D-(4”-*O*-methyl)-glucopyranoside (**20**)

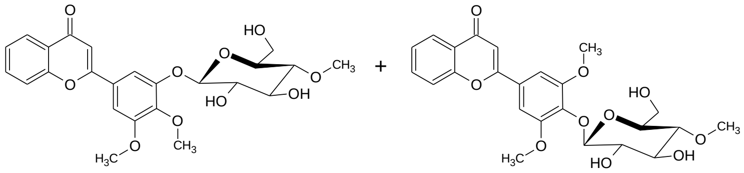

Molecular Formula = $C_{24}H_{26}O_{10}$
Formula Weight = 474.45724
Precursor =475.4000

CE: -15.0

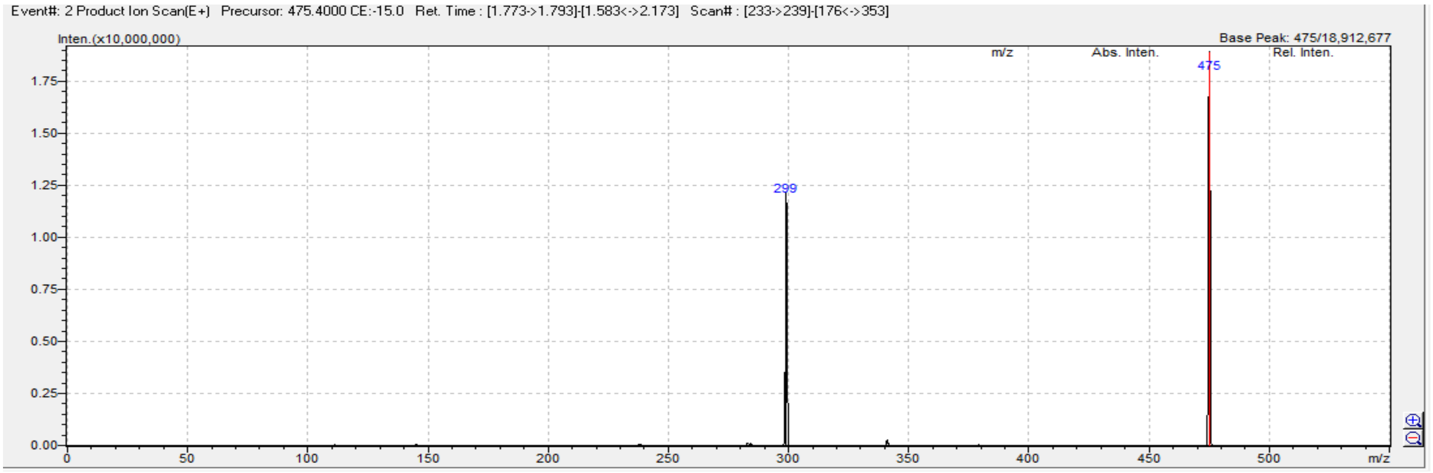


CE:-35.0

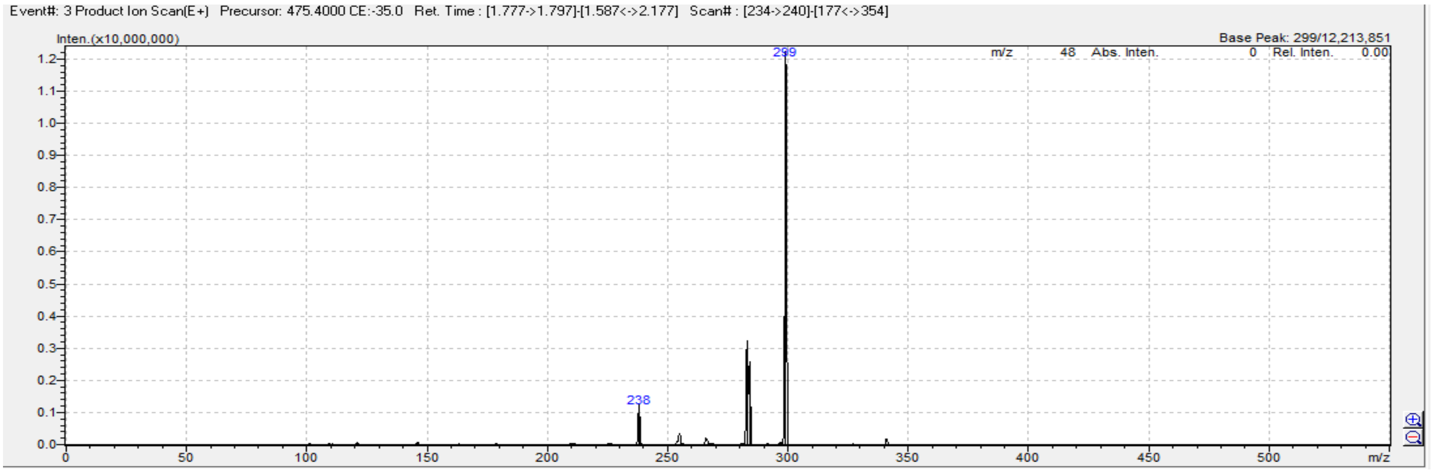


CE:-45.0

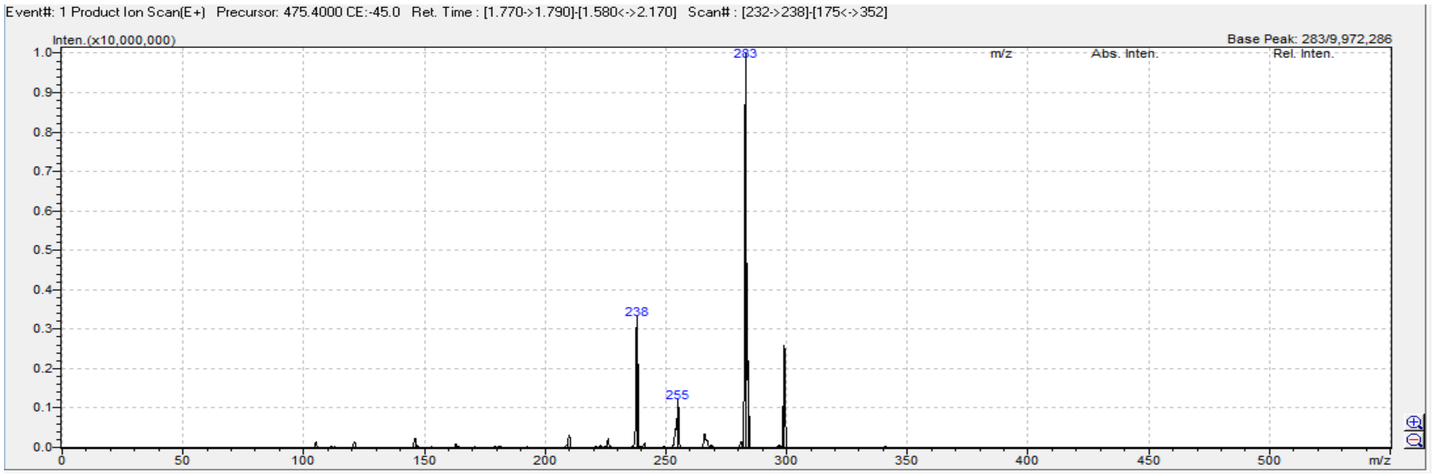

Fig.S90. $^{1}$H NMR spectral of 4’,5’-dimethoxyflavone 5’-*O*-β-D-(4”-*O*-methyl)-glucopyranoside (**19**) and 3’,5’-dimethoxyflavone 4’-*O*-β-D-(4”-*O*-methyl)-glucopyranoside (**20**) (DMSO-$d_6$, 600 MHz)

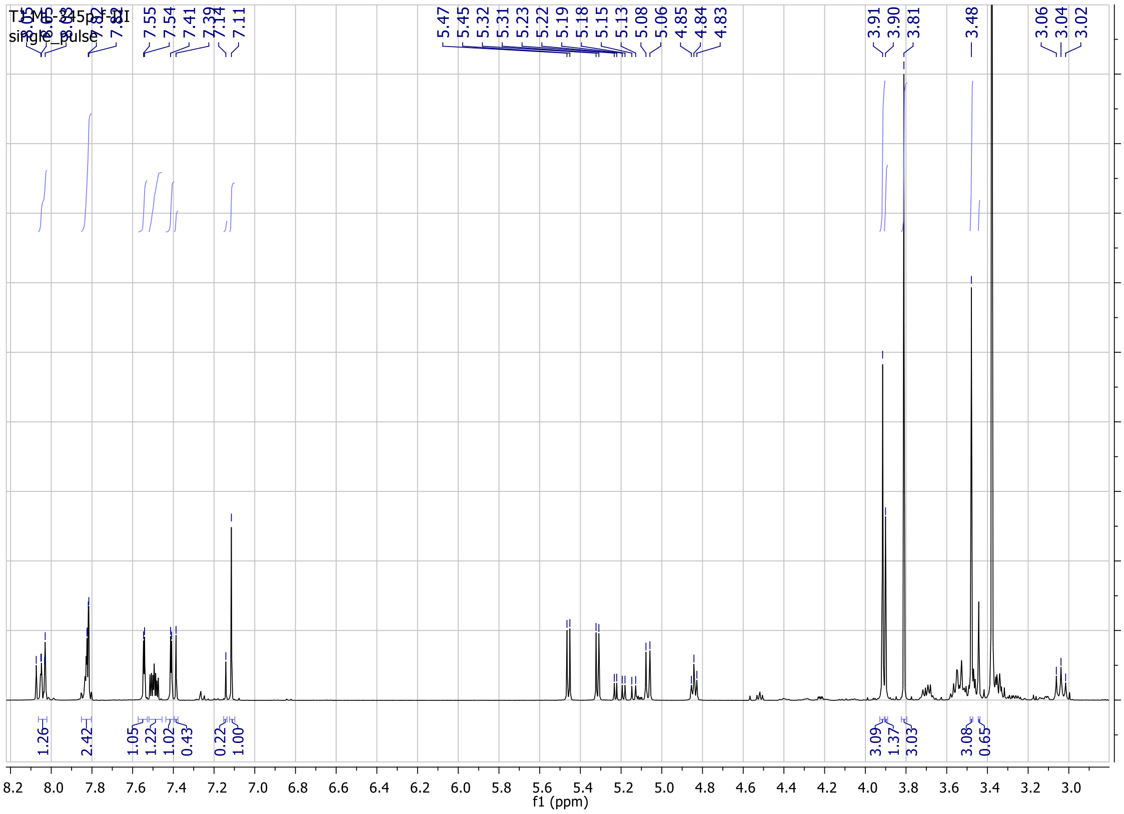

Fig.S91. Flavone part of the $^{1}$H NMR spectral 4’,5’-dimethoxyflavone 5’-*O*-β-D-(4”-*O*-methyl)-glucopyranoside (**19**) and 3’,5’-dimethoxyflavone 4’-*O*-β-D-(4”-*O*-methyl)-glucopyranoside (**20**) (DMSO-$d_6$, 600 MHz)

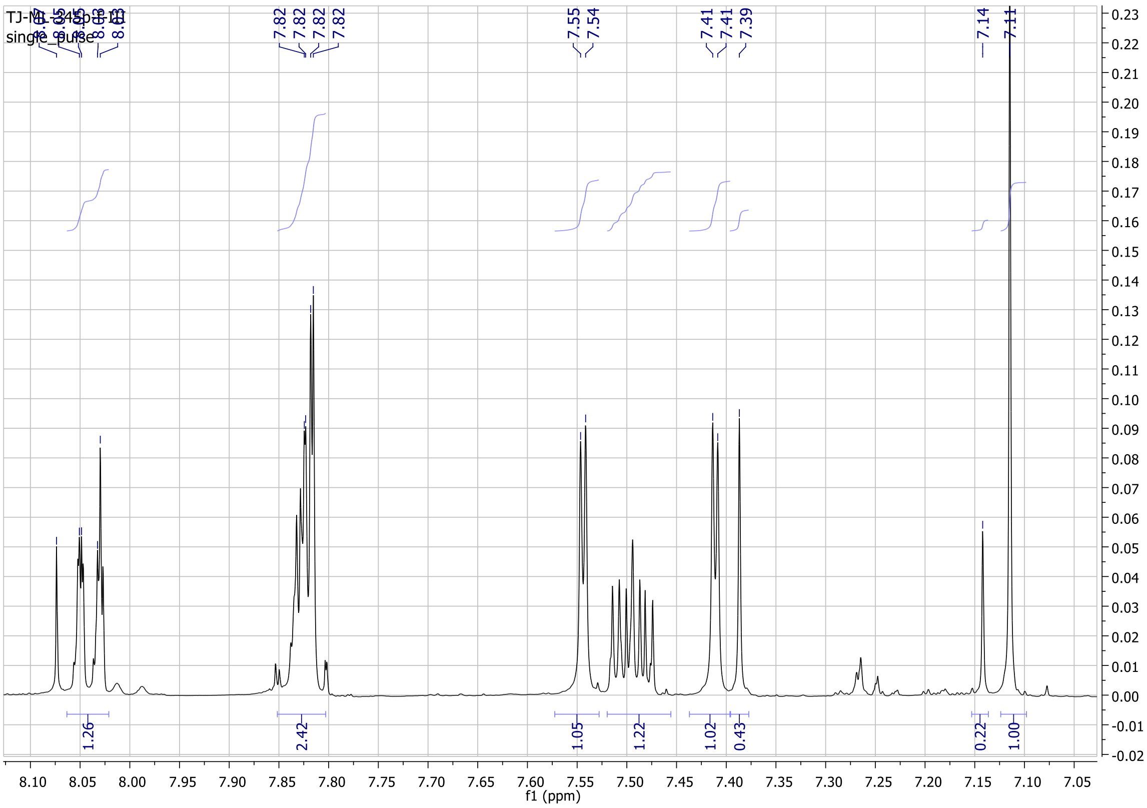

Fig.S92. Glycoside part of the $^{1}$H NMR spectral 4’,5’-dimethoxyflavone 5’-*O*-β-D-(4”-*O*-methyl)-glucopyranoside (**19**) and 3’,5’-dimethoxyflavone 4’-*O*-β-D-(4”-*O*-methyl)-glucopyranoside (**20**) (DMSO-$d_6$, 600 MHz)

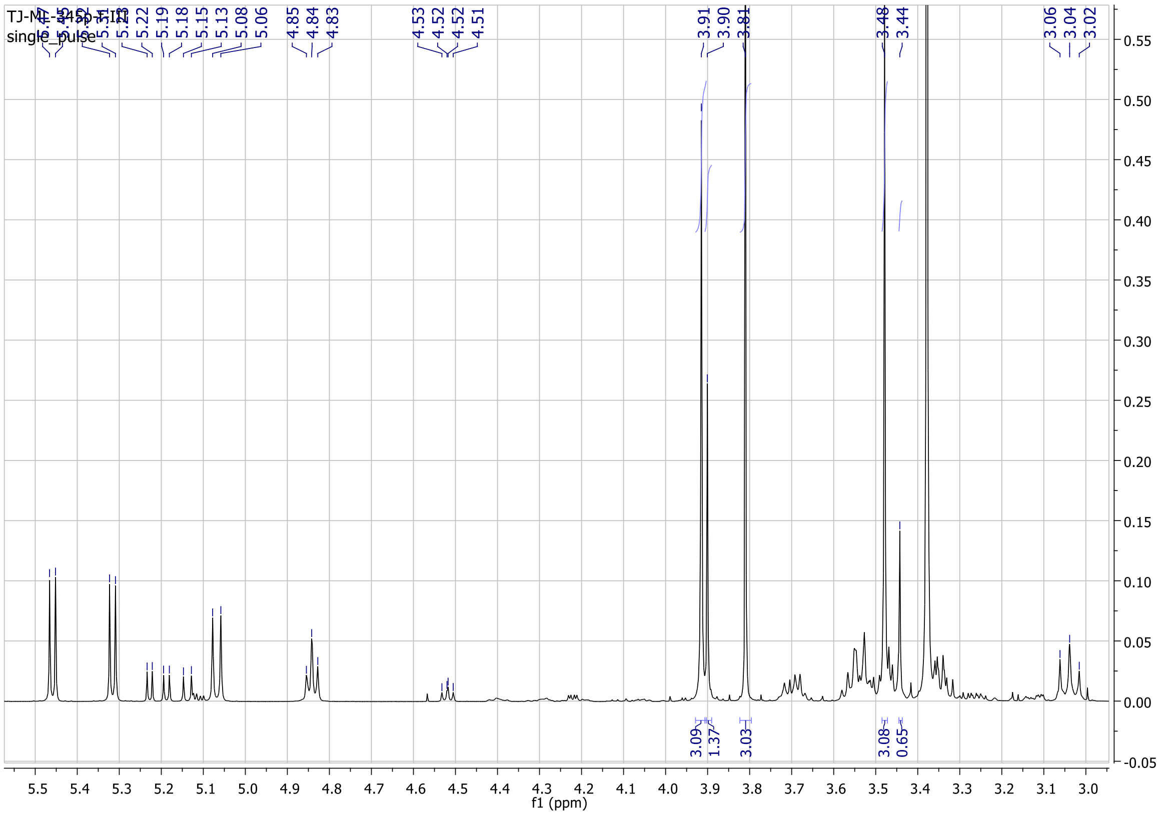


Fig.S93. $^{13}$C NMR spectral of 4’,5’-dimethoxyflavone 5’-*O*-β-D-(4”-*O*-methyl)-glucopyranoside (**19**) and 3’,5’-dimethoxyflavone 4’-*O*-β-D-(4”-*O*-methyl)-glucopyranoside (**20**) (DMSO-$d_6$, 151 MHz)

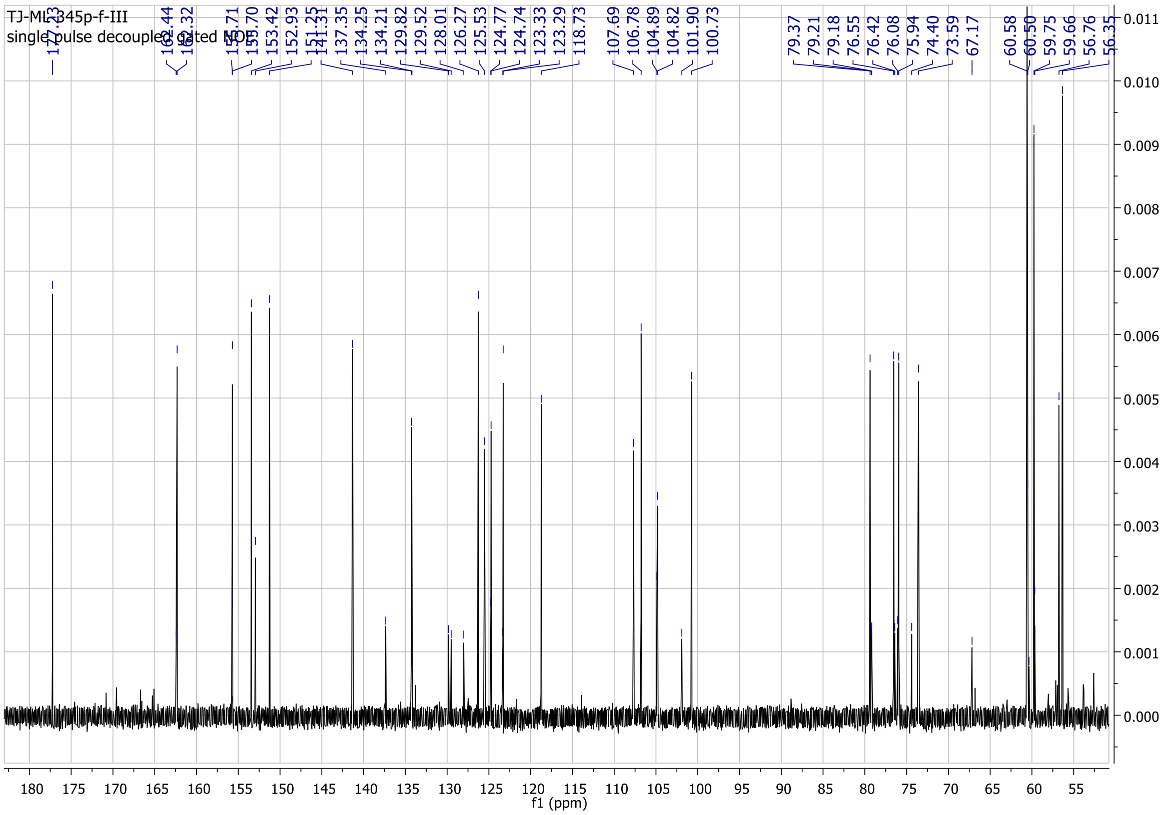

Fig.S94. HMQC spectral of 4’,5’-dimethoxyflavone 5’-*O*-β-D-(4”-*O*-methyl)-glucopyranoside (**19**) and 3’,5’-dimethoxyflavone 4’-*O*-β-D-(4”-*O*-methyl)-glucopyranoside (**20**) (DMSO-$d_6$, 151 MHz)

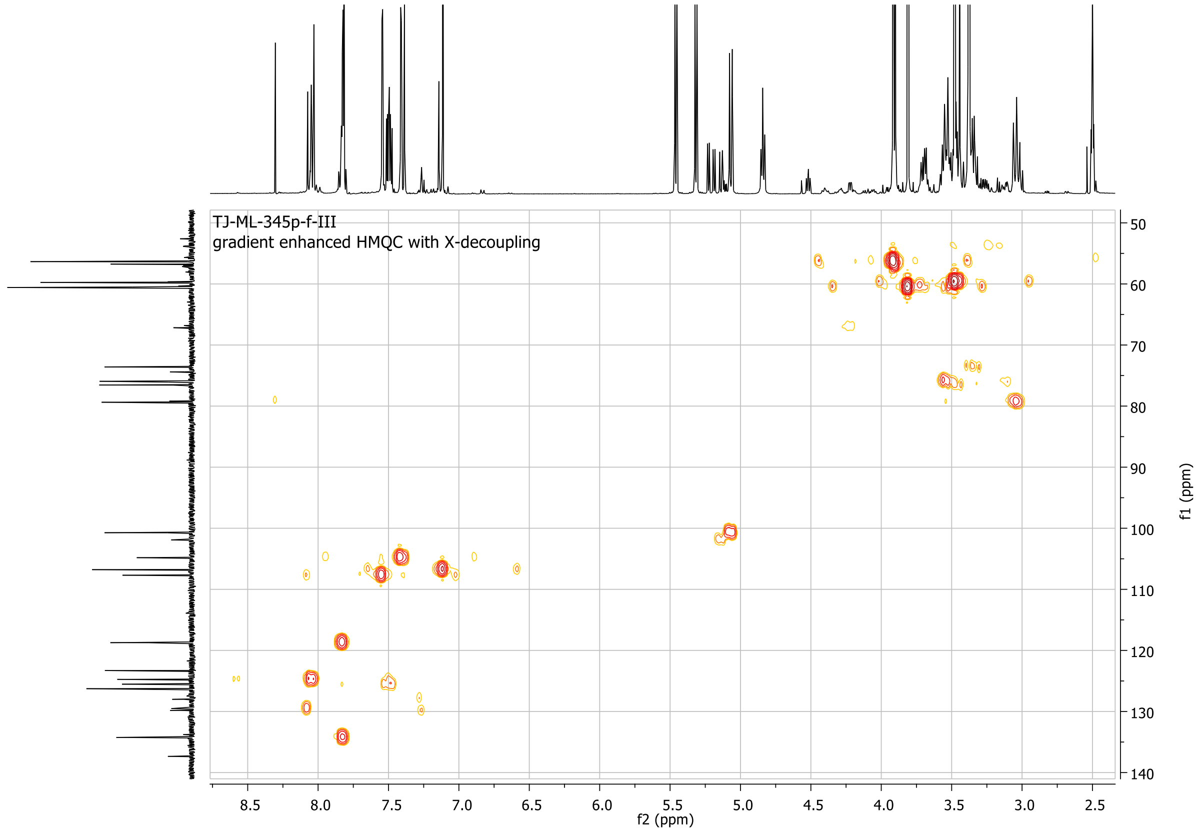


Fig.S95. HMBC spectral of 4’,5’-dimethoxyflavone 5’-*O*-β-D-(4”-*O*-methyl)-glucopyranoside (**19**) and 3’,5’-dimethoxyflavone 4’-*O*-β-D-(4”-*O*-methyl)-glucopyranoside (**20**) (DMSO-$d_6$, 151 MHz)

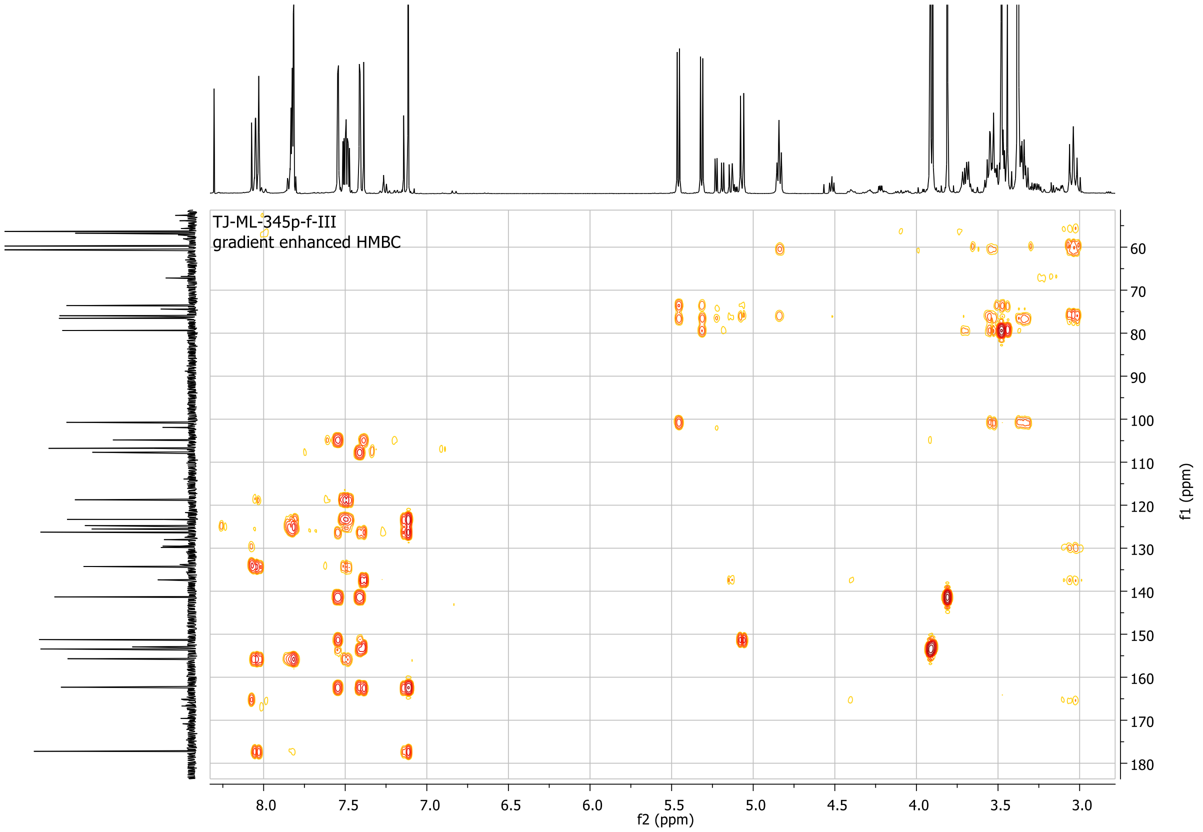

Fig.S96. MS analysis 3’,4’,5’-trimethoxyflavone 6-*O*-β-D-(4”-*O*-methyl)-glucopyranoside (**21**)

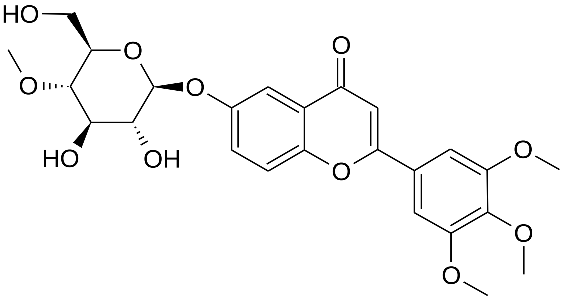

Molecular Formula = $C_{25}H_{28}O_{11}$
Formula Weight = 504.48322
Precursor: =505.4000

CE: -15.0

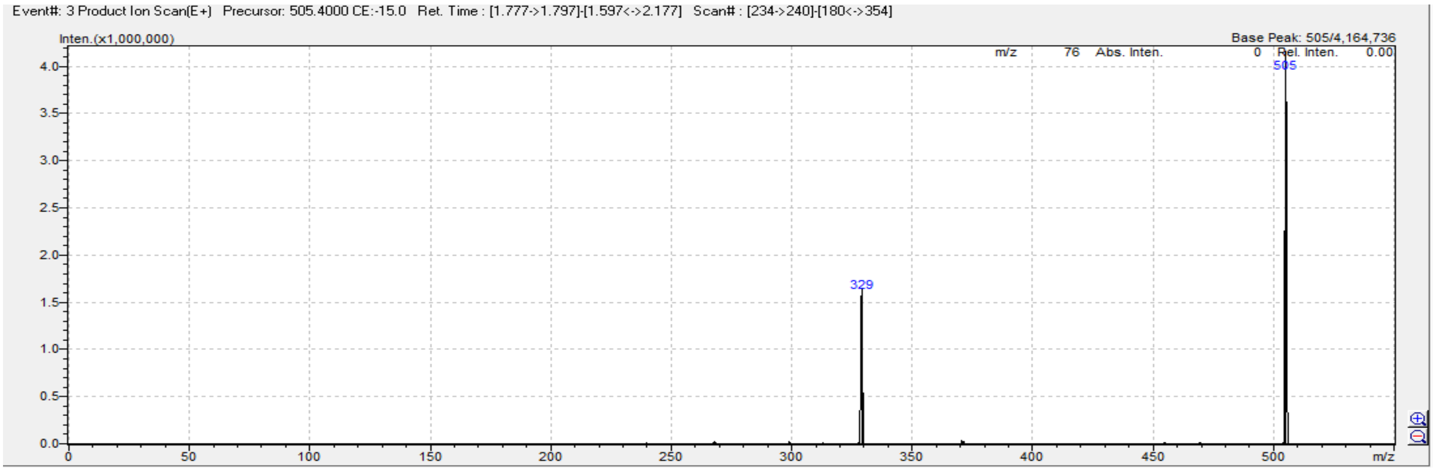


CE:-35.0

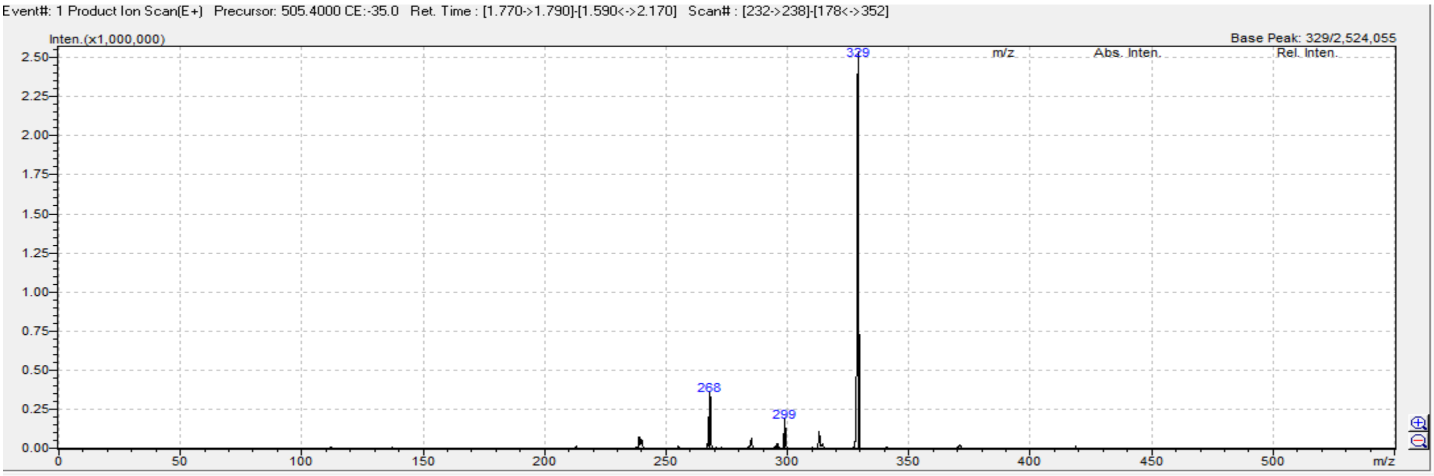


CE:-45.0

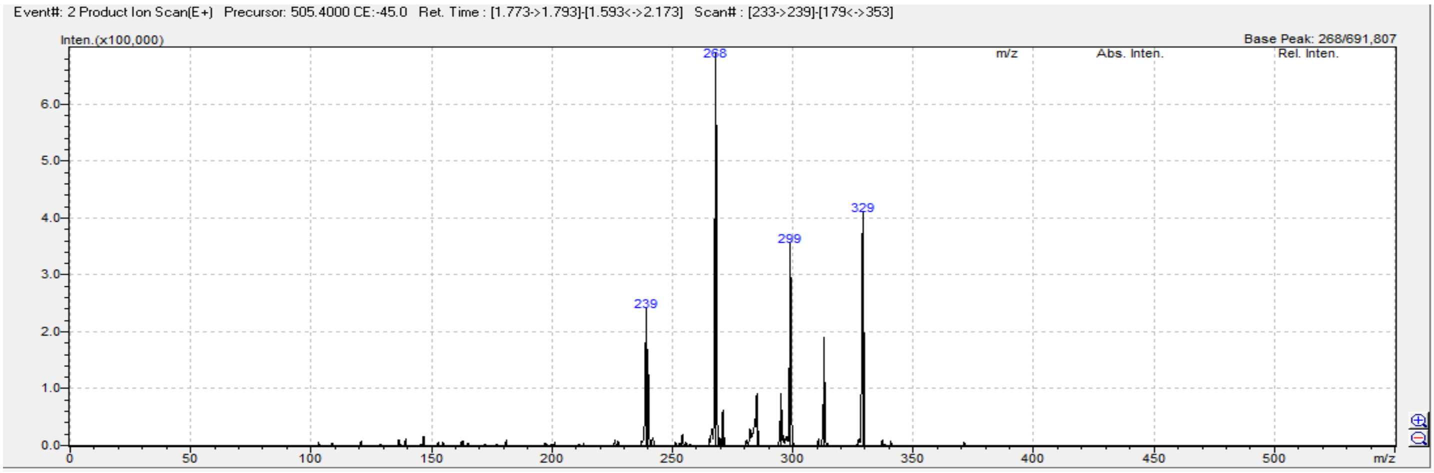

Fig.S97. $^{1}$H NMR spectral of 3’,4’,5’-trimethoxyflavone 6-*O*-β-D-(4”-*O*-methyl)-glucopyranoside (**21**) (DMSO-$d_6$, 600 MHz)

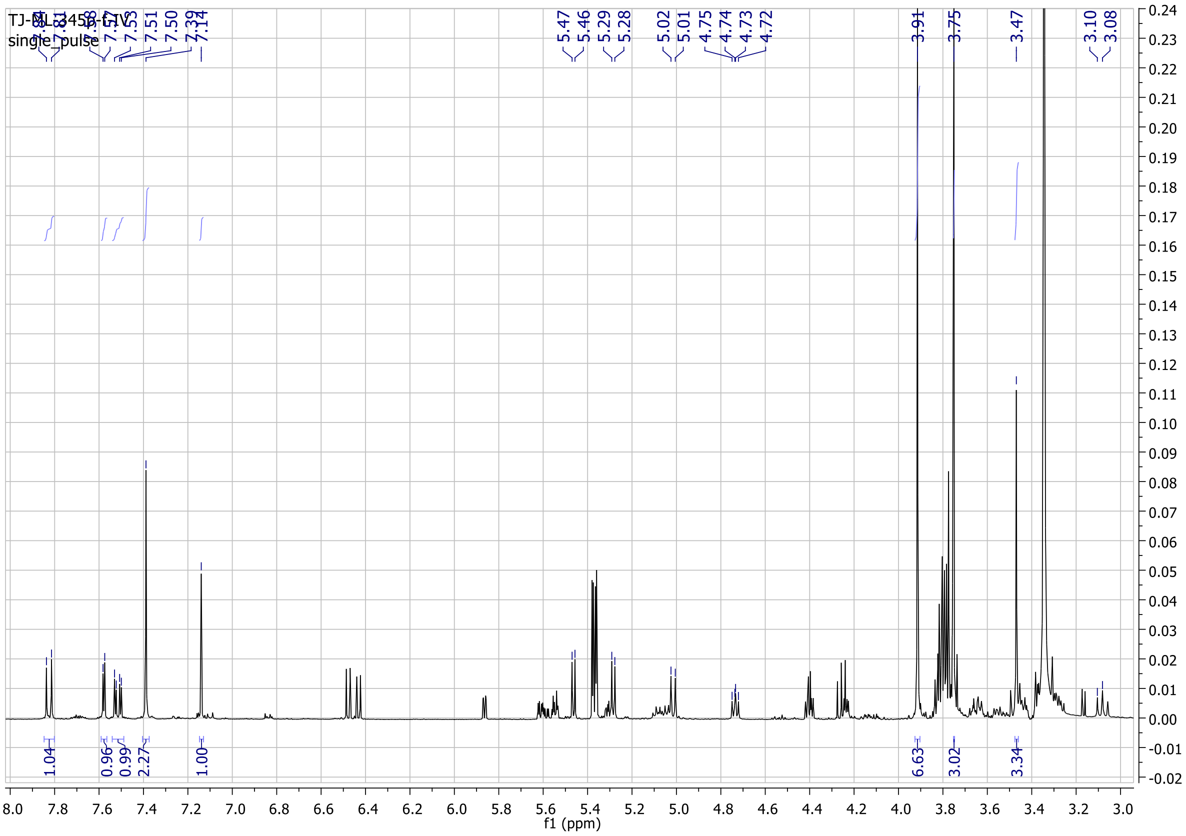


Fig.S98. Flavone part of the $^{1}$H NMR spectral 3’,4’,5’-trimethoxyflavone 6-*O*-β-D-(4”-*O*-methyl)-glucopyranoside (**21**) (DMSO-$d_6$, 600 MHz)

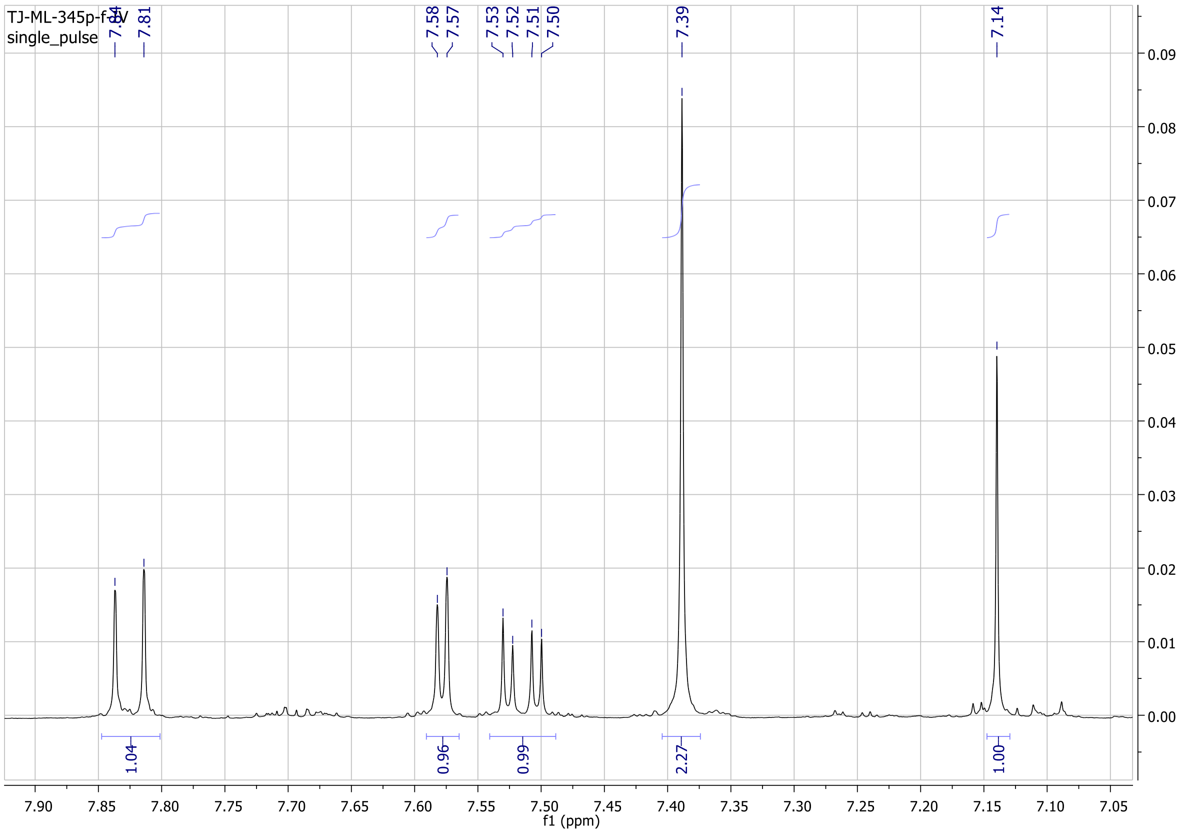

Fig.S99. Glucopyranoside part of the $^{1}$H NMR spectral 3’,4’,5’-trimethoxyflavone 6-*O*-β-D-(4”-*O*-methyl)-glucopyranoside (**21**) (DMSO-$d_6$, 600 MHz)

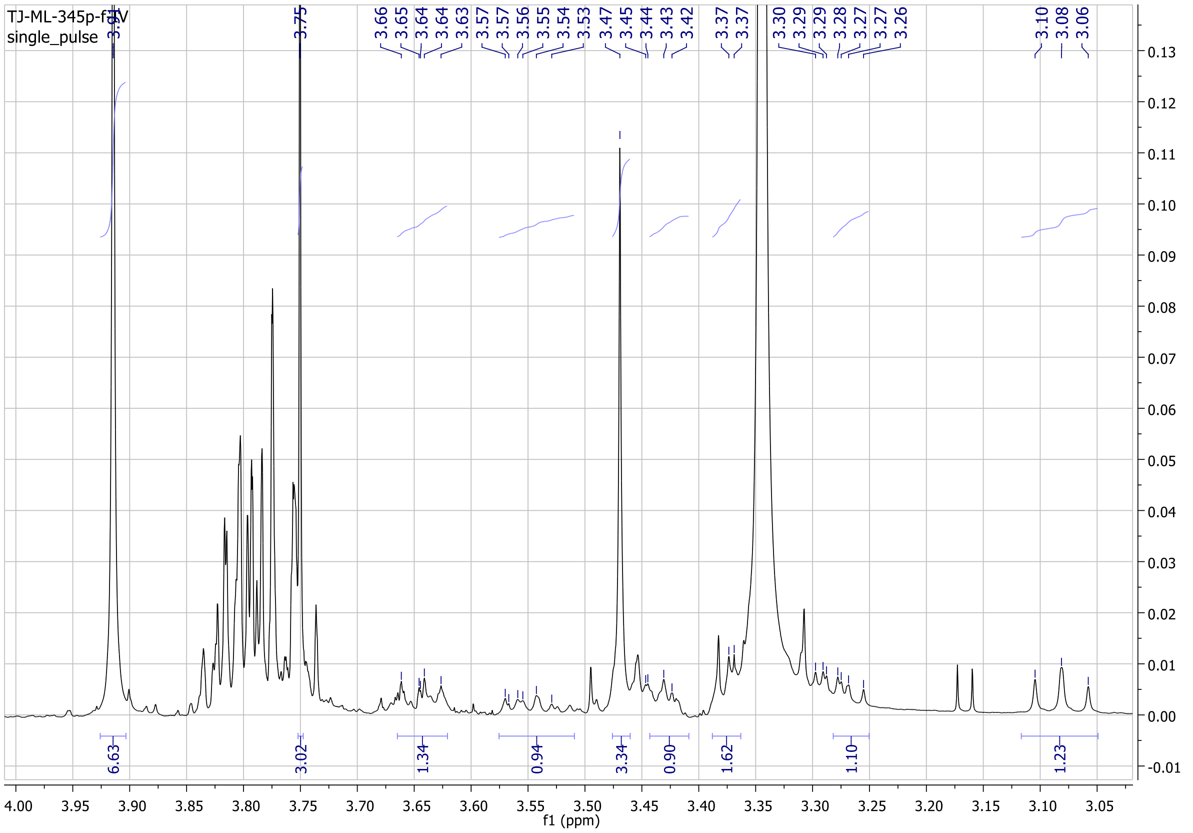


Fig.S100. $^{13}$C NMR spectral of 3’,4’,5’-trimethoxyflavone 6-*O*-β-D-(4”-*O*-methyl)-glucopyranoside (**21**) (DMSO-$d_6$, 151 MHz)

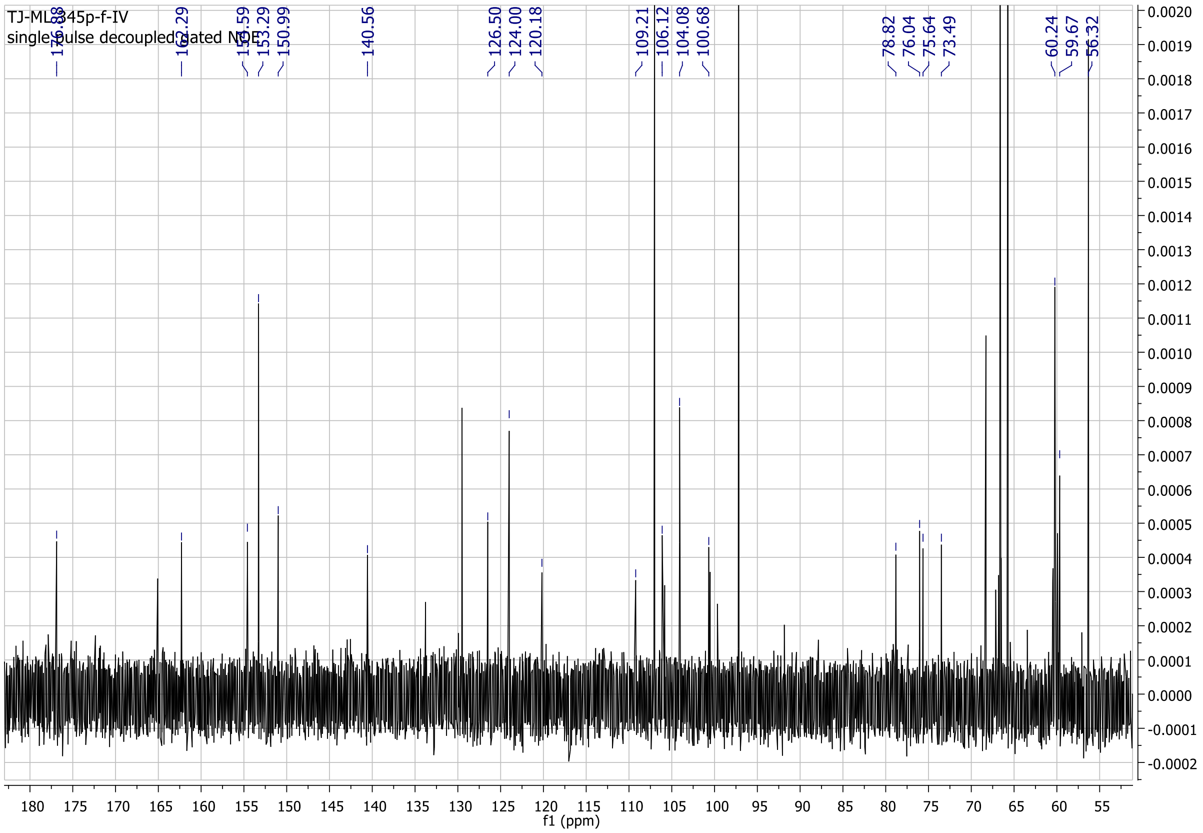

Fig.S101. HMQC spectral of 3’,4’,5’-trimethoxyflavone 6-*O*-β-D-(4”-*O*-methyl)-glucopyranoside (**21**) (DMSO-$d_6$, 151 MHz)

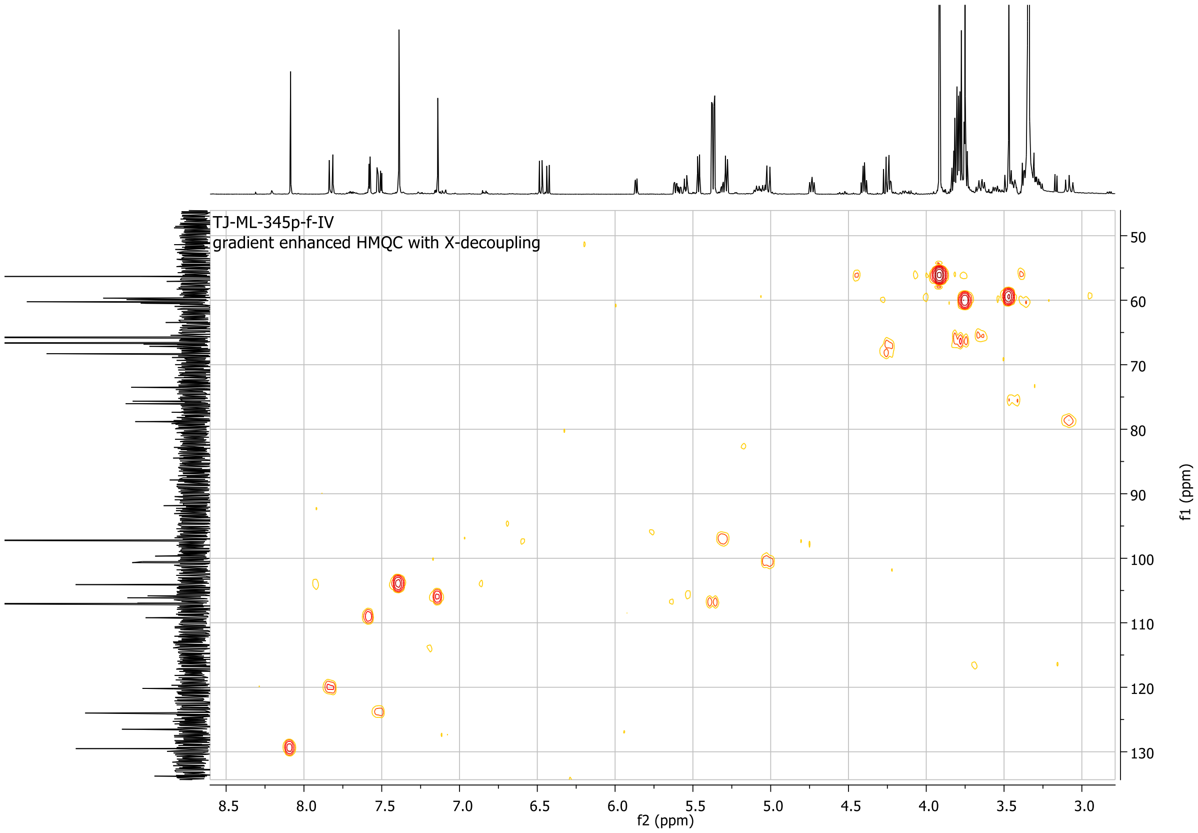


Fig.S102. HMBC spectral of 3’,4’,5’-trimethoxyflavone 6-*O*-β-D-(4”-*O*-methyl)-glucopyranoside (**21**) (DMSO-$d_6$, 151 MHz)

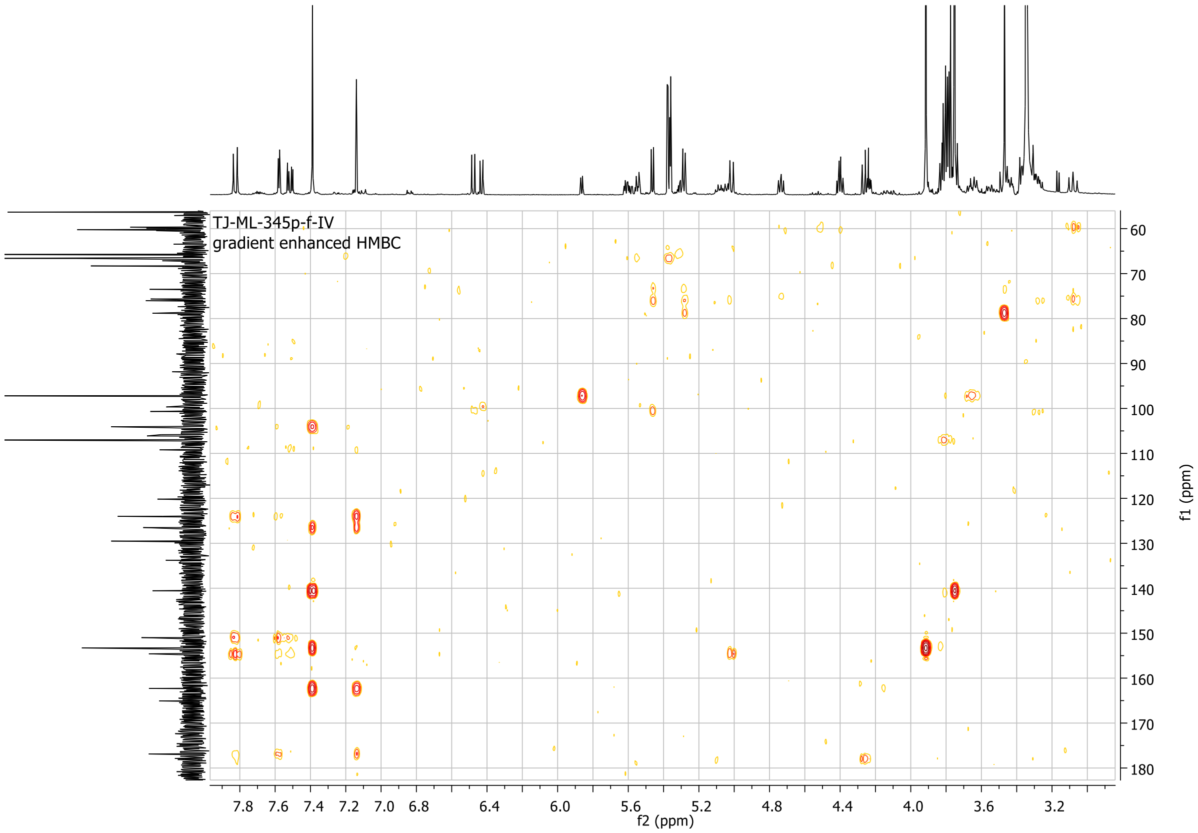

Fig.S103. MS analysis 6-hydroxy-4’,5’-dimethoxyflavone 3’-*O*-β-D-(4”-*O*-methyl)-glucopyranoside (**22**)

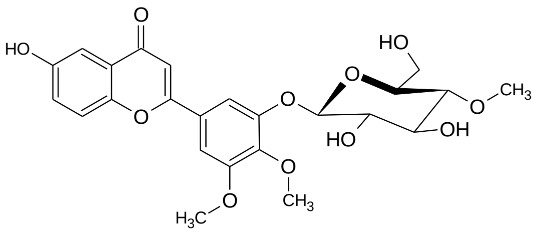


Molecular Formula = $C_{24}H_{26}O_{11}$
Formula Weight = 490.45664
Precursor: = 491.4000

CE:-15

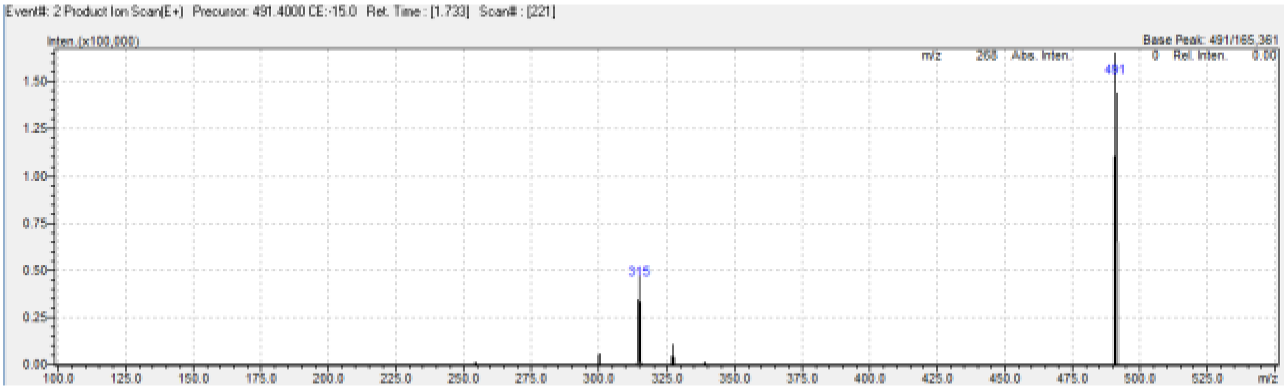


CE:-35

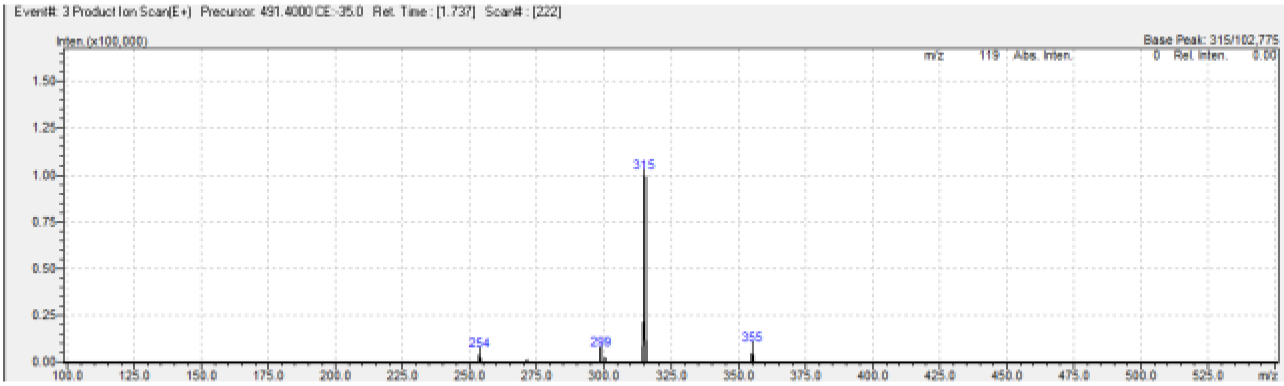


CE:-45

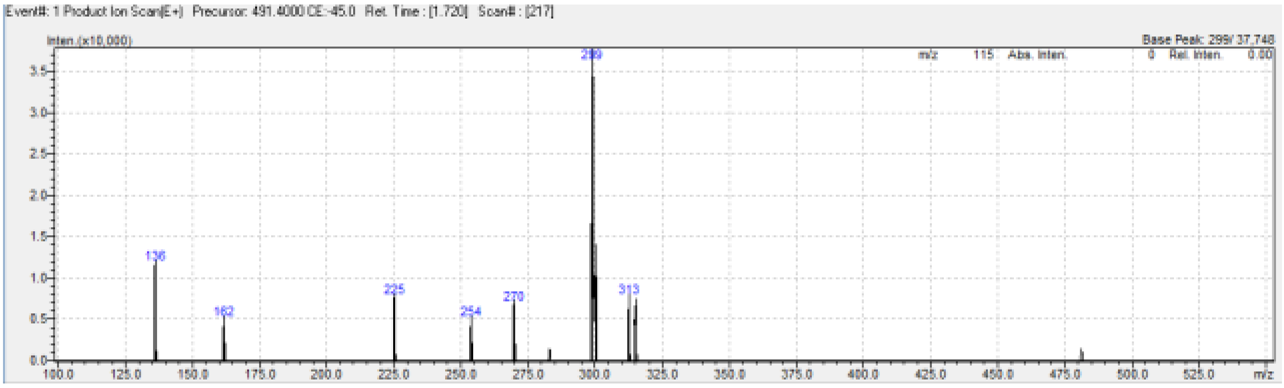

Fig.S104. $^{1}$H NMR spectral of 6-hydroxy-4’,5’-dimethoxyflavone 3’-*O*-β-D-(4”-*O*-methyl)-glucopyranoside (**22**) (DMSO-$d_6$, 600 MHz)

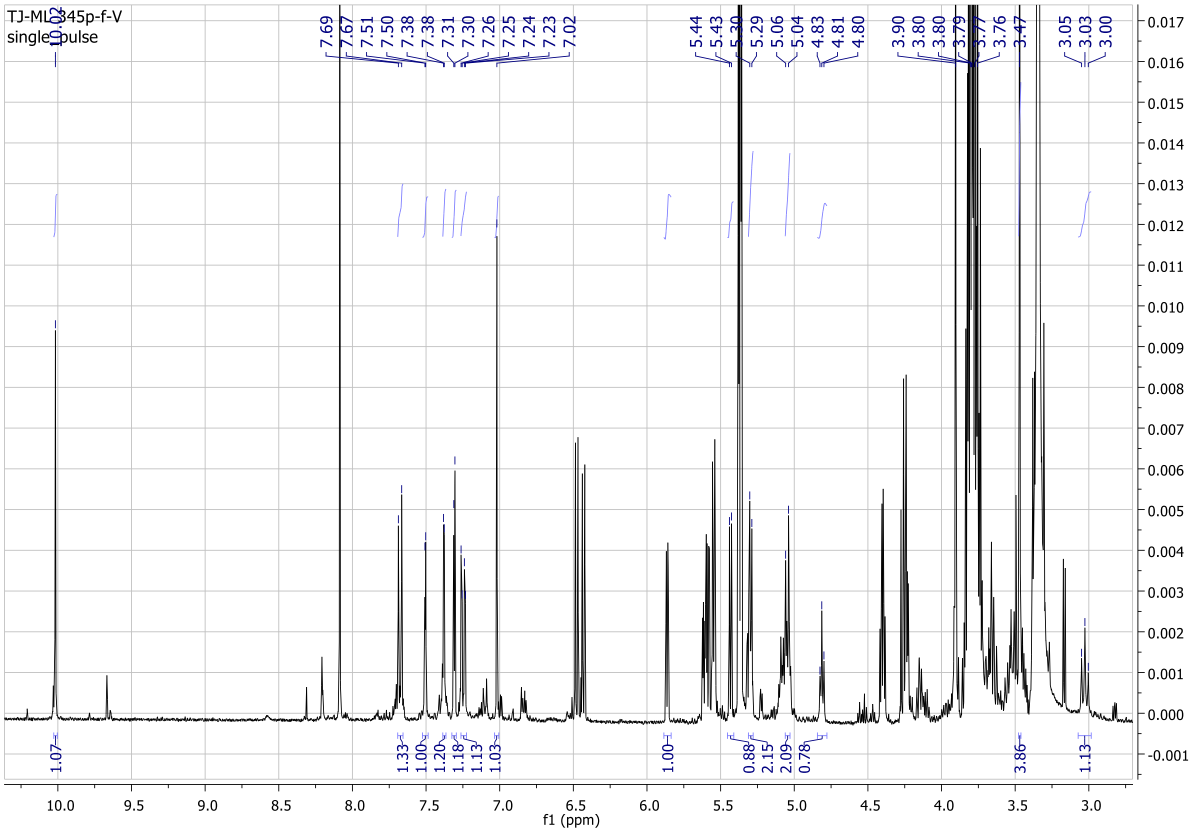

Fig.S105. Flavone part of the $^{1}$H NMR spectral 6-hydroxy-4’,5’-dimethoxyflavone 3’-*O*-β-D-(4”-*O*-methyl)-glucopyranoside (**22**) (DMSO-$d_6$, 600 MHz)

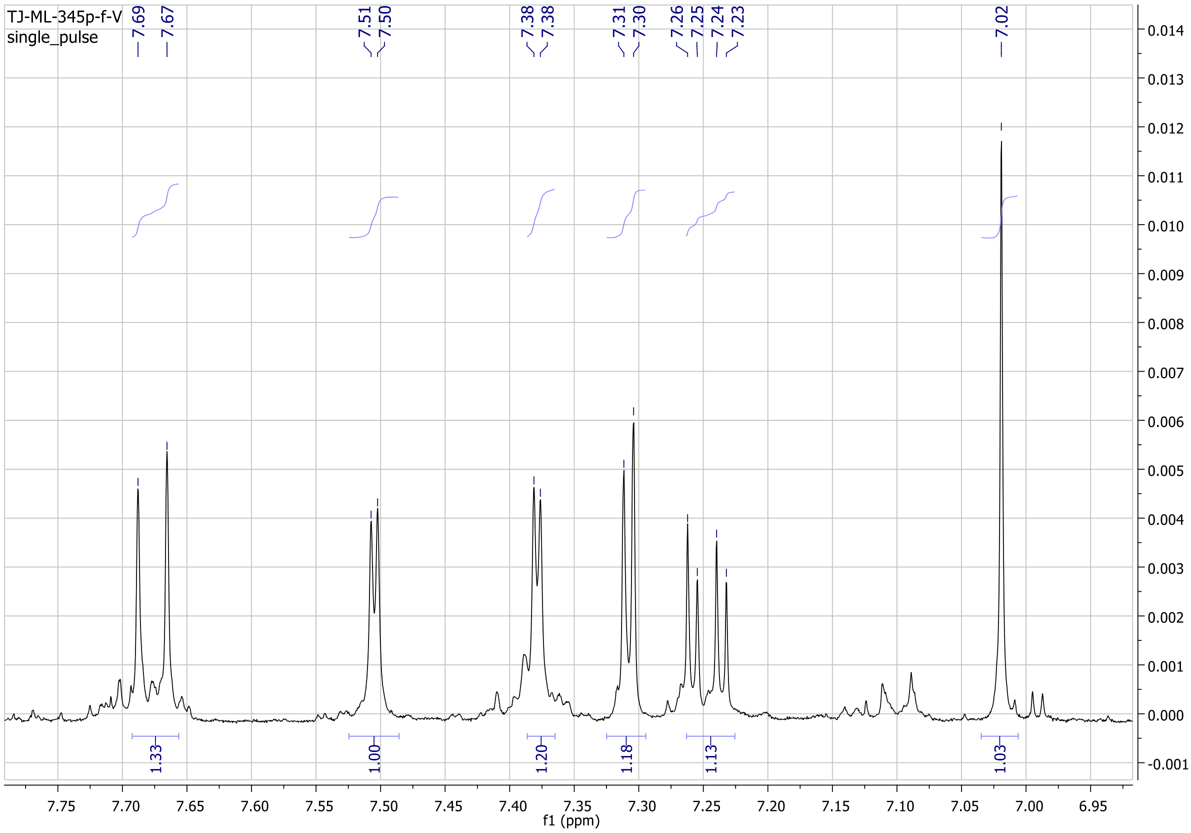

Fig.S106. Glucopyranoside part of the $^{1}$H NMR spectral 6-hydroxy-4',5'-dimethoxyflavone 3'-*O*-β-D-(4"-*O*-methyl)-glucopyranoside (**22**) (DMSO-$d_6$, 600 MHz)

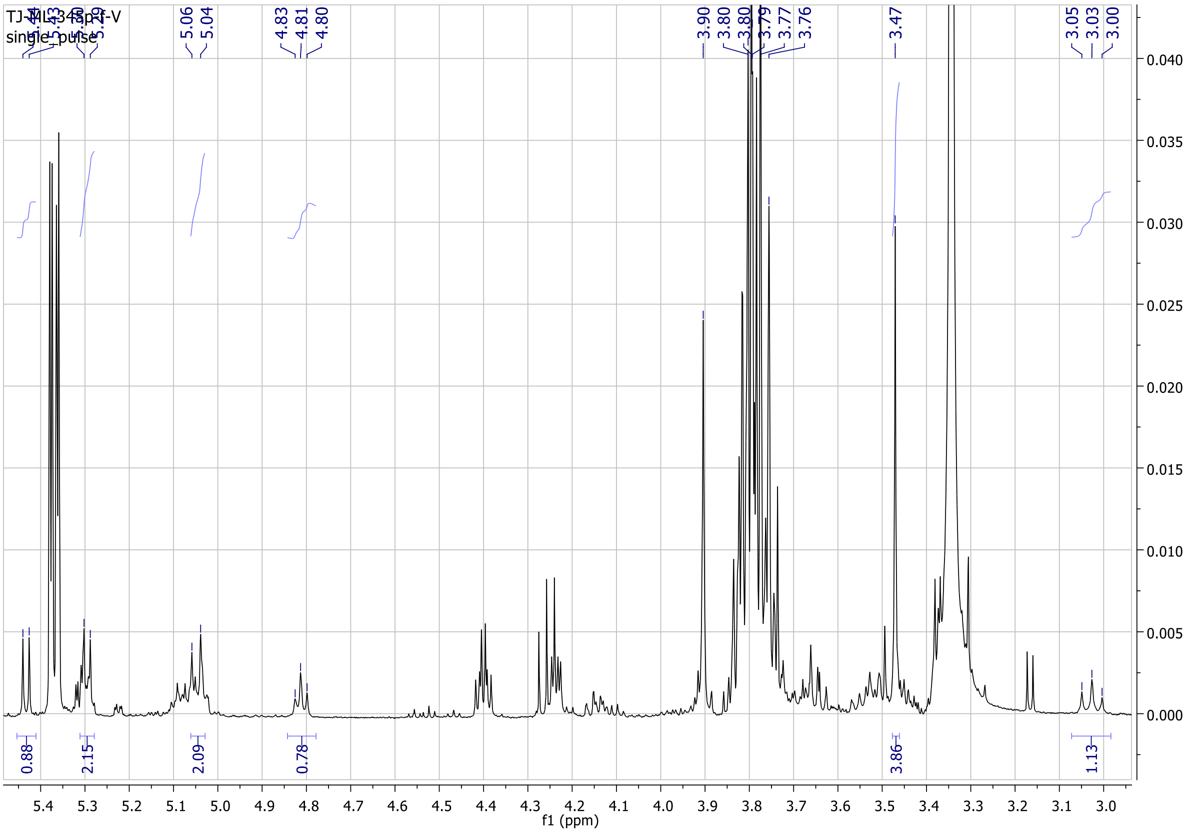


Fig.S107. $^{13}$C NMR spectral of 6-hydroxy-4',5'-dimethoxyflavone 3'-*O*-β-D-(4"-*O*-methyl)-glucopyranoside (**22**) (DMSO-$d_6$, 151 MHz)

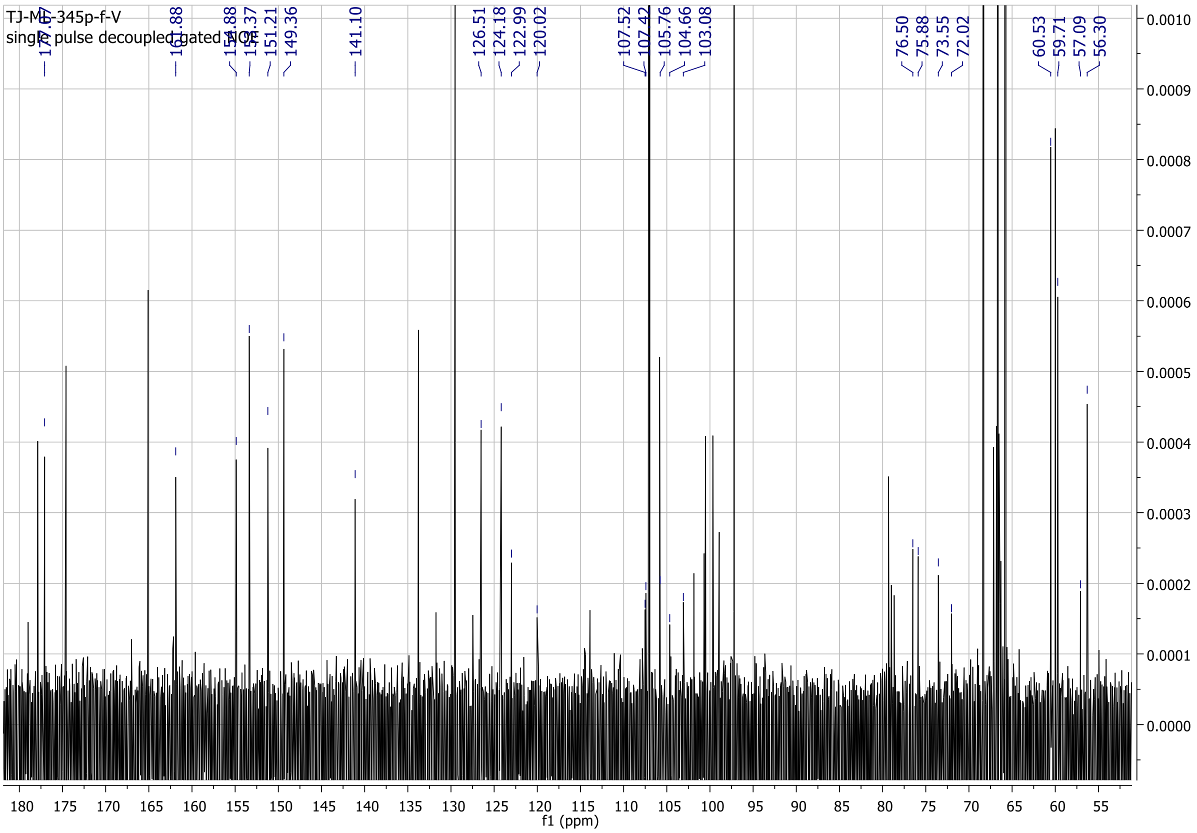

Fig.S108. HMQC spectral of 6-hydroxy-4',5'-dimethoxyflavone 3'-*O*-β-D-(4''-*O*-methyl)-glucopyranoside (**22**) (DMSO-$d_6$, 151 MHz)

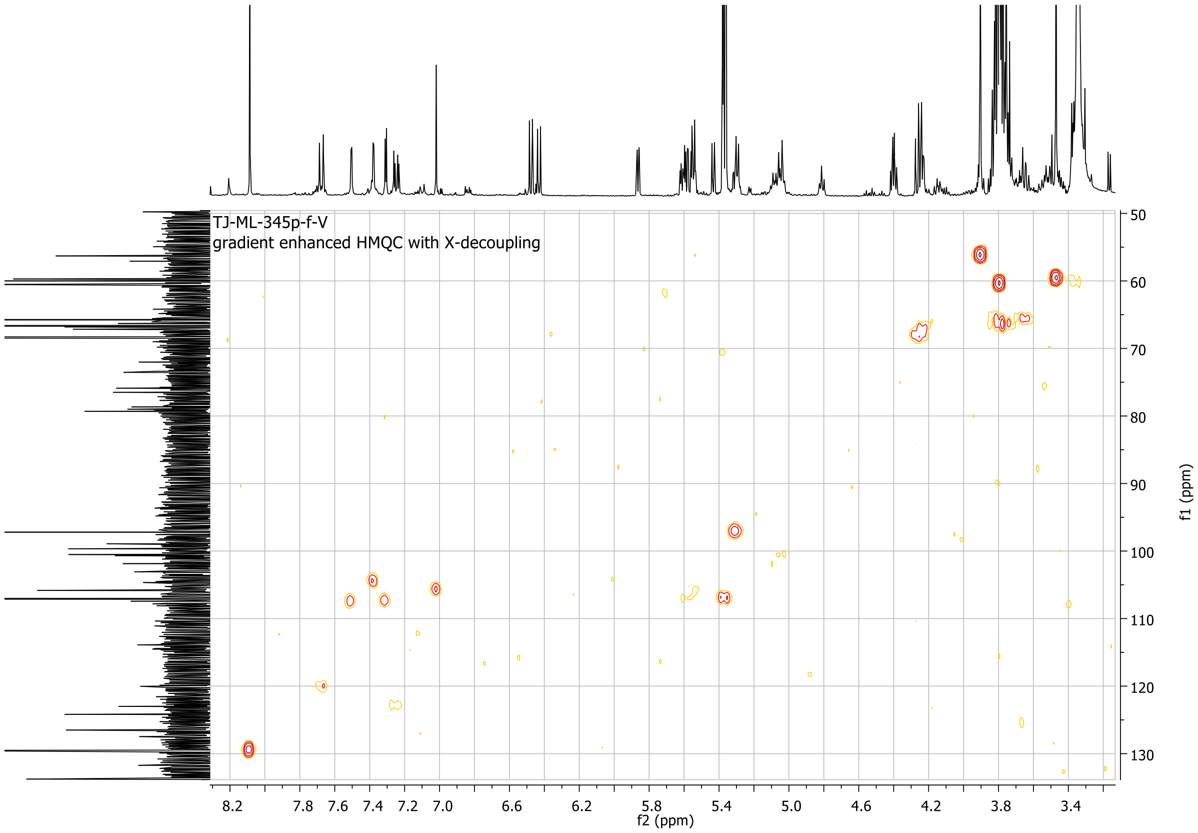


Fig.S109. HMBC spectral of 6-hydroxy-4',5'-dimethoxyflavone 3'-*O*-β-D-(4''-*O*-methyl)-glucopyranoside (**22**) (DMSO-$d_6$, 151 MHz)

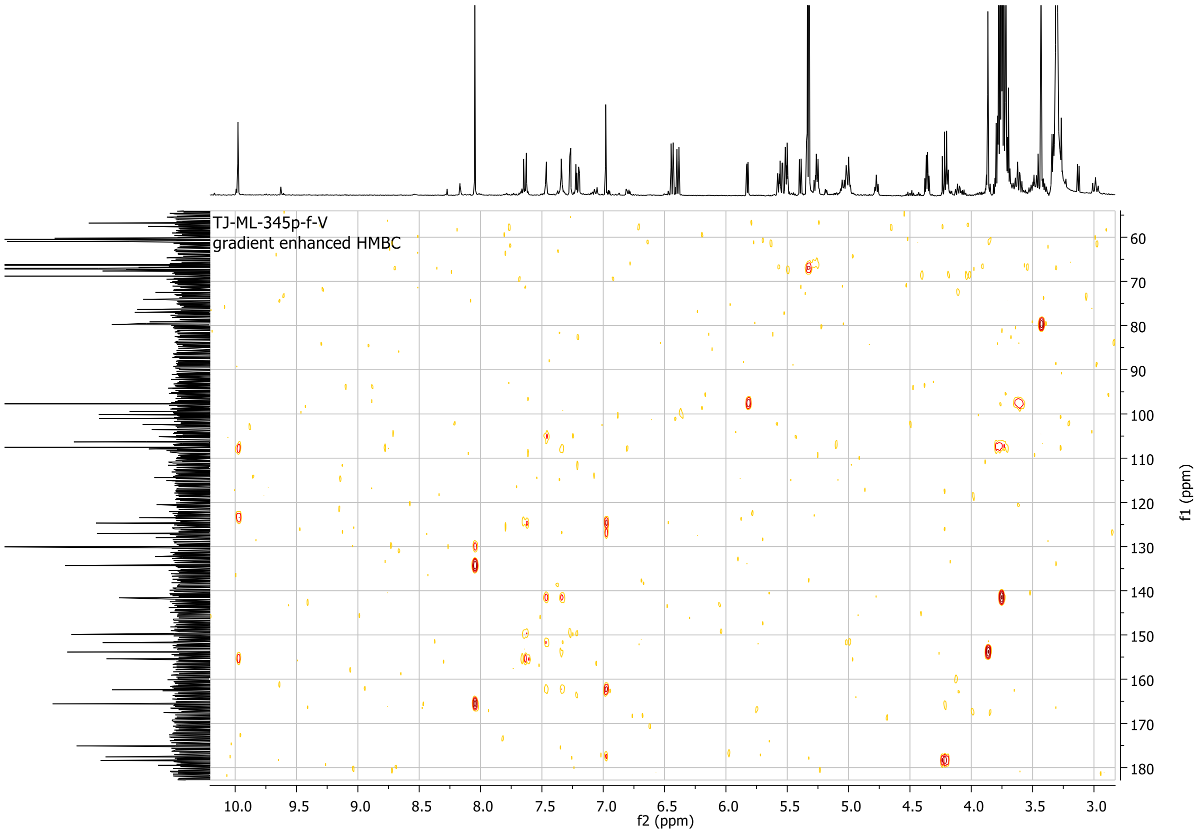

Supplement: Supplementary file 1 [file ijms-21-06121-s001.pdf]
